# Synthesis of 2′-formamidonucleoside phosphoramidites for suppressing the seed-based off-target effects of siRNAs

**DOI:** 10.1093/nar/gkae741

**Published:** 2024-09-05

**Authors:** Kohei Nomura, Seongjin An, Yoshiaki Kobayashi, Jiro Kondo, Ting Shi, Hirotaka Murase, Kosuke Nakamoto, Yasuaki Kimura, Naoko Abe, Kumiko Ui-Tei, Hiroshi Abe

**Affiliations:** Department of Chemistry, Graduate School of Science, Nagoya University, Furo-cho, Chikusa-ku, Nagoya, Aichi 464-8602, Japan; Department of Computational Biology and Medical Sciences, Graduate School of Frontier Sciences, The University of Tokyo, Chiba 277-8561, Japan; Department of Biological Sciences, Graduate School of Science, The University of Tokyo, Tokyo 113-0033, Japan; Department of Materials and Life Sciences, Faculty of Science and Technology, Sophia University, 7-1 Kioi-cho, Chiyoda-ku 102-8554 Tokyo, Japan; Department of Chemistry, Graduate School of Science, Nagoya University, Furo-cho, Chikusa-ku, Nagoya, Aichi 464-8602, Japan; Department of Chemistry, Graduate School of Science, Nagoya University, Furo-cho, Chikusa-ku, Nagoya, Aichi 464-8602, Japan; Department of Chemistry, Graduate School of Science, Nagoya University, Furo-cho, Chikusa-ku, Nagoya, Aichi 464-8602, Japan; Department of Chemistry, Graduate School of Science, Nagoya University, Furo-cho, Chikusa-ku, Nagoya, Aichi 464-8602, Japan; Department of Chemistry, Graduate School of Science, Nagoya University, Furo-cho, Chikusa-ku, Nagoya, Aichi 464-8602, Japan; Department of Computational Biology and Medical Sciences, Graduate School of Frontier Sciences, The University of Tokyo, Chiba 277-8561, Japan; Department of Biological Sciences, Graduate School of Science, The University of Tokyo, Tokyo 113-0033, Japan; Department of Chemistry, Graduate School of Science, Nagoya University, Furo-cho, Chikusa-ku, Nagoya, Aichi 464-8602, Japan; Research Center for Materials Science, Nagoya University, Furo-cho, Chikusa-ku, Nagoya, Aichi 464-8602, Japan; CREST, Japan Science and Technology Agency, 7 Gobancho, Chiyoda-ku, Tokyo 102-0076, Japan; Institute for Glyco-core Research (iGCORE), Nagoya University, Furo-cho, Chikusa-ku, Nagoya, Aichi464-8601, Japan

## Abstract

In this study, we report the synthesis of 2′-formamidonucleoside phosphoramidite derivatives and their incorporation into siRNA strands to reduce seed-based off-target effects of small interfering RNAs (siRNAs). Formamido derivatives of all four nucleosides (A, G, C and U) were synthesized in 5–11 steps from commercial compounds. Introducing these derivatives into double-stranded RNA slightly reduced its thermodynamic stability, but X-ray crystallography and CD spectrum analysis confirmed that the RNA maintained its natural A-form structure. Although the introduction of the 2′-formamidonucleoside derivative at the 2nd position in the guide strand of the siRNA led to a slight decrease in the on-target RNAi activity, the siRNAs with different sequences incorporating 2′-formamidonucleoside with four kinds of nucleobases into any position other than 2nd position in the seed region revealed a significant suppression of off-target activity while maintaining on-target RNAi activity. This indicates that 2′-formamidonucleosides represent a promising approach for mitigating off-target effects in siRNA therapeutics.

## Graphical Abstract

**Graphical Abstract:**
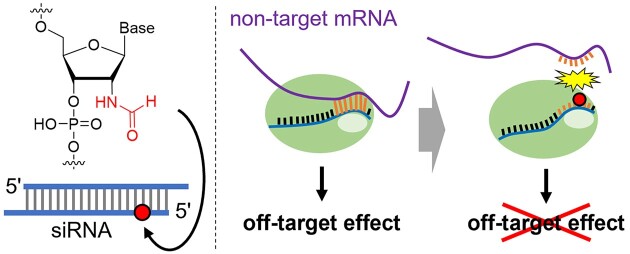

## Introduction

**Figure 1.**
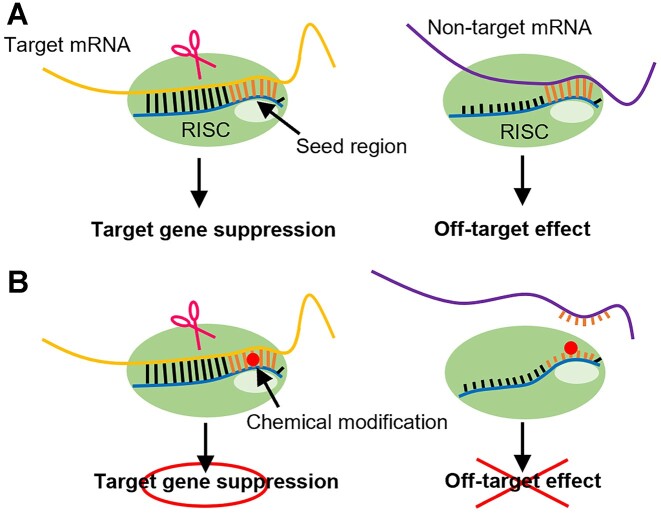
Off-target effect caused by the seed match sequence. (**A**) Mechanism of on- and off-target effects. Target gene suppression is caused by a full-match siRNA guide strand. Seed-match siRNA guide strands bind to non-target mRNA only in the seed region and cause off-target effects. (**B**) Effect of introducing chemical modifications in the seed region. Full-match siRNA guide strands can cause target gene suppression even if chemical modification is introduced into the seed region. Seed-matched siRNA guide strands cannot bind non-target mRNA because of chemical modification, and the off-target effect is suppressed.

Small interfering RNAs (siRNAs) are composed of two strands: a guide strand and a passenger strand. The guide strand forms a complex with proteins known as the RNA-induced silencing complex (RISC) in cells (1–3). Subsequently, one of the RISC component proteins, Argonaute2 (Ago2), cleaves messenger RNA (mRNA) that has a complementary sequence to the guide strand, thereby suppressing the expression of this gene through a process known as RNA interference (RNAi) (1,4). The gene-silencing effect of siRNAs has been utilized in the treatment of diseases, and several pharmaceuticals employing this technology have already been marketed (5–9). However, certain siRNA sequences may have off-target effects, including the suppression of non-targeted mRNA expression. Off-target effects of siRNAs can be broadly categorized into two types. The first involves the unintended strand of the double-stranded RNA becoming the guide strand (10,11). Molecular design of siRNAs has been established to ensure that the desired strand acts as a guide (12–15). The second type is binding of the seed region of the guide strand to non-target sequences. Specifically, seven nucleotides from the 2nd to 8th position from the 5′ end of the siRNA guide strand, known as the seed region, are crucial for target recognition. If this seed region sequence can form base pairs with a non-target mRNA, the guide strand may bind to the mRNA solely through the seed region, resulting in off-target effects (Figure 1A) (16,17). This mechanism is similar to how microRNAs (miRNAs) suppress gene expression (18). These undesired off-target effects may be mitigated by designing siRNA molecules such that the interaction between the seed region of the guide strand in RISC and non-target mRNA is destabilized, while ensuring that a stable complex is formed only when the entire guide strand, typically approximately 21 nucleotides in length, binds to the mRNA.

Achieving the aforementioned siRNA molecular design strategy using only natural RNA structures is challenging (19). To date, the introduction of chemically modified nucleotides into the seed region has been linked to a reduction in off-target effects (Figure 1B). For instance, modifications in the seed region such as DNA (20), unlocked nucleic acids (UNA) (21), spacer amidites (21) (22), 2′-*O*-methyl (2′-OMe) (23,24), locked nucleic acids (LNA) (24), 1-triazole derivatives (25), glycol nucleic acids (GNA) (26), 2′-5′-RNA (27), and replacement of the internucleoside phosphates with amide linkages (28) have demonstrated efficacy in suppressing off-target effects. Modified nucleotides with lower melting temperatures (*T*_m_) than natural RNA, such as DNA (29), UNA (30), spacer amidite, GNA (31), and 2′-5′-RNA (32) inhibit the formation of the RISC complex in the unwanted seed region in a *T*_m_-dependent manner. Conversely, modifications with a higher *T*_m_ than natural RNA, such as 2′-OMe (33) and LNA (34), are expected to bind non-target mRNA more strongly in the seed region, leading to lower selectivity; however, reports suggest that they reduce off-target effects. This phenomenon can be explained by the fact that chemically modified nucleic acids destabilize the complex formation between the non-target mRNA and the RISC in the seed region. While the destabilization factor is thought to be due to local steric hindrance (24). However, multiple modifications are necessary to achieve sufficient suppression of off-target effects by 2′-OMe RNAs and LNAs, which can lead to a reduction in on-target RNAi activity (24). The amide linker improves target specificity by introducing a modification at one position. Since this modification has no effect on the duplex stability, it may destabilize the interaction with mRNA in the seed region through a conformational change on the Ago protein (28), similar to 2′-OMe and LNA. However, incorporating the linker modification necessitates the use of dimeric phosphoramidites, and 16 different amidites are required to cover all sequences. It has been noted that certain base combinations among the 16 may pose challenges during the synthesis process (28).

Designing modified nucleotides that reduce the *T*_m_ in double-stranded formation and locally destabilize the RISC complex in the seed region due to steric factors is anticipated to demonstrate high efficacy with a single modification and increase the flexibility of the molecular design. Using only common existing modified nucleosides has limitations, so developing novel chemical modifications of nucleosides from a chemical perspective may lead to the advancement of more useful chemically modified siRNAs. In this study, we designed and synthesized 2′-formamidonucleoside phosphoramidites and incorporated them into siRNA strands. We evaluated their on/off-target effects in RNAi and reported the utility of 2′-formamidonucleosides.

## Materials and methods

### General

All commercial starting materials, reagents and solvents were purchased as reagent-grade, first-grade or special-grade from TCI, FUJIFILM Wako, KANTO CHEMICAL and Sigma-Aldrich unless otherwise stated. All solvents for the reactions were purchased as super dehydrated grade from FUJIFILM Wako. All the purchased materials were used without further purification. The sterile water used in this study was produced by MicroPure UV (Thermo SCIENTIFIC) and Pacific TII 3 UV (Thermo SCIENTIFIC). Silicagel 70 F254 TLC Plate-Wako was used for TLC. Silica Gel 60 (spherical) 40–50 μm (KANTO CHEMICAL) was used for silica gel column chromatography. All reactions were performed under argon atmosphere unless otherwise stated. The reactions at elevated temperatures were performed using a temperature-controlled oil bath. The NMR spectra were measured using a JNM-ECS400 spectrometer (JEOL). The chemical shift values of the ^1^H-NMR spectra were adjusted using the residual proton of the solvent (chloroform: 7.26 ppm, DMSO: 2.50 ppm) as an internal standard. The chemical shift values of the ^13^C-NMR spectra were adjusted using signals from the measured solvents (chloroform: 77.16 ppm, DMSO: 39.52 ppm) as an internal standard. The chemical shift values of the ^19^F-NMR and ^31^P-NMR spectra were not standardized. The multiplicity of the signal is expressed as s: singlet, d = doublet, t = triplet, q = quartet, sext = sextet, sep = septuplet, m = multiplet, and br = broad singlet. HRMS of the compounds was performed using a compact (Bruker) (ESI-MS).

### Synthesis of 2′-formamidonucleoside phosphoramidites

*2, 2′-O-Anhydro-5′-O- (4, 4′-dimethoxytrityl)uridine (* ***2*** *)* (35). 2, 2′-*O*-Cyclouridine (**1**) (5.00 g, 22.1 mmol, 1 eq.) were dissolved in pyridine (50 ml). DMTrCl (8.24 g, 24.3 mmol, 1.1 eq.) and DMAP (0.0540 g, 0.442 mmol, 0.02 eq.) was then added, and the mixture was stirred at room temperature. After stirring for 16 h, the reaction mixture was diluted with AcOEt and washed with a saturated NaHCO_3_ aqueous solution and brine. The organic layer was dried over Na_2_SO_4_, filtered, and concentrated *in vacuo*. The crude product was purified by silica gel column chromatography (AcOEt/MeOH = 10/1), and compound **2** (10.4 g, 19.8 mmol, 90%) was obtained. ^1^H-NMR (400 MHz, DMSO-*d*_6_): δ 7.944 (d, *J* = 7.6 Hz, 1H), 7.292–7.115 (m, 9H), 6.860–6.808 (m, 4H), 6.325 (d, *J* = 5.6 Hz, 1H), 5.976 (br, 1H), 5.880 (d, *J* = 7.6 Hz, 1H), 5.207 (dd, *J* = 0.8, 5.6 Hz, 1H), 4.300 (m, 1H), 4.233–4.197 (m, 1H), 3.730 (s, 6H), 2.937 (dd, *J* = 10.4, 4.4 Hz, 1H), 2.808 (dd, *J* = 10.4, 7.4 Hz, 1H); ^13^C-NMR (100 MHz, DMSO-*d*_6_): δ 170.962, 159.303, 158.067, 144.683, 136.789, 135.218, 129.565, 127.889, 127.467, 126.720, 113.260, 108.920, 89.797, 88.466, 86.885, 85.410, 74.756, 62.848, 55.002; HRMS (ESI-TOF): calcd. for C_30_H_28_N_2_O_7_ [M + Na]^+^ 551.1789, found 551.1792.

*2′-Azido-2′-deoxy-5′-O-(4,4′-dimethoxytrityl)uridine (* ***3*** *)* (35). Compound **2** (10.0 g, 18.9 mmol, 1 equiv.) was dissolved in dimethylformamide (DMF; 62.5 ml). Sodium azide (6.5 g, 100 mmol, 5 equiv.) and 15-crown-5 (37.5 ml, 189.5 mmol, 10 eq.) was added, and the mixture was stirred at 120°C. After stirring for 42 h, the reaction mixture was diluted with AcOEt and washed with a saturated NaHCO_3_ aqueous solution and brine. The organic layer was dried over Na_2_SO_4_, filtered, and concentrated *in vacuo*. The crude product was purified using silica gel column chromatography (hexane/AcOEt = 1/2), and compound **3** (7.01 g, 12.3 mmol, 65%) was obtained. ^1^H-NMR (400 MHz, CDCl_3_): δ 8.946 (br, 1H), 7.906 (d, *J* = 8.4 Hz, 1H), 7.376–7.226 (m, 9H), 6.847 (d, *J* = 4.4 Hz, 4H), 5.976 (d, *J* = 2.8 Hz, 1H), 5.377 (d, *J* = 8.0 Hz, 1H), 4.487 (q, *J* = 6.4 Hz, 1H), 4.172 (q, *J* = 2.8 Hz, 1H), 4.049–4.021 (m, 1H), 3.795 (s, 6H), 3.601 (dd, *J* = 11.4, 2.2 Hz, 1H), 3.486 (dd, *J* = 11.4, 2.6 Hz, 1H), 2.536 (d, *J* = 6.8 Hz, 1H); ^13^C-NMR (100 MHz, CDCl_3_): δ 163.112, 158.915, 150.179, 144.304, 139.659, 135.253, 135.060, 130.167, 128.164, 127.363, 113.448, 102.527, 87.811, 87.382, 83.290, 69.823, 67.267, 61.421, 55.365; HRMS (ESI-TOF) calcd. for C_30_H_29_N_5_O_7_ [M + Na]^+^ 594.1959, found 594.1940.

*2′-Amino-2′-deoxy-5′-O- (4, 4′-dimethoxytrityl)uridine (* ***4*** *)* (35). Compound **3** (6.50 g, 11.4 mmol) was then dissolved in methanol (115 ml). Pd: carbon powder (Pd: 10%; H_2_O: 55.64%; N. E. CHEMCAT) (0.95 g) was added and stirred at room temperature with H_2_ bubbling. After stirring for 2.5 h, the reaction mixture was filtered using Cerite and concentrated *in vacuo*. Compound **4** (5.66 g, 10.4 mmol, 91% yield) was obtained. ^1^H-NMR (400 MHz, CDCl_3_): δ 7.802 (d, *J* = 8.4 Hz, 1H), 7.387–7.223 (m, 9H), 6.866–6.819 (m, 4H), 5.903 (d, *J* = 6.4 Hz, 1H), 5.425 (d, *J* = 8.4 Hz, 1H), 4.236–4.204 (m, 2H), 3.799 (s, 6H), 3.604 (t, *J* = 6.0 Hz, 1H), 3.496–3.404 (m, 2H); ^13^C-NMR (100 MHz, CDCl_3_): δ 163.216, 158.848, 151.247, 144.370, 140.069, 135.405, 135.253, 130.188, 128.185, 127.308, 113.460, 102.568, 89.988, 87.232, 85.534, 71.743, 63.521, 59.687, 55.405; HRMS (ESI-TOF) calcd. for C_30_H_31_N_3_O_7_ [M + Na]^+^ 568.2054, found 568.2074.

*2′-Deoxy-5′-O- (4,4′-dimethoxytrityl)-2′-formamidouridine (* ***5*** *)*. Compound **4** (5.50 g, 10.1 mmol, 1 equiv.) were dissolved in dichloromethane (75 ml). DIPEA (26.4 ml, 152 mmol, 15 eq.), EDC·HCl (2.90 g, 15.1 mmol, 1.5 eq.), DMAP (0.0620 g, 0.507 mmol, 0.05 eq.), and formic acid (0.571 ml, 15.1 mmol, 1.5 eq.) was then added, and the mixture was stirred at room temperature. After stirring for 19 h, the reaction mixture was washed with a saturated NaHCO_3_ aqueous solution and brine. The organic layer was dried over Na_2_SO_4_, filtered, and concentrated *in vacuo*. The crude product was purified by silica gel column chromatography (CH_2_Cl_2_/MeOH = 10/1) and compound **5** (3.30 g, 5.75 mmol, 57%) was obtained. ^1^H-NMR (400 MHz, CDCl_3_): δ 8.197 (s, 1H), 7.620 (d, *J* = 8.0 Hz, 1H), 7.555 (d, *J* = 7.2 Hz, 1H), 7.379–7.147 (m, 9H), 6.830–6.808 (m, 4H), 6.115 (d, *J* = 8.8 Hz, 1H), 5.405 (d, *J* = 8.0 Hz, 1H), 4.773 (dd, *J* = 8.4 Hz, *J* = 14 Hz, 1H), 4.388 (d, *J* = 5.2 Hz, 1H), 4.174 (m, 1H), 3.729 (s, 6H), 3.376 (m, 2H); ^13^C-NMR (100 MHz, CDCl_3_): δ 163.642, 163.201, 158.775, 151.982, 144.193, 140.055, 135.389, 135.226, 130.245, 128.309, 127.284, 113.479, 103.525, 87.248, 85.639, 85.572, 71.833, 63.863, 55.374, 55.039; HRMS (ESI-TOF) calcd. for C_31_H_31_N_3_O_8_ [M + Na]^+^ 596.2003, found 596.2026.

*2′-Deoxy-5′-O-(4,4′-dimethoxytrityl)-2′-formamidouridine-3′-O-(2-cyanoethyl N,N-diisopropylphosphoramidite) (* ***6*** *)* (36). Compound **5** (3.00 g, 5.23 mmol, 1 eq.) were co-evaporated with toluene and dissolved in dichloromethane (55 ml). DIPEA (5.50 ml, 31.6 mmol, 2.5 eq.) and 2-cyanoethyl-*N*,*N*-diisopropylchlorophosphoroamidite (2.90 ml, 13.0 mmol, 2.5 eq.) was added at 0°C, and the mixture was stirred at 0°C. After stirring for 3 h, the reaction mixture was diluted with AcOEt and washed with a saturated NaHCO_3_ aqueous solution and brine. The organic layer was dried over Na_2_SO_4_, filtered, and concentrated *in vacuo*. The crude product was purified by silica gel column chromatography (hexane/AcOEt/TEA = 9/90/1→0/99/1), and compound **6** (1.16 g, 1.50 mmol, 29%) was obtained. ^1^H-NMR (400 MHz, CDCl_3_): δ 8.279 (s, 1H), 7.647 (d, *J* = 8.4 Hz, 1H), 7.416–7.220 (m, 9H), 6.869–6.830 (m, 4H), 6.614 (d, *J* = 8.8 Hz, 1H), 6.115 (d, *J* = 8.8 Hz, 1H), 5.460, (d, *J* = 8.0 Hz, 1H), 4.922–4.865 (m, 1H), 4.587 (q, *J* = 5.6 Hz, 1H), 4.227 (m, 1H), 3.919–3.834 (m, 1H), 3.795 (s, 6H), 3.778–3.703 (m, 1H), 3.659–3.564 (m, 2H), 3.404 (d, *J* = 3.2 Hz, 2H), 2.649 (t, *J* = 6.0 Hz, 2H), 1.195–1.121 (m, 12H); ^13^C-NMR (100 MHz, CDCl_3_): δ 162.711, 161.986, 158.886, 150.942, 144.065, 140.050, 135.262, 135.091, 130.226, 128.252, 127.346, 117.790, 113.517, 103.140, 87.489, 86.211, 84.990, 75.109, 63.569, 58.457, 55.424, 53.822, 43.579, 24.647, 20.631; ^31^P-NMR (160 MHz, CDCl_3_): δ 151.932; HRMS (ESI-TOF) calcd. for C_40_H_48_N_5_O_9_P [M + Na]^+^ 796.3082, found 796.3089.

*2′-Azide-2′-deoxy-3′,5′-di-(tert-butyldimethylsilyl)uridine (* ***8*** *)* (37). 2, 2′-*O-Cyclouridine* (**1**) (5.00 g, 22.1 mmol, 1 eq.) were dissolved in dimethylformamide (DMF, 55 ml). 15-crown-5 (21.9 ml, 110.5 mmol, 5 eq.), and sodium azide (7.19 g, 110.5 mmol, 5 eq.) was added and the mixture was refluxed at 120°C. After stirring for 16 h, the reaction mixture was allowed to cool to room temperature. Subsequently, imidazole (7.52 g, 110.5 mmol, 5 eq.) and *tert*-butyldimethylchlorosilane (9.34 ml, 66.3 mmol, 3 equiv.) was added, and the mixture was stirred at room temperature for 20 h. The reaction mixture was diluted with AcOEt and washed with a saturated NaHCO_3_ aqueous solution and brine. The organic layer was dried over Na_2_SO_4_, filtered, and concentrated *in vacuo*. The crude product was purified by silica gel column chromatography (hexane/AcOEt = 3/1) and compound **8** (4.50 g, 9.04 mmol, 41%) was obtained. ^1^H-NMR (400 MHz, CDCl_3_): δ 8.716 (s, 1H), 7.856 (d, *J* = 8.4 Hz, 1H), 6.091 (d, *J* = 4.8 Hz, 1H), 5.704 (d, *J* = 8.0 Hz, 1H), 4.367 (t, *J* = 4.8 Hz, 1H), 4.075–4.054 (m, 1H), 3.964 (dd, *J* = 12.0, 2.4 Hz, 1H), 3.747 (dd, *J* = 12.2, 1.8 Hz, 1H), 3.655 (t, *J* = 5.2 Hz, 1H), 0.937 (s, 9H), 0.921 (s, 9H), 0.169 (s, 3H), 0.132 (s, 3H), 0.116 (s, 3H), 0.109 (s, 3H); ^13^C-NMR (100 MHz, CDCl_3_): δ 162.949, 150.179, 139.487, 102.720, 86.535, 85.725, 72.143, 66.201, 61.948, 26.058, 25.839, 18.533, 18.219, −4.500, −5.368; HRMS (ESI-TOF) calcd. for C_21_H_39_N_5_O_5_Si_2_ [M + Na]^+^ 520.2382, found 520.2397.

*2′-Amino-2′-deoxy-3′,5′-di-(tert-butyldimethylsilyl)uridine (* ***9*** *)* (35). Compound **8** (4.40 g, 8.84 mmol) was then dissolved in methanol (88 ml). Pd: carbon powder (Pd: 10%; H_2_O: 55.64%; N. E. CHEMCAT) (0.84 g) was added and stirred at room temperature with H_2_ bubbling. After stirring for 4 h, the reaction mixture was filtered through Celite^®^ and concentrated *in vacuo*. Compound **9** (4.11 g, 8.71 mmol, 99% yield) was obtained. ^1^H-NMR (400 MHz, CDCl_3_): δ 7.818 (d, *J* = 8.4 Hz, 1H), 5.869 (d, *J* = 6.4 Hz, 1H), 5.672 (d, *J* = 8.8 Hz, 1H), 4.148 (q, *J* = 2.8 Hz, 1H), 4.061 (q, *J* = 2.5 Hz, 1H), 3.886 (dd, *J* = 11.6, 2.8 Hz, 1H), 3.733 (dd, *J* = 11.6, 2.0 Hz, 1H), 3.344 (dd, *J* = 5.2, 6.4 Hz, 1H), 0.927 (s, 9H), 0.921 (s, 9H), 0.120–0.108 (m, 12H); ^13^C-NMR (100 MHz, CDCl_3_): δ 163.150, 150.865, 140.203, 102.520, 89.597, 86.345, 73.135, 63.102, 59.811, 26.049, 25.896, 18.514, 18.238, −4.471, −5.387; HRMS (ESI-TOF) calcd. for C_21_H_41_N_3_O_5_Si_2_ [M + Na]^+^ 494.2477, found 494.2479.

*2′-Deoxy-2′-formamido-3′, 5′-di-(tert-butyldimethylsilyl)uridine (* ***10*** *)*. Compound **9** (4.10 g, 8.69 mmol, 1 equiv.) was dissolved in dichloromethane (80 ml). DIPEA (7.57 ml, 43.5 mmol, 5 equiv.), EDC·HCl (5.00 g, 26.1 mmol, 3 eq.), DMAP (0.106 g, 0.869 mmol, 0.1 eq.), and formic acid (0.984 ml, 26.1 mmol, 3 equiv.) was then added, and the mixture was stirred at room temperature. After stirring for 15 h, the reaction mixture was diluted with AcOEt and washed with a saturated NaHCO_3_ aqueous solution and brine. The organic layer was dried over Na_2_SO_4_, filtered, and concentrated *in vacuo*. The crude product was purified by silica gel column chromatography (hexane/AcOEt = 1/2) and compound **10** (3.71 g, 7.42 mmol, 85%) was obtained. ^1^H-NMR (400 MHz, CDCl_3_): δ 8.187 (s, 1H), 7.760 (d, *J*= 8.4 Hz, 1H), 6.102 (d, *J* = 8.8 Hz, 1H), 5.738 (dd, *J* = 8.4, 2.0 Hz, 1H), 4.604–4.561 (m, 1H), 4.289 (d, *J* = 5.2 Hz, 1H), 4.085 (m, 1H), 3.861 (dd, *J* = 11.8, 3.0 Hz, 1H), 3.758 (dd, *J* = 11.6, 2.4 Hz, 1H), 0.942 (s, 9H), 0.932 (s, 9H), 0.139 (s, 3H), 0.129 (s, 3H), 0.112 (s, 3H), 0.108 (s, 3H); ^13^C-NMR (100 MHz, CDCl_3_): δ 162.654, 161.175, 150.951, 139.745, 103.359, 87.537, 86.545, 73.126, 63.750, 54.747, 26.011, 25.915, 18.505, 18.257, −4.414, −5.387; HRMS (ESI-TOF) calcd. for C_22_H_41_N_3_O_6_Si_2_ [M + Na]^+^ 522.2426, found 522.2445.

*2′-Deoxy-2′-formamido-3′,5′-di-(tert-butyldimethylsilyl)cytidine (* ***11*** *)* (38). Compound **10** (3.70 g, 7.40 mmol and 1 equiv.) were dissolved in acetonitrile (70 ml). 2, 4, 6-triisopropylbenzenesulfonyl chloride (6.73 g, 22.2 mmol, 3 eq.) and DMAP (0.362 g, 2.96 mmol, 0.4 eq.) were added. triethylamine (3.10 ml, 22.2 mmol, 3 equiv.) was added dropwise at 0°C and the mixture was stirred at room temperature. After stirring for 3.5 h, a 28% ammonia solution (75 ml, 1110 mmol, 150 eq.) was then added, and the mixture was stirred for 4 h. The reaction mixture was diluted with AcOEt and washed with a saturated NaHCO_3_ aqueous solution and brine. The organic layer was dried over Na_2_SO_4_, filtered, and concentrated *in vacuo*. The crude product was purified using silica gel column chromatography (AcOEt/MeOH = 10/1), and compound **11** (3.20 g, 6.42 mmol, 87%) was obtained. ^1^H-NMR (400 MHz, CDCl_3_): δ 8.134 (s, 1H), 7.793 (d, *J* = 8.0 Hz, 1H), 6.475 (d, *J* = 8.0 Hz, 1H), 6.239 (d, *J* = 8.8 Hz, 1H), 6.077 (d, *J* = 8.8 Hz, 1H), 4.415–4.359 (m, 1H), 4.303 (d, *J* = 5.2 Hz, 1H), 4.106 (m, 1H), 3.827 (dd, *J* = 11.6, 3.2 Hz, 1H), 3.733 (dd, *J* = 11.4, 2.2 Hz, 1H), 0.932 (s, 9H), 0.909 (s, 9H), 0.116 (s, 3H), 0.109 (s, 6H), 0.083 (s, 3H); ^13^C-NMR (100 MHz, CDCl_3_): δ 162.845, 161.347, 148.014, 140.775, 97.360, 88.357, 86.898, 73.355, 63.703, 56.721, 26.039, 26.001, 18.447, 18.276, −4.519, −5.310; HRMS (ESI-TOF) calcd. for C_22_H_42_N_4_O_5_Si_2_ [M + Na]^+^ 521.2586, found 521.2602.

*4-N-acetyl-2′-deoxy-2′-formamidocytidine (* ***13*** *)* (39,40). Compound **11** (3.15 g, 6.32 mmol, 1 equiv.) were dissolved in pyridine (60 ml). Acetic anhydrid (2.98 ml, 31.6 mmol, 5 equiv.) was added, and the mixture was stirred at 80°C. After stirring for 2 h, the solvent was removed *in vacuo* and the solvent was co-evaporated with toluene. The crude product was dissolved in tetrahydrofuran (60 ml). Triethylamine trihydrofluoride (5.15 ml, 31.6 mmol, 5 equiv.) was added, and the mixture was stirred at room temperature for 22 h. The reaction mixture was then concentrated *in vacuo*. The crude product was purified by silica gel column chromatography (DCM/MeOH = 3/1), and compound **13** (1.28 g, 4.11 mmol, 65%) was obtained. ^1^H-NMR (400 MHz, DMSO-*d*_6_): δ 10.876 (s, 1H), 8.300 (d, *J* = 7.6 Hz, 1H), 8.148 (d, *J* = 9.2 Hz, 1H), 7.986 (s, 1H), 7.200 (d, *J* = 7.6 Hz, 1H), 6.038 (d, *J* = 8.4 Hz, 1H), 5.799 (d, *J* = 5.2 Hz, 1H), 5.188 (t, *J* = 5.4 Hz, 1H), 4.543–4.486 (m, 1H), 4.105 (td, *J* = 5.4, 1.6 Hz, 1H), 3.982–3.962 (m, 1H), 3.665–3.563 (m, 2H), 2.100 (s, 3H); ^13^C-NMR (100 MHz, DMSO-*d*_6_): δ 171.099, 162.381, 161.552, 154.990, 145.586, 95.963, 87.007, 86.950, 70.526, 61.485, 54.341, 24.394; HRMS (ESI-TOF) calcd. for C_12_H_16_N_4_O_6_ [M + Na]^+^ 335.0962, found 335.0962.

*4-N-acetyl-2′-deoxy-5′-O- (4, 4′-dimethoxytrityl)-2′-formamidocytidine (* ***14*** *)* (35). Compound **13** (1.25 g, 4.00 mmol, 1 eq.) were dissolved in pyridine (10 ml). DMTrCl (1.49 g, 4.40 mmol, 1.1 eq.) and DMAP (9.78 mg, 0.0801 mmol, 0.02 eq.) was then added, and the mixture was stirred at room temperature. After stirring for 16 h, the reaction mixture was diluted with AcOEt and washed with a saturated NaHCO_3_ aqueous solution and brine. The organic layer was dried over Na_2_SO_4_, filtered, and concentrated *in vacuo*. The crude product was purified by silica gel column chromatography (AcOEt/MeOH = 7/1), and compound **14** (2.34 g, 3.80 mmol, 95%) was obtained. ^1^H-NMR (400 MHz, CDCl_3_): δ 9.370 (s, 1H), 8.234 (s, 1H), 8.013 (d, *J* = 7.6 Hz, 1H), 7.911 (d, *J* = 8.0 Hz, 1H), 7.401 (d, *J* = 7.2 Hz, 1H), 7.309–7.178 (m, 7H), 6.825 (d, *J* = 8.4 Hz, 4H), 6.375 (d, *J* = 8.0 Hz, 1H), 4.912 (s, 1H), 4.663 (dd, *J* = 13.2, 8.0 Hz, 1H), 4.558 (s, 1H), 4.292 (s, 1H), 3.759 (s, 6H), 3.469–3.390 (m, 2H), 2.119 (s, 3H); ^13^C-NMR (100 MHz, CDCl_3_): δ 170.673, 163.507, 162.770, 158.813, 156.954, 144.711, 144.261, 135.456, 135.236, 130.216, 128.252, 127.275, 113.508, 97.834, 87.210*, 86.146, 72.015, 63.824, 58.076, 55.384, 24.966; HRMS (ESI-TOF) calcd. for C_33_H_34_N_4_O_8_ [M + Na]^+^ 637.2269, found 637.2258. *87.210 ppm peak of ^13^C-NMR overlapped with the two peaks.

*4-N-acetyl-2′-deoxy-5′-O-(4,4′-dimethoxytrityl)-2′-formamidocytidine-3′-O-(2-cyanoethyl N,N-diisopropylphosphoramidite) (* ***15*** *)* (36). Compound **14** (2.30 g, 3.74 mmol, 1 eq.) were co-evaporated with toluene and dissolved in dichloromethane (37 ml). DIPEA (3.26 ml, 18.7 mmol, 5 eq.) and 2-cyanoethyl-*N, N*-diisopropylchlorophosphoroamidite (0.920 ml, 4.12 mmol, 1.1 eq.) were added at 0°C and stirred at room temperature. After stirring for 18 h, the reaction mixture was diluted with AcOEt and washed with a saturated NaHCO_3_ aqueous solution and brine. The organic layer was dried over Na_2_SO_4_, filtered, and concentrated *in vacuo*. The crude product was purified by silica gel column chromatography (DCM/ACN/TEA = 33/66/1→20/79/1) to obtain compound **15** (2.32 g, 2.85 mmol, 76%). ^1^H-NMR (400 MHz, CDCl_3_): δ 8.702 (br, 1H), 8.234–8.181 (m, 1H), 8.003–7.920 (m, 1H), 7.434–7.412 (m, 2H), 7.331–7.218 (m, 8H), 6.872–6.845 (m, 4H), 6.780–6.577 (m, 1H), 6.344–6.305 (m, 1H), 4.786–4.530 (m, 2H), 4.456–4.306 (m, 1H), 3.900–3.824 (m, 1H), 3.801 (s, 6H), 3.770–3.726 (m, 1H), 3.643–3.576 (m, 2H), 3.514–3.406 (m, 2H), 2.652–2.621 (m, 1H), 2.475–2.434 (m, 1H), 2.206–2.203 (m, 3H), 1.193–1.117 (m, 12H); ^13^C-NMR (100 MHz, CDCl_3_): δ 170.370, 162.625, 161.938, 158.848, 156.092, 144.533, 144.113, 135.338, 135.195, 130.293, 128.319, 127.298, 117.761, 113.507, 97.360, 87.308, 87.012, 85.706, 73.860, 63.579, 58.457, 56.492, 55.415, 43.550, 25.114, 24.656, 20.508; ^31^P-NMR (160 MHz, CDCl_3_): δ 151.825, 150.997; HRMS (ESI-TOF) calcd. for C_42_H_51_N_6_O_9_P [M + Na]^+^ 837.3347, found 837.3353.

*3′, 5′-O-(1,1,3,3-Tetraisopropyldisiloxane-1,3-diyl)adenosine (* ***17*** *)* (41). Adenosine (**16**) (5.00 g, 18.7 mmol, 1 eq.) were dissolved in pyridine (90 ml). 1,3-Dichloro-1,1,3,3-tetraisopropyldisiloxane (6.58 ml, 20.6 mmol, 1.1 eq.) was added dropwise at 0°C, and the mixture was stirred at room temperature. After stirring for 12 h, the reaction mixture was diluted with AcOEt and washed with a saturated NaHCO_3_ aqueous solution and brine. The organic layer was dried over Na_2_SO_4_, filtered, and concentrated *in vacuo*. The crude product was purified by silica gel column chromatography (hexane/AcOEt = 1/3), and compound **17** (9.49 g, 18.6 mmol, 99%) was obtained. ^1^H-NMR (400 MHz, CDCl_3_): δ 8.287 (s, 1H), 7.964 (s, 1H), 5.974 (d, *J* = 0.8 Hz, 1H), 5.658 (br, 2H), 5.089 (dd, *J* = 8.0, 5.6 Hz, 1H), 4.572 (d, *J* = 5.2 Hz, 1H), 4.160–4.013 (m, 3H), 3.333 (s, 1H), 1.136–1.039 (m, 28H); ^13^C-NMR (100 MHz, CDCl_3_): δ 155.584, 153.180, 149.290, 139.614, 120.482, 89.854, 82.305, 75.273, 70.847, 61.851, 17.599, 17.494, 17.254, 17.120, 13.422, 13.202, 12.914, 12.752; HRMS (ESI-TOF) calcd. for C_22_H_39_N_5_O_5_Si_2_ [M + Na]^+^ 532.2382, found 532.2391.

*3′, 5′-O-(1,1,3,3-Tetraisopropyldisiloxane-1,3-diyl) (β-d-arabinofuranosyl)adenine (* ***19*** *)* (42). Pyridine (11.9 ml, 147 mmol, 8 equiv.) was added to a stirred suspension of chromium (VI) oxide (7.35 g, 73.8 mmol, 4 eq.) in dichloromethane (140 ml), at room temperature. After stirring the mixture for 30 min, acetic anhydride (6.92 ml, 73.8 mmol, 4 equiv.) was added. After 30 min, a solution of **17** (9.40 g, 18.4 mmol, 1 equiv.) in dichloromethane (40 ml) was then added dropwise to the solution. After stirring at room temperature for 20 h, the mixture was pipetted into a silica gel column covered with ethyl acetate (1 l). The column was eluted using ethylacetate. The eluate was evaporated and then evaporated with toluene. The residue was dissolved in ethanol (55 ml), and a solution of sodium borohydride (0.977 g, 25.8 mmol, 1.4 eq.) in water (5.5 ml) were added dropwise at 0°C. After stirring for 11 h at 0°C, the reaction mixture was diluted with AcOEt and washed with a saturated NaHCO_3_ aqueous solution and brine. The organic layer was dried over Na_2_SO_4_, filtered, and concentrated *in vacuo*. The crude product was purified by silica gel column chromatography (AcOEt/MeOH = 20/1), and compound **19** (2.713 g, 5.2 mmol, 29%) was obtained. ^1^H-NMR (400 MHz, CDCl_3_): δ 8.188 (s, 1H), 8.102 (s, 1H), 6.181 (d, *J* = 6.0 Hz, 1H), 6.042 (br, 2H), 4.680–4.596 (m, 2H), 4.028 (d, *J* = 3.6 Hz, 2H), 3.860–3.824 (m, 1H), 1.121–1.038 (m, 28H); ^13^C-NMR (100 MHz, CDCl_3_): δ 155.710, 152.763, 149.454, 140.546, 119.935, 84.218, 81.318, *, 74.413, 61.480, 17.627, 17.475, 17.208, 17.112, 13.660, 13.192, 13.021, 12.582; HRMS (ESI-TOF) calcd. for C_22_H_39_N_5_O_5_Si_2_ [M + Na]^+^ 532.2382, found 532.2391. *1 13C-NMR peak overlaps with the solvent peak.

*3′, 5′-O-(1,1,3,3-Tetraisopropyldisiloxane-1, 3-diyl)-2′-O-(trifluoromethanesulfonyl) (β-d-arabinofuranosyl)adenine (* ***20*** *)* (43). Compound **19** (2.70 g, 5.30 mmol, 1 equiv.) were dissolved in dichloromethane (50 ml). DMAP (1.94 g, 15.9 mmol, 3 eq.) was added, and the mixture was stirred at 0°C. *N-Phenylbis* (trifluoromethanesulfonimide) (2.84 g, 7.95 mmol, 1.5 eq.) was added, and the mixture was stirred at 0°C for 1 h. The reaction mixture was diluted with AcOEt and washed with a saturated NaHCO_3_ aqueous solution and brine. The organic layer was dried over Na_2_SO_4_, filtered, and concentrated *in vacuo*. The crude product was purified by silica gel column chromatography (hexane/AcOEt = 1/2), and compound **20** (3.30 g, 5.14 mmol, 97%) was obtained. ^1^H-NMR (400 MHz, CDCl_3_): δ 8.321 (s, 1H), 7.925 (s, 1H), 6.392 (d, *J* = 6.0 Hz, 1H), 5.769 (br, 2H), 5.474 (t, *J* = 6.4 Hz, 1H), 5.384 (t, *J* = 7.0 Hz, 1H), 4.216 (dd, *J* = 12.6, 6.2 Hz, 1H), 4.078 (dd, *J* = 12.6, 3.4 Hz, 1H), 3.974–3.931 (m, 1H), 1.198–1.042 (m, 28H); ^13^C-NMR (100 MHz, CDCl_3_): δ 155.682, 153.402, 149.759, 139.707, 119.945, 116.702, 88.586, 81.309, 80.937, 74.423, 62.215, 17.570, 17.456, 16.988, 16.874, 13.354, 13.211, 13.116, 12.715; ^19^F-NMR (380 MHz, CDCl_3_): −74.288; HRMS (ESI-TOF) calcd. for C_23_H_38_F_3_N_5_O_7_SSi_2_ [M + Na]^+^ 664.1875, found 664.1892.

*6-N-acetyl-2′-azide-2′-deoxy-3′, 5′-O-(1, 1, 3, 3-tetraisopropyldisiloxane-1,3-diyl)adenosine (* ***22*** *)* (44). Compound **20** (3.10 g, 4.83 mmol, 1 equiv.) was dissolved in dimethylformamide (DMF; 48 ml). Sodium azide (0.942 g, 14.5 mmol, 3 equiv.) was added, and the mixture was stirred at 60°C. After stirring for 16 h, the reaction mixture was diluted with AcOEt and washed with a saturated NaHCO_3_ aqueous solution and brine. The organic layer was dried over Na_2_SO_4_, filtered, and concentrated *in vacuo*. The residue was then coevaporated with pyridine and dissolved in pyridine (48 ml). Acetyl chloride (0.377 ml, 5.31 mmol, 1.1 eq.) was added dropwise at 0°C, and the mixture was stirred at room temperature for 14 h. The reaction mixture was diluted with AcOEt and washed with a saturated NaHCO_3_ aqueous solution and brine. The organic layer was dried over Na_2_SO_4_, filtered, and concentrated *in vacuo*. The crude product was purified by silica gel column chromatography (hexane/AcOEt = 1/2), and compound **22** (1.77 g, 3.06 mmol, 63%) was obtained. ^1^H-NMR (400 MHz, CDCl_3_): δ 8.707 (s, 1H), 8.653 (s, 1H), 8.189 (s, 1H), 5.810 (s, 1H), 5.151 (dd, *J* = 9.2, 5.6 Hz, 1H), 4.604 (d, *J* = 5.2 Hz, 1H), 4.185 (dd, *J* = 13.6, 2.0 Hz, 1H), 4.148–4.114 (m, 1H), 4.043 (dd, *J* = 13.6, 2.8 Hz, 1H), 2.614 (s, 3H), 1.097–1.062 (m, 28H); ^13^C-NMR (100 MHz, CDCl_3_): δ 170.474, 152.678, 150.341, 149.435, 141.557, 122.511, 87.718, 81.995, 71.075, 65.429, 60.078, 25.839, 17.541, 17.398, 17.150, 16.969, 13.536, 13.068, 12.868, 12.830; HRMS (ESI-TOF) calcd. for C_24_H_40_N_8_O_5_Si_2_ [M + Na]^+^ 599.2552, found 599.2557.

*6-N-Acetyl-2′-amino-2′-deoxy-3′, 5′-O-(1,1,3,3-tetraisopropyldisiloxane-1, 3-diyl)adenosine (* ***23*** *)* (35). Compound **22** (1.75 g, 3.03 mmol) was then dissolved in methanol (30 ml). Pd: carbon powder (Pd: 10%; H_2_O: 55.64%; N. E. CHEMCAT) (0.287 g) was added and stirred at room temperature with H_2_ bubbling. After stirring for 6 h, the reaction mixture was filtered through Celite^®^ and concentrated *in vacuo*. The crude product was purified using silica gel column chromatography (DCM/MeOH = 15/1) and compound **23** (1.14 g, 2.07 mmol, 68%). ^1^H-NMR (400 MHz, CDCl_3_): δ 8.734 (br, 1H), 8.656 (s, 1H), 8.267 (s, 1H), 5.926 (d, *J* = 2.4 Hz, 1H), 4.708 (t, *J* = 7.0 Hz, 1H), 4.231 (quin, *J* = 3.6 Hz, 1H), 4.155 (dd, *J* = 13.2, 4.0 Hz, 1H), 4.053 (dd, *J* = 12.8, 3.2 Hz, 1H), 3.893 (dd, *J* = 6.2, 2.6 Hz, 1H), 2.604 (s, 3H), 1.097–1.001 (m, 28H); ^13^C-NMR (100 MHz, CDCl_3_): δ 170.424, 152.442, 150.651, 149.271, 141.396, 122.484, 90.553, 82.975, 69.908, 61.506, 58.411, 25.829, 17.618, 17.465, 17.293, 17.130, 13.528, 13.211, 13.039, 12.752; HRMS (ESI-TOF) calcd. for C_24_H_42_N_6_O_5_Si_2_ [M + Na]^+^ 573.2647, found 573.2663.

*6-N-Acetyl-2′-deoxy-2′-formamido-3′,5′-O-(1,1,3,3-tetraisopropyldisiloxane-1, 3-diyl)adenosine (* ***24*** *)*. Compound **23** (1.05 g, 1.91 mmol, 1 equiv.) were dissolved in dichloromethane (19 ml). DIPEA (0.996 ml, 5.72 mmol, 3 equiv.), EDC·HCl (0.548 g, 2.86 mmol, 1.5 eq.), DMAP (23.3 mg, 0.191 mmol, 0.1 eq.), and formic acid (0.108 ml, 2.86 mmol, 1.5 eq.) was then added, and the mixture was stirred at room temperature. After stirring for 16 h, the reaction mixture was diluted with AcOEt and washed with a saturated NaHCO_3_ aqueous solution and brine. The organic layer was dried over Na_2_SO_4_, filtered, and concentrated *in vacuo*. The crude product was purified using silica gel column chromatography (AcOEt/MeOH = 15/1) and compound **24** (1.00 g, 1.72 mmol, 91%). ^1^H-NMR (400 MHz, CDCl_3_): δ 8.707 (br, 1H), 8.608 (s, 1H), 8.329 (s, 1H), 8.119 (s, 1H), 6.606 (d, *J* = 4.0 Hz, 1H), 6.032 (d, *J* = 3.6 Hz, 1H), 5.391 (t, *J* = 7.4 Hz, 1H), 4.795 (quin, *J* = 4.0 Hz, 1H), 4.084–3.998 (m, 3H), 2.605 (s, 3H), 1.184–1.053 (m, 28H); ^13^C-NMR (100 MHz, CDCl_3_): δ 170.417, 161.929, 152.411, 150.865, 149.483, 142.692, 122.482, 88.510, 83.798, 70.074, 62.425, 55.357, 25.829, 17.570, 17.456, 17.265, 17.122, 13.326, 13.278, 12.935, 12.715; HRMS (ESI-TOF) calcd. for C_25_H_42_N_6_O_6_Si_2_ [M + Na]^+^ 601.2597, found 601.2611.

*6-N-Acetyl-2′-deoxy-2′-formamidoadenosine (* ***25*** *)* (40). Compound **24** (950 mg, 1.64 mmol, and 1 equiv.) was dissolved in tetrahydrofuran (THF, 16 ml). Triethylamine trihydrofluoride (1.34 ml, 8.21 mmol, 5 equiv.) was then added, and the mixture was stirred at room temperature. After stirring for 16 h, the reaction mixture was concentrated under vacuum. The crude product was purified using silica gel column chromatography (AcOEt/MeOH = 7/3), and **25** (542 mg, 1.61 mmol, 98%) was obtained. ^1^H-NMR (400 MHz, DMSO-*d*_6_): δ 10.705 (s, 1H), 8.666 (s, 1H), 8.642 (s, 1H), 8.322 (d, *J* = 8.8 Hz, 1H), 7.941 (m, 1H), 6.051 (d, *J* = 8.4 Hz, 1H), 5.917 (d, *J* = 4.8 Hz, 1H), 5.252 (dd, *J* = 6.4, 5.2 Hz, 1H), 5.182–5.125 (m, 1H), 4.277–4.216 (m, 1H), 4.084–4.058 (m, 1H), 3.731–3.578 (m, 2H), 2.255 (s, 3H); ^13^C-NMR (100 MHz, DMSO-*d*_6_): δ 168.838, 161.561, 151.909, 151.690, 149.592, 142.744, 123.497, 87.522, 85.872, 70.517, 61.666, 53.502, 24.346; HRMS (ESI-TOF) calcd. for C_13_H_16_N_6_O_5_ [M + Na]^+^ 359.1074, found 359.1081.

*6-N-Acetyl-5′-O-(4, 4′-dimethoxytrityl)-2′-formamidoadenosine (* ***26*** *)* (35). Compound **25** (530 mg, 1.58 mmol, 1 equiv.) were dissolved in pyridine (6 ml). DMTrCl (587 mg, 1.73 mmol, 1.1 eq.) and DMAP (3.85 mg, 0.0315 mmol, 0.02 eq.) was then added, and the mixture was stirred at room temperature. After stirring for 16 h, the reaction mixture was diluted with AcOEt and washed with a saturated NaHCO_3_ aqueous solution and brine. The organic layer was dried over Na_2_SO_4_, filtered, and concentrated *in vacuo*. The crude product was purified using silica gel column chromatography (AcOEt/MeOH = 7/1), and compound **26** (971 mg, 1.52 mmol, 97%) was obtained. ^1^H-NMR (400 MHz, CDCl_3_): δ 9.042 (s, 1H), 8.553 (s, 1H), 8.249 (s, 1H), 8.119 (s, 1H), 7.402–7.380 (m, 2H), 7.306–7.161 (m, 8H), 6.793–6.762 (m, 4H), 6.218 (d, *J* = 8.0 Hz, 1H), 5.260 (dd, *J* = 13.8, 7.8 Hz, 1H), 4.649–4.637 (m, 1H), 4.303–4.288 (m, 2H), 3.748 (s, 6H), 3.479–3.393 (m, 2H), 2.535 (s, 3H); ^13^C-NMR (100 MHz, CDCl_3_): δ 170.742, 162.034, 158.762, 152.468, 151.695, 149.206, 144.390, 141.853, 135.567, 130.207, 128.271, 128.119, 127.203, 121.938, 113.402, 87.003, 86.402, 85.896, 71.857, 63.798, 55.386, 54.919, 25.734; HRMS (ESI-TOF) calcd. for C_34_H_34_N_6_O_7_ [M + Na]^+^ 661.2381, found 661.2386.

*6-N-Acetyl-2′-deoxy-5′-O-(4,4′-dimethoxytrityl)-2′-formamidoadenosine-3′-O-(2-cyanoethyl N,N-diisopropylphosphoramidite) (* ***27*** *)* (36). Compound **26** (950 mg, 1.49 mmol, 1 eq.) were co-evaporated with toluene and dissolved in dichloromethane (15 ml). DIPEA (1.30 ml, 7.44 mmol, 5 equiv.), and 2-cyanoethyl-*N,N-diisopropylchlorophosphoramidite* (0.366 ml, 1.64 mmol, 1.1 eq.) were added at 0°C and stirred at room temperature. After stirring for 20 h, the reaction mixture was diluted with AcOEt and washed with a saturated NaHCO_3_ aqueous solution and brine. The organic layer was dried over Na_2_SO_4_, filtered, and concentrated *in vacuo*. The crude product was purified by silica gel column chromatography (DCM/ACN/TEA = 33/66/1→25/74/1) to obtain compound **27** (784 mg, 0.935 mmol, 63%). ^1^H-NMR (400 MHz, CDCl_3_): δ 8.633, 8.621 (2s, 1H), 8.588 (br, 1H), 8.265, 8.247 (2s, 1H), 8.198, 8.145 (2s, 1H), 7.430–7.409 (m, 2H), 7.322–7.196 (m, 7H), 6.820–6.792 (m, 4H), 6.645, 6.501 (2d, *J* = 8.6 Hz, 1H), 6.182, 6.138 (2d, *J* = 7.8 Hz, 1H), 5.519–5.388 (m, 1H), 4.743–4.627 (m, 1H), 4.494–4.347 (m, 1H), 3.925–3.719 (m, 8H), 5.680–3.596 (m, 2H), 3.528–3.348 (m, 2H), 2.660–2.440 (m, 5H), 1.251–1.147 (m, 12H); ^13^C-NMR (100 MHz, CDCl_3_): δ 170.684, 161.748, 158.782, 152.620, 151.733, 149.311, 144.380, 141.586, 135.520, 130.226, 128.309, 128.119, 127.193, 122.005, 118.047, 113.393, 87.031, 86.440, 85.305, 73.078, 63.483, 58.314, 55.386, 53.679, 43.550, 25.801, 24.685, 20.603; ^31^P-NMR (160 MHz, CDCl_3_): δ 152.333, 149.981; HRMS (ESI-TOF) calcd. for C_43_H_51_N_8_O_8_P [M + Na]^+^ 861.3460, found 861.3466.

*2-N-Isobutyrylguanosine (* ***29*** *)* (45). Guanosine (**28**) (5.0 g, 17.7 mmol, 1 eq.) was coevaporated with pyridine and dissolved in pyridine (150 ml). Trimethylsilyl chloride (16.7 ml, 132 mmol, 7.5 eq.) was added dropwise at 0°C, and the mixture was stirred at room temperature for 3 h. Isobutyryl chloride (5.59 ml, 53.0 mmol, 3 equiv.) was then added dropwise to the solution at 0°C. After stirring at room temperature for 14 h, H_2_O (15 ml) was added at 0°C and the mixture was stirred at room temperature for 30 min. A solution of 28% ammonia solution (23.9 ml, 353 mmol, 20 equiv.) was added and the mixture was stirred for 30 min at room temperature. The reaction mixture was diluted with water and washed with dichloromethane. The aqueous layer was concentrated *in vacuo* and recrystallized from hot water to obtain compound **29** (5.78 g, 16.4 mmol, 93%). ^1^H-NMR (400 MHz, DMSO-*d*_6_): δ 12.078 (br, 1H), 11.737 (br, 1H), 8.265 (s, 1H), 5.793 (d, *J* = 6.0 Hz, 1H), 5.531 (d, *J* = 5.6 Hz, 1H), 5.241 (d, *J* = 3.2 Hz, 1H), 5.099 (br, 1H), 4.428 (q, *J* = 5.2 Hz, 1H), 4.136 (m, 1H), 3.899 (q, *J* = 4.0 Hz, 1H), 3.573 (m, 2H), 2.784 (sep, *J* = 3.0 Hz, 1H), 1.107 (d, *J* = 6.8 Hz, 6H); ^13^C-NMR (100 MHz, DMSO-*d*_6_): δ 180.178, 154.895, 148.905, 148.152, 137.670, 120.140, 86.540, 85.338, 74.036, 70.250, 61.151, 34.761, 18.919; HRMS (ESI-TOF) calcd. for C_14_H_19_N_5_O_6_ [M + Na]^+^ 376.1228, found 376.1233.

*2-N-Isobutyryl-3′, 5′-O-(1,1,3,3-tetraisopropyldisiloxane-1, 3-diyl)guanosine (* ***30*** *)* (41). Compound **29** (5.00 g, 14.2 mmol, 1 equiv.) was coevaporated with pyridine and dissolved in pyridine (140 ml). 1, 3-dichloro-1, 1, 3, 3-tetraisopropyldisiloxane (4.98 ml, 15.6 mmol, 1.1 eq.) was added dropwise at 0°C, and the mixture was stirred at room temperature. After stirring for 12 h, the reaction mixture was diluted with dichloromethane and washed with a saturated NaHCO_3_ aqueous solution and brine. The organic layer was dried over Na_2_SO_4_, filtered, and concentrated *in vacuo*. The crude product was purified by silica gel column chromatography (DCM/MeOH = 100/0→98/2→96/4→94/6) to obtain compound **30** (8.10 g, 13.6 mmol, 96%). ^1^H-NMR (400 MHz, CDCl_3_): δ 11.990 (br, 1H), 8.372 (br, 1H), 7.889 (s, 1H), 5.888 (d, *J* = 1.2 Hz, 1H), 4.575 (dd, *J* = 8.2, 5.4 Hz, 1H), 4.296 (d, *J* = 5.2 Hz, 1H), 4.169–4.036 (m, 3H), 3.129 (br, 1H), 2.615 (sep, *J* = 3.0 Hz, 1H), 1.282 (dd, *J* = 7.2, 1.2 Hz, 6H), 1.106–1.016 (m, 28H); ^13^C-NMR (100 MHz, CDCl_3_): δ 178.702, 155.632, 147.786, 147.527, 136.730, 121.843, 88.867, 82.008, 75.483, 70.013, 61.027, 36.683, 19.161, 17.570, 17.408, 17.226, 17.091, 13.489, 13.116, 13.039, 12.675; HRMS (ESI-TOF) calcd. for C_26_H_45_N_5_O_7_Si_2_ [M + Na]^+^ 618.2750, found 618.2763.

*2-N-Isobutyryl-3′,5′-O-(1,1,3,3-tetraisopropyldisiloxane-1,3-diyl)-2′-O-(trifluoromethanesulfonyl)guanosine (* ***31*** *)* (44) and *1-[2-isobutyrylamino-9-(3′,5′-O-(1,1,3,3-tetraisopropyldisiloxane-1,3-diyl)-2′-O-(trifluoromethanesulfonyl)-9H-purin-6-yl]-4- (dimethylamino) pyridinium trifluoromethanesulfonate****(31b)***. Compound **30** (4.90 g, 8.22 mmol, 1 equiv.) was coevaporated with pyridine and dissolved in dichloromethane (82 ml). DMAP (3.01 g, 24.7 mmol, 3 eq.) Trifluoromethanesulfonyl chloride (1.30 ml, 12.3 mmol, 1.5 eq.) was added dropwise at 0°C and the mixture was stirred at 0°C. After stirring for 30 min, the reaction mixture was diluted with dichloromethane and washed with a saturated NaHCO_3_ aqueous solution and brine. The organic layer was dried over Na_2_SO_4_, filtered and concentrated *in vacuo*. The crude product was purified using silica gel column chromatography (hexane/AcOEt = 1/2). Compounds **31** (1.67 g, 2.29 mmol, 28%) and **31b** (3.53 g, 3.60 mmol, 44%) were obtained. The reaction mechanism of obtaining compound **31b** is shown in Supplementary Scheme S1. Compound **31**; ^1^H-NMR (400 MHz, CDCl_3_): δ 12.116 (br, 1H), 8.795 (s, 1H), 7.958 (s, 1H), 6.090 (s, 1H), 5.333 (d, *J* = 4.4 Hz, 1H), 4.700 (dd, *J* = 9.6, 4.4 Hz, 1H), 4.272 (d, *J* = 14 Hz, 1H), 4.116 (d, *J* = 9.6 Hz, 1H), 4.038 (dd, *J* = 13.8, 2.6 Hz, 1H), 2.677 (sep, *J* = 3.1 Hz, 1H), 1.258 (d, *J* = 6.8 Hz, 6H), 1.098–0.985 (m, 28H); ^13^C-NMR (100 MHz, CDCl_3_): δ 178.772, 155.463, 148.157, 147.203, 136.092, 121.986, 116.969, 88.290, 86.507, 81.681, 67.518, 59.296, 36.569, 19.058, 17.503, 17.351, 16.836, 16.807, 13.421, 13.040, 12.973, 12.887; ^19^F-NMR (380 MHz, CDCl_3_): -74.535; HRMS (ESI-TOF) calcd. for C_27_H_44_F_3_N_5_O_9_SSi_2_ [M + Na]^+^ 750.2243, found 750.2258. Compound **31b**; ^1^H-NMR (400 MHz, CDCl_3_): δ 9.863 (d, *J* = 8.4 Hz, 2H), 9.763 (s, 1H), 8.233 (s, 1H), 7.042 (d, *J* = 8.4 Hz, 2H), 6.202 (s, 1H), 5.636 (d, *J* = 5.6 Hz, 1H), 5.220 (dd, *J* = 9.0, 5.4 Hz, 1H), 4.349 (dd, *J* = 12.8, 4.0 Hz, 1H), 4.149–4.111 (m, 1H), 4.068 (dd, *J* = 13.0, 2.6 Hz, 1H), 3.420 (s, 6H), 2.845 (sep, *J* = 6.8 Hz, 1H), 1.240 (d, *J* = 6.4 Hz, 6H), 1.100–1.006 (m, 28H); ^13^C-NMR (100 MHz, CDCl_3_): δ 175.348, 157.847, 154.757, 153.050, 145.286, 143.255, 139.039, 122.406, 119.688, 117.007, 108.271, 88.195, 87.556, 82.634, 69.444, 60.984, 41.271, 36.225, 19.229, 17.532, 17.408, 17.188, 17.036, 13.040, 13.021, 12.925, 12.668; ^19^F-NMR (380 MHz, CDCl_3_): -74.659, -78.242; HRMS (ESI-TOF) calcd. for C_34_H_53_F_3_N_7_O_8_SSi_2_^+^ [M]^+^ 832.3161, found 832.3171.

*2-N-Isobutyryl-3′,5′-O-(1,1,3,3-tetraisopropyldisiloxane-1,3-diyl) (β-d-arabinofuranosyl)guanine (* ***32*** *)* (44). Compound **31** (7.30 g, 10.0 mmol, 1 equiv.) were dissolved in dimethylformamide (DMF, 100 ml). Potassium trifluoroacetate (7.62 g, 50.1 mmol, 5 equiv.), and DIPEA (2.62 ml, 15.0 mmol, 1.5 eq.) was added, and the mixture was stirred at 80°C. After stirring for 16 h, the reaction mixture was diluted with dichloromethane and washed with a saturated NaHCO_3_ aqueous solution and brine. The organic layer was dried over Na_2_SO_4_, filtered, and concentrated *in vacuo*. The crude product was purified by silica gel column chromatography (hexane/AcOEt = 1/1→1/2), and compound **32** (3.18 g, 5.34 mmol, 53%) was obtained. ^1^H-NMR (400 MHz, CDCl_3_): δ 11.724 (br, 1H), 8.796 (s, 1H), 7.879 (s, 1H), 6.074 (d, *J* = 4.0 Hz, 1H), 5.844 (br 1H), 4.754–4.748 (m, 1H), 4.486 (dd, *J* = 5.2, 3.2 Hz, 1H), 4.111–4.060 (m, 1H), 4.000–3.943 (m, 1H), 3.878–3.836 (m, 1H), 2.636 (sep, *J* = 7.5 Hz, 1H), 1.259 (dd, *J* = 7.0, 3.0 Hz, 6H), 1.111–1.024 (m, 28H); ^13^C-NMR (100 MHz, CDCl_3_): δ 178.715, 154.738, 147.546, 147.270, 139.812, 119.268, 84.332, 83.588, 79.087, 76.950, 63.569, 36.597, 19.249, 17.703, 17.551, 17.265, 17.141, 13.688, 13.488, 13.221, 12.601; HRMS (ESI-TOF) calcd. for C_26_H_45_N_5_O_7_Si_2_ [M + Na]^+^ 618.2750, found 618.2767.

*2-N-isobutyryl-3′,5′-O-(1,1,3,3-tetraisopropyldisiloxane-1,3-diyl)-2′-O-(trifluoromethanesulfonyl) (β-d-arabinofuranosyl)guanine (* ***33*** *)* (44) and *1-[2-isobutyrylamino-9- (3′,5′-O-(1,1,3,3-tetraisopropyldisiloxane-1,3-diyl)-2′-O- (trifluoromethanesulfonyl) (β-d-arabinofuranosyl)-9H-purin-6-yl]-4-(dimethylamino) pyridinium trifluoromethanesulfonate(33b)*. Compound **32** (3.15 g, 5.29 mmol, 1 equiv.) was coevaporated with pyridine and dissolved in dichloromethane (53 ml). DMAP (1.94 g, 15.9 mmol, 3 eq.) was added and trifluoromethanesulfonyl chloride (0.688 ml, 6.34 mmol, 1.2 eq.) was then added dropwise to the solution at 0°C. After stirring for 4 h at 0°C, the reaction mixture was diluted with dichloromethane and washed with a saturated NaHCO_3_ aqueous solution and brine. The organic layer was dried over Na_2_SO_4_, filtered, and concentrated *in vacuo*. The crude product was purified using silica gel column chromatography (DCM/MeOH = 100/0→99/1→98/2→96/4). Compounds **33** (0.767 g, 1.05 mmol, 20%) and **33b** (1.85 g, 1.88 mmol, 36%) were obtained. The mechanism of obtaining compound **33b** is shown in Supplementary Scheme S1. Compound **33**; ^1^H-NMR (400 MHz, CDCl_3_): δ 12.000 (br, 1H), 8.244 (s, 1H), 7.990 (s, 1H), 6.233 (d, *J* = 5.6 Hz, 1H), 5, 418 (t, *J* = 6.8 Hz, 1H), 4.826 (t, *J* = 8.0 Hz, 1H), 4.120–4.108 (m, 2H), 3.940–3.904 (m, 1H), 2.634 (sep, *J* = 6.9 Hz, 1H), 1.276 (d, *J* = 6.8 Hz, 6H), 1.101–1.028 (m, 28H); ^13^C-NMR (100 MHz, CDCl_3_): δ 178.261, 155.498, 147.997, 147.786, 136.874, 125.426, 121.459, 87.363, 80.983, 80.149, 71.738, 60.950, 36.769, 19.084, 17.436, 17.235, 16.938, 16.727, 13.365, 13.192, 13.096, 12.732; ^19^F-NMR (380 MHz, CDCl_3_): −74.207; HRMS (ESI-TOF) calcd. for C_27_H_44_F_3_N_5_O_9_SSi_2_ [M + Na]^+^ 750.2243, found 750.2269. Compound **33b**; ^1^H-NMR (400 MHz, CDCl_3_): δ 10.059 (s, 1H), 9.997 (d, *J* = 8.4 Hz, 2H), 8.126 (s, 1H), 7.053 (d, *J* = 8.4 Hz, 2H), 6.475 (d, *J* = 6.4 Hz, 1H), 5, 478 (t, *J* = 6.6 Hz, 1H), 5.439–5.424 (m, 1H), 4.508–4.462 (m, 1H), 4.052 (dd, *J* = 12.4, 2.4 Hz, 1H), 3.972–3.931 (m, 1H), 3.416 (s, 6H), 2.887 (sep, *J* = 6.8 Hz, 1H), 1.252 (d, *J* = 7.2 Hz, 6H), 1.163–0.988 (m, 28H); ^13^C-NMR (100 MHz, CDCl_3_): δ 175.825, 157.876, 155.796, 153.221, 145.105, 143.827, 139.468, 122.530, 119.258, 116.693, 108.290, 89.683, 81.843, 81.242, 74.823, 62.186, 41.232, 36.292, 19.296, 17.503, 17.436, 17.141, 16.836, 13.011, 12.954, 12.878, 12.839; ^19^F-NMR (380 MHz, CDCl_3_): -73.995, -78.180; HRMS (ESI-TOF) calcd. for C_34_H_53_F_3_N_7_O_8_SSi_2_^+^ [M]^+^ 832.3161, found 832.3186.

*2′-Azide-2′-deoxy-2-N-isobutyryl-3′,5′-O-(1,1,3,3-tetraisopropyldisiloxane-1,3-diyl)guanosine (* ***34*** *)*. Compound **33** (760 mg, 1.04 mmol, 1 equiv.) were dissolved in dimethylformamide (DMF, 10 ml). Sodium azide (102 mg, 1.57 mmol, 1.5 eq.) was then added, and the mixture was stirred at room temperature. After stirring for 17 h, the reaction mixture was diluted with AcOEt and washed with a saturated NaHCO_3_ aqueous solution and brine. The organic layer was dried over Na_2_SO_4_, filtered, and concentrated *in vacuo*. The crude product was purified using silica gel column chromatography (hexane/AcOEt = 1/2), and compound **34** (614 mg, 0.989 mmol, 95%) was obtained. ^1^H-NMR (400 MHz, CDCl_3_): δ 12.023 (br, 1H), 8.391 (s, 1H), 7.942 (s, 1H), 5.653 (s, 1H), 4.772 (dd, *J* = 9.0, 5.4 Hz, 1H), 4.224–4.186 (m, 2H), 4.128–4.094 (m, 1H), 4.034 (dd, *J* = 13.6, 2.8 Hz, 1H), 2.650 (sep, *J* = 7.0 Hz, 1H), 1.292 (dd, *J* = 7.0, 1.4 Hz, 6H), 1.102–1.017 (m, 28H); ^13^C-NMR (100 MHz, CDCl_3_): δ 178.333, 155.463, 147.737, 147.098, 136.397, 122.129, 86.841, 81.948, 70.627, 66.182, 59.878, 36.817, 19.144, 17.560, 17.398, 17.131, 17.026, 13.612, 13.097, 13.040, 12.792; HRMS (ESI-TOF) calcd. for C_26_H_44_N_8_O_6_Si_2_ [M + Na]^+^ 643.2815 found 643.2813.

*2′-Amino-2′-deoxy-2-N-isobutyryl-3′,5′-O-(1,1,3,3-tetraisopropyldisiloxane-1,3-diyl)guanosine (* ***35*** *)* (35). Compound **34** (1.40 g, 2.25 mmol) was then dissolved in methanol (22 ml). Pd: carbon powder (Pd: 10%; H_2_O: 55.64%; N. E. CHEMCAT) (0.213 g) was added and stirred at room temperature with H_2_ bubbling. After stirring for 4 h, the reaction mixture was filtered through Celite^®^ and concentrated *in vacuo*. The crude product was purified by silica gel column chromatography (AcOEt/MeOH = 20/1→10:1) to yield compound **35** (0.708 g, 1.19 mmol, 53%). ^1^H-NMR (400 MHz, CDCl_3_): δ 7.930 (s, 1H), 5.721 (d, *J* = 2.4 Hz, 1H), 4.517 (t, *J* = 6.8 Hz, 1H), 4.147–4.011 (m, 3H), 3.661 (dd, *J* = 6.4, 2.8 Hz, 1H), 2.647 (sep, *J* = 7.1 Hz, 1H), 1.269 (dd, *J* = 7.0, 1.0 Hz, 6H), 1.109–0.984 (m, 28H); ^13^C-NMR (100 MHz, CDCl_3_): δ 178.534, 155.634, 147.680, 147.604, 136.674, 121.824, 89.559, 82.892, 69.940, 61.480, 58.829, 36.702, 19.134, 17.627, 17.465, 17.322, 17.141, 13.545, 13.211, 13.126, 12.734; HRMS (ESI-TOF) calcd. for C_26_H_46_N_6_O_6_Si_2_ [M + Na]^+^ 617.2910, found 617.2923.

*2′-Deoxy-2′-formamido-2-N-isobutyryl-3′,5′-O-(1,1,3,3-tetraisopropyldisiloxane-1,3-diyl)guanosine (* ***36*** *)*. Compound **35** (700 mg, 1.18 mmol, 1 equiv.) was dissolved in dichloromethane (11 ml). DIPEA (0.615 ml, 3.53 mmol, 3 equiv.), EDC·HCl (338 mg, 1.77 mmol, 1.5 eq.), DMAP (14.4 mg, 0.118 mmol, 0.1 eq.), formic acid (0.0666 ml, 1.77 mmol, 1.5 eq.) was then added, and the mixture was stirred at room temperature. After stirring for 12 h, the reaction mixture was diluted with AcOEt and washed with a saturated NaHCO_3_ aqueous solution and brine. The organic layer was dried over Na_2_SO_4_, filtered, and concentrated *in vacuo*. The crude product was purified by silica gel column chromatography (AcOEt/MeOH = 30/1→10/1) and compound **36** (702 mg, 1.13 mmol, 96%) was obtained. ^1^H-NMR (400 MHz, CDCl_3_): δ 12.190 (br, 1H), 10.093 (s, 1H), 8.476 (s, 1H), 7.962 (s, 1H), 7.452 (m, 1H), 5.981 (d, *J* = 2.0 Hz, 1H), 4.738–4.685 (m, 2H), 4.069–4.016 (m, 3H), 2.647 (sep, *J* = 7.0 Hz, 1H), 1.215 (dd, *J* = 7.0, 1.4 Hz, 6H), 1.081–0.959 (m, 28H); ^13^C-NMR (100 MHz, CDCl_3_): δ 179.392, 162.645, 155.901, 148.246, 148.169, 136.865, 121.565, 87.325, 83.406, 68.576, 61.238, 55.231, 36.405, 19.161, 17.570, 17.408, 17.130, 17.034, 13.470, 13.154, 13.077, 12.560; HRMS (ESI-TOF) calcd. for C_27_H_46_N_6_O_7_Si_2_ [M + Na]^+^ 645.2859, found 645.2873.

*2′-Deoxy-2′-formamido-2-N-isobutyrylguanosine (* ***37*** *)* (40). Compound **36** (690 mg, 1.11 mmol, and 1 equiv.) was dissolved in tetrahydrofuran (THF, 11 ml). Triethylamine trihydrofluoride (0.903 ml, 5.54 mmol, 5 equiv.) was then added, and the mixture was stirred at room temperature. After stirring for 16 h, EtOAc and hexane were added to the reaction mixture. The reaction mixture was then filtered and washed with cold water and EtOAc. Compound **37** (395 mg, 1.04 mmol, 94% yield) was obtained. ^1^H-NMR (400 MHz, DMSO-*d*_6_): δ 12.093 (br, 1H), 11.656 (br, 1H), 8.243 (d, *J* = 8.8 Hz, 1H), 8.219 (s, 1H), 7.993 (s, 1H), 5.872 (d, *J* = 8.0 Hz, 1H), 5.813 (d, *J* = 8.8 Hz, 1H), 5.109 (t, *J* = 5.4 Hz, 1H), 5.050–4.993 (m, 1H), 4.219 (t, *J* = 3.8 Hz, 1H), 3.975–3.958 (m, 1H), 3.643–3.526 (m, 2H), 2.761 (sep, *J* = 7.0 Hz, 1H), 1.117 (d, *J* = 7.2 Hz, 6H); ^13^C-NMR (100 MHz, DMSO-*d*_6_): δ 180.131, 161.609, 154.790, 149.201, 148.247, 137.746, 120.016, 87.102, 84.623, 70.316, 61.466, 53.101, 34.761, 18.919; HRMS (ESI-TOF) calcd. for C_15_H_20_N_6_O_6_ [M + Na]^+^ 403.1337, found 403.1341.

*2′-Deoxy-2′-formamido-2-N-isobutyryl-5′-O-(4, 4′-dimethoxytrityl)guanosine (* ***38*** *)* (35). Compound **37** (390 mg, 1.03 mmol, 1 equiv.) were dissolved in pyridine (2.5 ml). DMTrCl (382 mg, 1.13 mmol, 1.1 eq.) and DMAP (2.51 mg, 0.0205 mmol, 0.02 eq.) was then added, and the mixture was stirred at room temperature. After stirring for 16 h, the reaction mixture was diluted with AcOEt and washed with a saturated NaHCO_3_ aqueous solution and brine. The organic layer was dried over Na_2_SO_4_, filtered, and concentrated *in vacuo*. The crude product was purified by silica gel column chromatography (AcOEt/MeOH = 10/1), and compound **38** (680 mg, 1.00 mmol, 97%) was obtained. ^1^H-NMR (400 MHz, DMSO-*d*_6_): δ 12.100 (s, 1H), 11.629 (s, 1H), 8.391 (d, *J* = 9.6 Hz, 1H), 8.078 (s, 1H), 8.059 (s, 1H), 7.377–7.181 (m, 9H), 6.833–6.797 (m, 4H), 5.906–5.864 (m, 2H), 5.235 (dd, *J* = 14, 8.4 Hz, 1H), 4.277–4.247 (m, 1H), 4.079 (m, 1H), 3.718 (s, 6H), 3.301 (dd, *J* = 10.6, 5.4 Hz, 1H), 3.168 (dd, *J* = 10.2, 3.4 Hz, 1H), 2.747 (sep, *J* = 6.9 Hz, 1H), 1.115 (d, *J* = 6.8 Hz, 6H); ^13^C-NMR (100 MHz, DMSO-*d*_6_): δ 180.140, 161.803, 158.076, 154.809, 149.176, 148.247, 144.779, 137.680, 135.428, 129.824, 127.831, 127.736, 126.739, 120.387, 113.126, 85.726, 85.391, 85.074, 70.081, 64.170, 55.021, 52.616, 34.787, 18.941; HRMS (ESI-TOF) calcd. for C_36_H_38_N_6_O_8_ [M + Na]^+^ 705.2643, found 705.2641.

*2′-Deoxy-2′-formamido-2-N-isobutyryl-5′-O-(4, 4′-dimethoxytrityl)guanosine-3′-O-(2-cyanoethyl N,N-diisopropylphosphoramidite) (* ***39*** *)* (36). Compound **38** (670 mg, 0.981 mmol, 1 eq.) were co-evaporated with toluene and dissolved in dichloromethane (10 ml). DIPEA (0.855 ml, 4.91 mmol, 5 equiv.), and 2-cyanoethyl-*N,N-*diisopropylchlorophosphoramidite (0.241 ml, 1.08 mmol, 1.1 eq.) were added at 0°C and stirred at room temperature. After stirring for 24 h, the reaction mixture was diluted with AcOEt and washed with a saturated NaHCO_3_ aqueous solution and brine. The organic layer was dried over Na_2_SO_4_, filtered, and concentrated *in vacuo*. The crude product was purified by silica gel column chromatography (DCM/ACN/TEA = 33/66/1→20/79/1) to obtain 39 (435 mg, 0.492 mmol, 50%). ^1^H-NMR (400 MHz, CDCl_3_): δ 11.932 (br, 1H), 8.244, 8.200 (2s, 1H), 7.796, 7.793 (2s, 1H), 7.580–7.188 (m, 9H), 6.836–6.775 (m, 4H), 6.742, 6.523 (2d, *J* = 8.4 Hz, 1H), 5.946–5.630 (m, 2H), 4.791–4.584 (m, 1H), 4.408–4.258 (m, 1H), 3.921–3.479 (m, 11H), 3.195–3.129 (m, 1H), 2.711–2.653 (m, 1H), 2.492–2.365 (m, 1H), 1.531–1.402 (m, 1H), 1.165 (d, *J* = 6.8 Hz, 6H), 1.109, 1.058 (2d, *J* = 6.8 Hz, 6H), 0.958, 0.883 (2d, *J* = 7.2 Hz, 3H), 0.764, 0.634 (2d, *J* = 7.0 Hz, 3H); ^13^C-NMR (100 MHz, CDCl_3_): δ 178.515, 161.719, 158.877, 155.691, 147.279, 147.165, 144.819, 138.648, 136.035, 130.245, 128.395, 128.224, 127.279, 122.797, 118.467, 113.431, 87.623, 86.411, 84.990, 72.439, 63.731, 58.448, 55.434, 53.583, 43.464, 36.158, 24.761, 20.746, 18.610; ^31^P-NMR (160 MHz, CDCl_3_): δ 151.612, 148.939; HRMS (ESI-TOF) calcd. for C_45_H_55_N_8_O_9_P [M + Na]^+^ 905.3722, found 905.3748.

*2′-Deoxy-2′-formamidouridine (* ***40*** *)* (46). Compound **5** (100.0 mg, 0.174 mmol, 1 equiv.) was dissolved in dichloromethane (15 ml). Trifluoroacetic acid (0.040 ml, 0.522 mmol, 3 equiv) was added, and the mixture was stirred for 3 h at room temperature. Hexane was added to the reaction solution and solids were precipitated. The solid was then suction-filtered and washed with hexane. The obtained solid was dried and compound **40** (46.7 mg, 0.172 mmol, 99%) was obtained. ^1^H-NMR (400 MHz, DMSO-*d*_6_): δ 11.285, (s, 1H), 8.145 (d, *J* = 9.2 Hz, 1H), 8.003 (s, 1H), 7.901 (d, *J* = 8.0 Hz, 1H), 5.895 (d, *J* = 8.8 Hz, 1H), 5.826 (d, *J* = 4.8 Hz, 1H), 5.688 (dd, *J* = 2.4, 8.4 Hz, 1H), 5.198 (t, *J* = 4.8 Hz, 1H), 4.524–4.465 (m, 1H), 4.075 (t, *J* = 5.2 Hz, 1H), 3.949–3.939 (m, 1H), 3.585 (m, 2H); ^13^C-NMR (100 MHz, CDCl_3_): δ 163.029, 161.650, 150.853, 140.631, 102.214, 86.856, 85.620, 70.599, 61.603, 53.076; HRMS (ESI-TOF) calcd. for C_10_H_13_N_3_O_6_ [M + Na]^+^ 294.0697, found 294.0662.

### Fluorescence measurement of Compound 31b and 33b

The samples were prepared at a final concentration of 30 mM in MeOH. A Cary Eclipse Fluorescence Spectrophotometer (Agilent) was used for fluorescence measurements. The prepared samples were transferred to a cell and the measurements were performed under the following conditions: excitation wavelength, 340 nm; excitation slit, 5 nm; emission slit, 5 nm; scan rate, 600 nm/min; averaging time, 0.1 s, date interval, 1.0 nm, and PMT detector voltage, 600 V. The obtained result was shown in Supplementary Figure S1.

### Synthesis of oligo RNA

The RNA used in this study was synthesized using an automated DNA synthesizer (NRs-4A10R7NP, Nihon Techno Service) at 0.2 μmol scale, DMTr on. The coupling time for 2′-formamide nucleoside amidite was increased from the typical 3 to 15 min due to potential lower coupling efficiency compared to natural RNA phosphoramidite. Under these adjusted conditions, the DNA synthesizer's trityl monitor indicated a coupling efficiency of approximately 100%. The phosphoramidite reagents for the natural nucleotide were as follows: 5′-DMT-2′-TOM-ribo adenosine (n-acetyl) OP (ANP-3201, ChemGenes), 5′-DMT-2′-TOM-ribo guanosine (n-acetyl) OP (ANP-3203, ChemGenes), 5′-DMT-2′-TOM-ribo cytidine (*n*-acetyl) OP (ANP-3202, ChemGenes), and 5′-DMT-2′-TOM-ribo uridine OP (ANP-3205, ChemGenes). The CPG supports for the natural nucleotide were as follows: 3′-TOM-ribo Adenosine (*n*-acetyl) 2′-lcaa CPG 1000 Å (N-3201–10, ChemGenes), 2′-TOM-ribo Guanosine (n-acetyl) 3′-lcaa CPG 1000 Å (N-3203-10, ChemGenes), 3′-TOM-ribo Cytidine (*n*-acetyl) 2′-lcaa CPG 1000 Å (N-3202-10, ChemGenes), and 3′-TOM-ribo Uridine 2′-lcaa CPG 1000 Å (N-3205-10, ChemGenes).

After RNA synthesis, 500 μl of 40% methylamine aqueous solution and 500 μl of 28% ammonia water were added, followed by incubation at 65°C for 15 min. After filtration (Millex-LCR, 0.45 μm) and drying in a centrifugal evaporator, 115 μl of DMSO was added and completely dissolved. Then, 60 μl of triethylamine and 75 μl of triethylamine trihydrofluoride were added and incubated at 65°C for 2.5 h to deprotect the silyl moiety. After incubation, diluted with 1.75 ml 0.1 M TEAA buffer (pH 7.0). To purify oligo RNA, a MicroPure II Column from BIOSEARCH TECHNOLOGIES was used. The column was washed with 4 ml of acetonitrile and MQ, and equilibrated with 4 ml of 0.1 M TEAA buffer (pH 7). The deprotected oligo RNA solution was then flowed, followed by 2 ml of 5% acetonitrile/95% 0.1 M TEAA buffer (pH 7) to remove incomplete oligonucleotides. After washing with 4 ml of MQ, the DMTr group was deprotected with 4 ml of 2% trifluoroacetic acid solution and washed with 10 ml of MQ. Finally, the target oligo RNA was eluted with 2 ml of a 50% acetonitrile aqueous solution. The eluted samples were evaporated in a centrifugal evaporator to remove acetonitrile and freeze-dried to obtain RNA. The purity of RNA was checked by HPLC, and identified by its molecular weight obtained by MALDI-TOF-MS (ultrafleXtreme, Bruker). The sequences, molecular weights, and isolation yields of the synthesized RNA are listed in Supplementary Table S1. HPLC chromatograms are shown in Supplementary Figure S2.

### Measurement of melting temperature of oligo RNA and siRNA

The samples of oligo RNA were prepared at a final concentration of 3 μM for each strand of oligo RNA, 10 mM sodium phosphate buffer (pH 7.0), and 1 M NaCl. And the samples of siRNA were prepared at a final concentration of 0.3 μM for each strand of siRNA, 10 mM sodium phosphate buffer (pH 7.0), and 25 mM NaCl. The prepared samples were heated at 90°C for 5 min and then annealed by slow cooling to room temperature. A JASCO V-650 Spectrophotometer was used for absorbance measurements. The prepared samples were transferred to a cell, and the absorbance at 260 nm at each temperature was measured by changing the temperature from 15 to 90°C at 0.5°C/min. The *T*_m_ value was calculated as the temperature at the inflection point of the sigmoid curve. The obtained melting curve is shown in Supplementary Figures S3, S4 and S8. Three measurements were taken, and the mean and standard error of the measurements were entered in the table.

### Measurement of thermodynamics parameter

Samples were prepared at final concentrations of 3, 6, 9 and 12 μM for each strand of oligo RNA, and the *T*_m_ value was measured as described above. In general, the relationship between *T*_m_ and RNA concentration is as follows (1), and the relationship between Δ*G*°, Δ*H*° and Δ*S*° is as follows (2). *R* is the gas constant, and *C*_t_ is the total concentration of single-stranded RNA.


(1) \begin{eqnarray*}\frac{1}{{{{T}_{\mathrm{m}}}}} &=& \frac{{R{\mathrm{\ ln}}\left( {{{C}_{\mathrm{t}}}/4} \right) + {\mathrm{\Delta }}S^\circ }}{{{\mathrm{\Delta }}H^\circ }} \nonumber\\ &=& \frac{{19.148\ {\mathrm{lo}}{{{\mathrm{g}}}_{10}}\left( {{{C}_{\mathrm{t}}}/4} \right)}}{{{\mathrm{\Delta }}H^\circ }} + \frac{{{\mathrm{\Delta }}S^\circ }}{{{\mathrm{\Delta }}H^\circ }}\end{eqnarray*}

(2) \begin{eqnarray*}{\mathrm{\Delta }}G^\circ = {\mathrm{\Delta }}H^\circ + T{\mathrm{\Delta }}S^\circ \end{eqnarray*}

From equation (1), the slope and intercept of the graph with 1/*T*_m_ on the vertical axis and log (*C*_t_/4) on the horizontal axis can be used to obtain Δ*H*° and Δ*S*°, respectively. These values were substituted into Equation (2) to obtain Δ*G*° at a certain temperature. The obtained melting curves are shown in Supplementary Figure S5, and the plot of 1/*T*_m_ versus log (*C*_t_/4) is shown in Supplementary Figure S6.

### Measurement of CD spectra

Samples were prepared at a final concentration of 2 μM for each strand of oligo RNA, 10 mM sodium phosphate buffer, and 100 mM NaCl. The prepared samples were heated to 90°C for 5 min and slowly cooled to room temperature. The samples were transferred to a 1 mm cell and the CD spectra were measured using a circular dichroism polarimeter (J-720WN). The measurement conditions were as follows: the measurement temperature was 20°C, the sensitivity was standard (100 mdeg), the starting wavelength was 350 nm, the ending wavelength was 200 nm, the data acquisition interval was 1 nm, the operation mode was continuous, the scanning speed was 500 nm/min, the response time was 0.5 seconds, and there were four integrations was 4 times.

### X-ray structural analysis of RNA

Crystallization was performed using the hanging-drop vapor diffusion method at 293 K. Crystallization droplets were prepared by mixing 0.2 μl of RNA solution and 0.2 μl of crystallization solutions (Supplementary Table S2). Single crystals were scooped with *LithoLoops^TM^* (Wakenbtech Co. Ltd., Japan) and directly flash-cooled in liquid nitrogen prior to the X-ray experiment.

X-ray data were collected at 100 K using synchrotron radiation at the BL-17A beamline of the Photon Factory (Tsukuba, Japan). The datasets were processed and scaled using *XDS* (47). The statistics of data collection are summarized in Supplementary Table S3.

Initial phases were determined by the Molecular Replacement method with the program *AutoMR* from the *Phenix* suite (48,49), using an A-form RNA duplex constructed with the COOT program (50,51) as a probe. The atomic parameters of each structure were refined using the program *refine.phenix* from the *Phenix* suite (48,52) through a combination of simulated annealing, crystallographic conjugate gradient minimization refinements, and B-factor refinements. The statistics for the structural refinements are summarized in Supplementary Table S3. The atomic coordinates and experimental data of the RNA crystal were deposited in the Protein Data Bank (PDB) with the ID code 8YNO.

### Construction of the luciferase reporters

All reporter plasmids were constructed using psiCHECK-1 (Promega). Oligonucleotides with a target sequence that was completely matched (CM) to the siRNA guide strand were chemically synthesized with cohesive Xhol/EcoRI ends. They were annealed and inserted into psiCHECK-1 at the corresponding restriction enzyme site, and named psiCHECK_gCM. Similarly, psiCHECK with three tandem repeats of seed-matched (SM) sequences to the siRNA guide strand, which is complementary to the 8 nt long seed-containing sequence but not to the non-seed region, was also generated and named psiCHECK-gSM. Each of the inserted targets was expressed as part of the 3′-untranslated regions (UTRs) region of the *Renilla* luciferase mRNA in the transfected cells.

### Cell culture and RNA silencing activity assay

Human HeLa cells were cultured in Dulbecco's modified Eagle's medium (D-MEM) (FUJIFILM Wako, Osaka, Japan) with 10% heat-inactivated fetal bovine serum (FBS) (Gibco Life Technologies, Paisley, UK) and 1% Penicillin-Streptomycin Solution (PS) (FUJIFILM Wako) at 37 °C with 5% CO_2_. The cells were diluted to 1.0 × 10^5^ cells/ml in DMEM containing 10% FBS and 1% PS. They were then dispensed in 1 ml per well of a 24-well cell culture plate and incubated at 37°C for 16 h. After that, all culture media were removed, and 250 μl of DMEM without FBS and PS was added. pGL3-Control (Promega) (100 ng), psiCheck-gCM or -gSM (10 ng), and siRNA duplexes (0.005, 0.05, or 0.5 nM) were transfected simultaneously using 1 μl Lipofectamine 2000 (Thermo Fisher Scientific, Waltham, MA, USA) per well. After 4 h, the medium was removed and 1 ml of DMEM containing 10% FBS and 1% PS was added to each well. 24 hours after transfection, all media were removed from the 24-well cell culture plates. Then, 100 μl of Passive Lysis Buffer (Promega) was added per well and shaken for 30 min using a 2D platform rocker. After overnight storage at -80°C, the plate was shaken for 30 min using a 2D platform rocker to dissolve the solution. Relative luciferase activity (*Renilla* luciferase activity/firefly luciferase activity) was measured using a dual-luciferase reporter assay system (Promega). pGL3-Control, which encodes firefly luciferase, served as a control for the calculation of relative luciferase activity. The siRNAs used against mammalian endogenous genes were siVIM [human vimentin]-270, siCTLC [human clathrin heavy chain]-2416, siKIF23 [human kinesin family member 23]-430, and siMC4R [human melanocortin 4 receptor]-490. RNA strands of siRNA without modifications, as shown in Figure 3, were chemically synthesized and purchased from GenePharma (Shanghai Gene Pharma, Shanghai, China). The percentages of relative values for each siRNA were calculated using siCont, an siRNA for GFP knockdown, as 100%.

### Microarray analysis

HeLa cells were cultured at a density of 1.0 × 10^5^ cells/ml in a well of 24-well plate, and 50 nM siRNA was transfected with Lipofectamine 2000. Twenty-four hours post-transfection, total RNA was isolated from the cells using the RNeasy Mini Kit (QIAGEN, Germany), and its quality was checked with NanoDrop 2000 (Thermo Fisher Scientific) and Bioanalyzer (Agilent Technologies, Santa Clara, CA, USA). For microarray analysis, cDNA synthesis and Cy-3 labeling were performed using the One-Color Quick Amp Labeling Kit (Agilent Technologies). Labeled RNA was fragmented and hybridized to the SurePrint G3 Human GE v3 8 × 60K Microarray (Agilent Technologies) at 65 °C for 17 hours. After hybridization, the microarray was washed, and scanned with a DNA Microarray Scanner. Data processing was carried out using Feature Extraction software v12.1.1.1 (Agilent Technologies), employing default settings (protocol GE1_1200_Jun14 and Grid 02363_D_F_20221108). In this process, the background was subtracted and processed signal intensities were spatially detrended. For the comprehensive analysis of the dataset, selection criteria were applied to include only the transcript data fulfilling the following conditions: ControlType = 0, gIsPosAndSignif = 1, gIsFeatNonUnifOL = 0, gIsWellAboveBG = 1, gIsSaturated = 0, gIsFeatPopnOL = 0, and SystematicName = NM_Identifier. Total of 15, 434 transcripts for siKIF23-430 and 15, 753 for siVIM-270 met these criteria. Among them, 164 transcripts for siKIF23-430 and 1, 377 for siVIM-270 were used as off-target transcripts, since they have sequences complementary to the seed region of each siRNA in their 3′ UTRs. Mock-transfected cells were used as controls. Normalization was done using the quantile method (53), and results were displayed in MA plots and cumulative distributions.

### RNA-sequencing (RNA-seq)

For RNA-seq analysis, total RNAs prepared for microarray experiments were used. RNA sequencing was performed using a DNBSEQ-G400 Platform (BGI). mRNA libraries were sequenced with a sequencing depth of at least 19 million paired 150-bp reads. RNA-Seq data was mapped using Hisat2 (v2.2.1) (54) against the human genome sequence and annotated gene models (GRCh38, release 105) from Ensembl (http://www.ensembl.org/). Gene expression levels were quantified as transcripts per million (TPM) using featureCounts (v2.0.1) with default settings (55). Before analysis, genes with a TPM value below 10 were excluded, and a total of 8, 752 transcripts was used for analysis. Among them, 676 transcripts with sequences complementary to the seed region of siVIM-270 in the 3′ UTR were designated as seed-matched transcripts, while the remaining 8, 076 transcripts without complementary sequences were designated as non-seed matched transcripts. Mock-transfected cells were used as controls, and results were displayed in MA plots and cumulative distributions.

### Quantitative RT-PCR (qRT-PCR)

To evaluate the effects of siRNAs on the endogenous genes, qRT-PCR was performed. For analyses of dose-dependent effects of siRNAs, HeLa cells were cultured at a density of 1.0 × 10^5^ cells/ml in a well of a 24-well plate, and 0.05, 0.5, 5 or 50 nM siRNA was transfected with Lipofectamine 2000. Twenty-four hours post-transfection, total RNA was isolated from the cells using ISOGEN (NIPPON GENE), and its quality was checked with NanoDrop 2000 (Thermo Fisher Scientific). For validation of microarray data, total RNAs for microarray analyses were used. An aliquot of total RNA (2 μg) from each sample was reverse transcribed using the High-Capacity cDNA Reverse Transcription Kits (Applied Biosystems, Foster City, CA, USA), according to the manufacturer's instructions. The qRT-PCR was performed with KAPA SYBR Fast qPCR Kit (NIPPON Genetics) using the QuantStudio^TM^ 3 Real-Time PCR System (Applied Biosystems) through the ΔΔCt method. Firstly, the expression levels of the target genes were normalized by the endogenous reference gene, glyceraldehyde-3-phosphate dehydrogenase (GAPDH). Subsequent normalization was carried out against the mock-transfected samples. Sequences of the used primer sets were shown in Supplementary Table S4.

## Results and discussion

### Design and chemical synthesis of 2′-formamidonucleoside phosphoramidites

We designed and introduced 2′-formamidonucleoside phosphoramidites into the RNA strands. While 2′-formamidonucleotide triphosphate and nucleosides have been synthesized previously (56,57), their incorporation into oligonucleotides has not been reported, leaving their physical properties unknown. The presence of amino and carbonyl groups can potentially result in more hydrogen bonds. These hydrogen bond possibilities led to the expectation of new interactions during base pairing or protein binding. We anticipated that these subtle steric factors and changes in interaction patterns would confer different stabilities to the RISC formation process when bound to Ago2 compared to natural RNA, thereby reducing off-target effects.

**Scheme 1.**
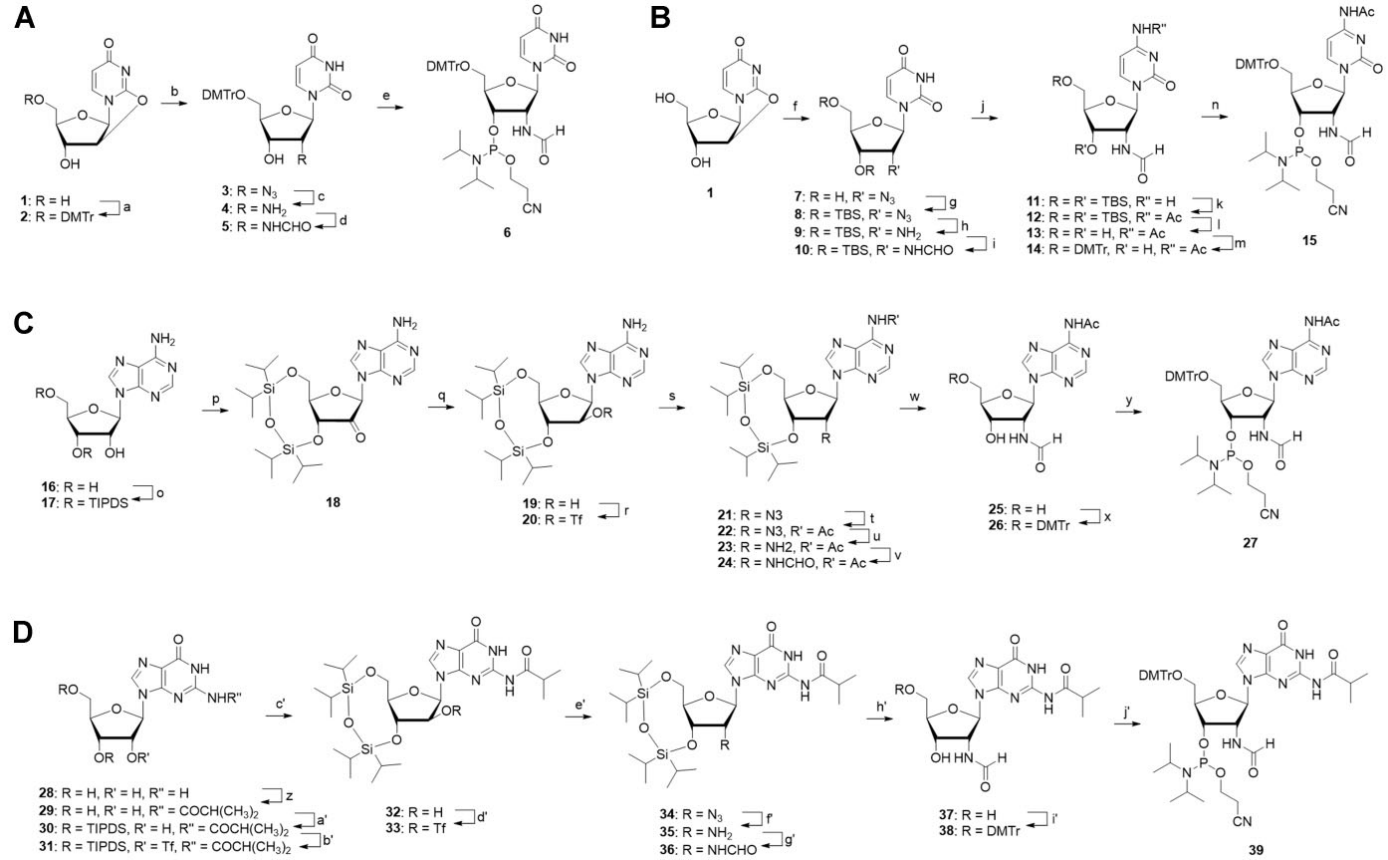
Synthesis of 2′-formamidonucleoside phosphoroamidite analogs. Reagents and conditions: (a) DMTrCl, DMAP, pyridine, rt, 90%; (b) NaN_3_, 15-crown-5, DMF, 120°C, 65%; (c) H_2_, Pd/C, MeOH, rt, 91%; (d) formic acid, EDC-HCl, DMAP, DIPEA, DCM, rt, 57%; (e) 2-cyanoethyl-*N, N*-diisopropylchlorophosphoroamidite, DIPEA, DCM, 0°C, 29%; (f) NaN_3_, 15-crown-5, DMF, 120°C; (g) TBDMSCl, imidazole, DMF, rt, 41% (over 2 steps); (h) H_2_, Pd/C, MeOH, rt, 99%; (i) formic acid, EDC-HCl, DMAP, DIPEA, DCM, rt, 85%; (j) TPSCl, TEA, DMAP, NH_4_OH, ACN, 0°C, 87%; (k) acetic anhydride, pyridine, rt; (l) TEA-3HF, THF, rt, 65% (over 2 sateps); (m) DMTrCl, DMAP, pyridine, rt, 95%; (n) 2-cyanoethyl-*N,N*-diisopropylchlorophosphoroamidite, DIPEA, DCM, 0°C, 76%; (o) TIPDSCl_2_, pyridine, rt, 99%; (p) CrO_3_, pyridine, acetic anhydride, DCM, rt; (q) NaBH_4_, EtOH/H_2_O, 0°C, 29% (over 2 steps); (r) *N*-phenylbis (trifluoromethanesulfonimide), DMAP, DCM, 0°C, 97%; (s) NaN_3_, DMF, 60°C; (t) acetyl chloride, pyridine, rt, 63% (over 2 steps); (u) H_2_, Pd/C, MeOH, rt, 68%; (v) formic acid, EDC-HCl, DMAP, DIPEA, DCM, rt, 91%; (w) TEA-3HF, THF, rt, 98%; (x) DMTrCl, DMAP, pyridine, rt, 97%; (y) 2-cyanoethyl-*N,N*-diisopropylchlorophosphoroamidite, DIPEA, DCM, 0°C, 63%; (z) 1) TMSCl, pyridine 2) isobutyryl chloride, 3) NH_4_OH, rt, 93%; (a’) TIPDSCl_2_, pyridine, rt, 96%; (b’) CF_3_SO_2_Cl, DMAP, DCM, 0°C, 28%; (c’) CF_3_COOK, DIPEA, DMF, 80°C, 53%, (d’) CF_3_SO_2_Cl, DMAP, DCM, 0°C, 20%; (e’) NaN_3_, DMF, rt, 95%; (f’) H_2_, Pd/C, MeOH, rt, 53%; (g’) formic acid, EDC-HCl, DMAP, DIPEA, DCM, rt, 96%; (h’) TEA-3HF, THF, rt, 94%; (i’) DMTrCl, DMAP, pyridine, rt, 97%; (j’) 2-cyanoethyl-*N,N*-diisopropylchlorophosphoroamidite, DIPEA, DCM, 0°C, 50%.

The synthesis of the phosphoramidites of 2′-formamidonucleoside followed the process outlined in Scheme 1. While the specific synthetic pathways varied for each of the four bases, the general synthetic route proceeded as follows: Initially, an azido group was incorporated at the 2′ position, which was subsequently reduced to an amino group. Subsequently, the amino group underwent condensation with formic acid to produce the formamido group. Detailed information on the synthesis can be found in the Materials and methods section and Supporting Information. Consequently, the desired 2′-formamidonucleoside phosphoramidites for all four bases were successfully obtained. The synthesized phosphoramidite was stored at −30°C in an argon atmosphere, similar to the commercially available normal RNA amidites. For larger-scale synthesis, the methodologies for the adenosine and guanosine analogs will need to be explored due to the extended synthesis steps and certain stages with low yields.

### Thermodynamic evaluation of oligo RNAs with 2′-formamidonucleoside

We synthesized oligo RNAs with 2′-formamidonucleoside phosphoramidites (X^f^) of each base, introducing X^f^ centrally using an automated nucleic acid synthesizer. The sequences were designed as 11-mers to clearly observe the changes in the melting temperature (*T*_m_) and to avoid self-complementary strands and hairpin structures.

Initially, we analyzed the change in double-strand stability of RNA due to the introduction of 2′-formamidonucleoside (58). The sequences used to analyze the *T*_m_ values and results are listed in Table 1. Regardless of the base type, it became clear that the introduction of 2′-formamidonucleoside decreased the *T*_m_ values by 6.0–7.6°C per modification. Comparing the U–A pairs (Entries 2 and 3) and C-G pairs (Entries 6 and 7), the decrease in *T*_m_ values was smaller for C-G pairs, which might be due to the higher number of hydrogen bonds in C–G pairs, resulting in stronger base pairing and less impact from sugar modification. Moreover, when base pairing involved 2′-formamidonucleosides on both strands (Entries 4 and 8), the decrease in *T*_m_ values for both U–A and C–G pairs was close to the sum of the values when only one strand was modified. This suggests that 2′-formamidonucleosides independently decrease double-strand stability without new interactions between them. From these results, it became evident that the introduction of 2′-formamidonucleosides decreased the double-strand stability of RNA, and it is anticipated that incorporating 2′-formamidonucleosides into the seed region of siRNAs could suppress off-target effects.

**Table 1.** *T*_m_ value of the double strand RNAs with modification

Entry	Sequence^a^	*T*_m_ (°C)^b^	Δ*T*_m_ (°C)
1	5′-ACUGCUACGAU-3′ 3′-UGACGAUGCUA-5′	66.2 ± 0.1	-
2	5′-ACUGC**U^f^**ACGAU-3′ 3′-UGACGAUGCUA-5′	58.6 ± 0.2	−7.6
3	5′-ACUGCUACGAU-3′ 3′-UGACG**A^f^**UGCUA-5′	59.7 ± 0.1	−6.5
4	5′-ACUGC**U^f^**ACGAU-3′ 3′-UGACG**A^f^**UGCUA-5′	52.2 ± 0.2	−14.0
5	5′-GUCAUCGUAGC-3′ 3′-CAGUAGCAUCG-5′	67.6 ± 0.1	-
6	5′-GUCAU**C^f^**GUAGC-3′ 3′-CAGUAGCAUCG-5′	61.6 ± 0.1	−6.0
7	5′-GUCAUCGUAGC-3′ 3′-CAGUA**G^f^**CAUCG-5′	61.5 ± 0.1	−6.1
8	5′-GUCAU**C^f^**GUAGC-3′ 3′-CAGUA**G^f^**CAUCG-5′	55.2 ± 0.3	−12.4

^a^ **X^f^**: 2′-formamidonucleoside,

^b^Three measurements were taken, and the mean and standard error of the measurements were shown.

Next, we investigated the impact on the base recognition capability using the sequences from entries 1 and 2 in Table 1. One of the factors that reduce off-target effects in the seed region is the base recognition ability of RNA strands containing non-natural nucleic acids (59). We investigated the mismatch recognition ability of 2′-formamidonucleosides by measuring the *T*_m_. *T*_m_ values were measured for sequences where mismatched bases were introduced at positions forming base pairs with 2′-formamidouridine. The results are presented in Table 2. Sequences with mismatch pairs exhibited a Δ*T*_m_ that was generally 1–2°C lower than that of natural RNA, indicating a slight decrease in the base recognition ability.

**Table 2.** *T*_m_ value and of the double strand RNAs with mismatch base pair

Entry	Sequence^a^	*T*_m_ (°C)^b^	Δ*T*_m_ (°C)
1	5′-ACUGCUACGAU-3′ 3′-UGACGAUGCUA-5′	66.2 ± 0.1	-
9	5′-ACUGCUACGAU-3′ 3′-UGACG**U**UGCUA-5′	51.4 ± 0.1	−14.8
10	5′-ACUGCUACGAU-3′ 3′-UGACG**G**UGCUA-5′	61.7 ± 0.2	−4.5
11	5′-ACUGCUACGAU-3′ 3′-UGACG**C**UGCUA-5′	51.2 ± 0.1	−14.0
2	5′-ACUGC**U^f^**ACGAU-3′ 3′-UGACGAUGCUA-5′	58.6 ± 0.2	-
12	5′-ACUGC**U^f^**ACGAU-3′ 3′-UGACG**U**UGCUA-5′	44.4 ± 0.0	−14.2
13	5′-ACUGC**U^f^**ACGAU-3′ 3′-UGACG**G**UGCUA-5′	56.7 ± 0.1	−1.9
14	5′-ACUGC**U^f^**ACGAU-3′ 3′-UGACG**C**UGCUA-5′	44.5 ± 0.3	−14.1

^a^ **U^f^**: 2′-formamidouridine.

^b^Three measurements were taken, and the mean and standard error of the measurements were shown.

Subsequently, the thermodynamic parameters were calculated for the same sequences from entries 1 and 2 in Table 1 (29). Table 3 presents the results. The experimental outcomes revealed that the absolute value of Δ*G*° for the modified RNA was 5.0 kcal/mol smaller than that of natural RNA. Furthermore, the modified sequences showed an increase in ΔΔ*H*° by 35.8 kcal/mol and an increase in ΔΔ*S*° by 98.9 kcal/mol when compared with natural RNA. These results indicate that the introduction of modifications makes the formation of double strands entropically favorable but enthalpically unfavorable, and the impact of enthalpic destabilization is greater, thereby destabilizing the formation of double strands.

**Table 3.** Thermodynamic parameters of the double strand RNAs

Entry	Sequence^a^	Δ*H*°^b^ (ΔΔ*H*°) (kcal/mol)	Δ*S*°^b^ (ΔΔ*S*°) (cal/mol·K)	Δ*G*°_37°C_^b^ (ΔΔ*G*°_37°C_) (kcal/mol)
1	5′-ACUGCUACGAU-3′ 3′-UGACGAUGCUA-5′	−118.8 ± 4.7	−350.9 ± 13.7	−10.0 ± 0.5
2	5′-ACUGC**U^f^**ACGAU-3′ 3′-UGACGAUGCUA-5′	−104.1 ± 6.6 (+14.7)	−314.5 ± 19.9 (+36.4)	−6.6 ± 0.5 (+3.4)

^a^ **U^f^**: 2′-formamidouridine.

^b^Three measurements were taken, and the mean and standard error of the measurements were shown.

### The structure of oligonucleotides containing 2′-formamidonucleoside

The introduction of a modification to the sugar backbone is thought to affect the sugar conformation. The conformation of 2′-formamidouridine was analyzed by ^1^H-NMR coupling. By measuring ^1^H-NMR and applying the value of *J*_1'-2′_ to the formula (C3'-endo (%) = 100 – *J*_1'-2′_ × 10), the ratio of C3'-endo to C2'-endo conformations could be analyzed (60). Compound **40**, the monomer of formamidouridine, was synthesized according to Supplementary Scheme S2 by detritylation of compound **5** (46). For comparison, the sugar conformations of commercially available deoxyuridine and uridine were examined in the same manner. ^1^H-NMR was measured in DMSO-*d_6_*, and the obtained *J*_1'-2′_ values are presented in Table 4. 2′-Foramidouridine showed a 12% presence ratio of C3'-endo, which is lower than the 32% of 2′-deoxyuridine, indicating that 2′-formamidouridine has a smaller proportion of the C3'-endo conformation.

**Table 4.** Conformation of the sugar backbone

	*J*_1'-2′_ (Hz)	C3'-endo (%)
2′-deoxyuridine	6.8	32
uridine	5.6	44
2′-formamidouridine	8.8	12

Next, we present the results of CD (circular dichroism) spectroscopy analysis using double-stranded RNA from entries 1 and 2 in Table 1, as shown in Supplementary Figure S7. No significant differences were observed between the modified double-stranded RNA and the natural RNA. This is likely because converting only one nucleoside of an 11-mer oligo RNA to a modified form does not significantly affect the overall structure of the RNA.

**Figure 2.**
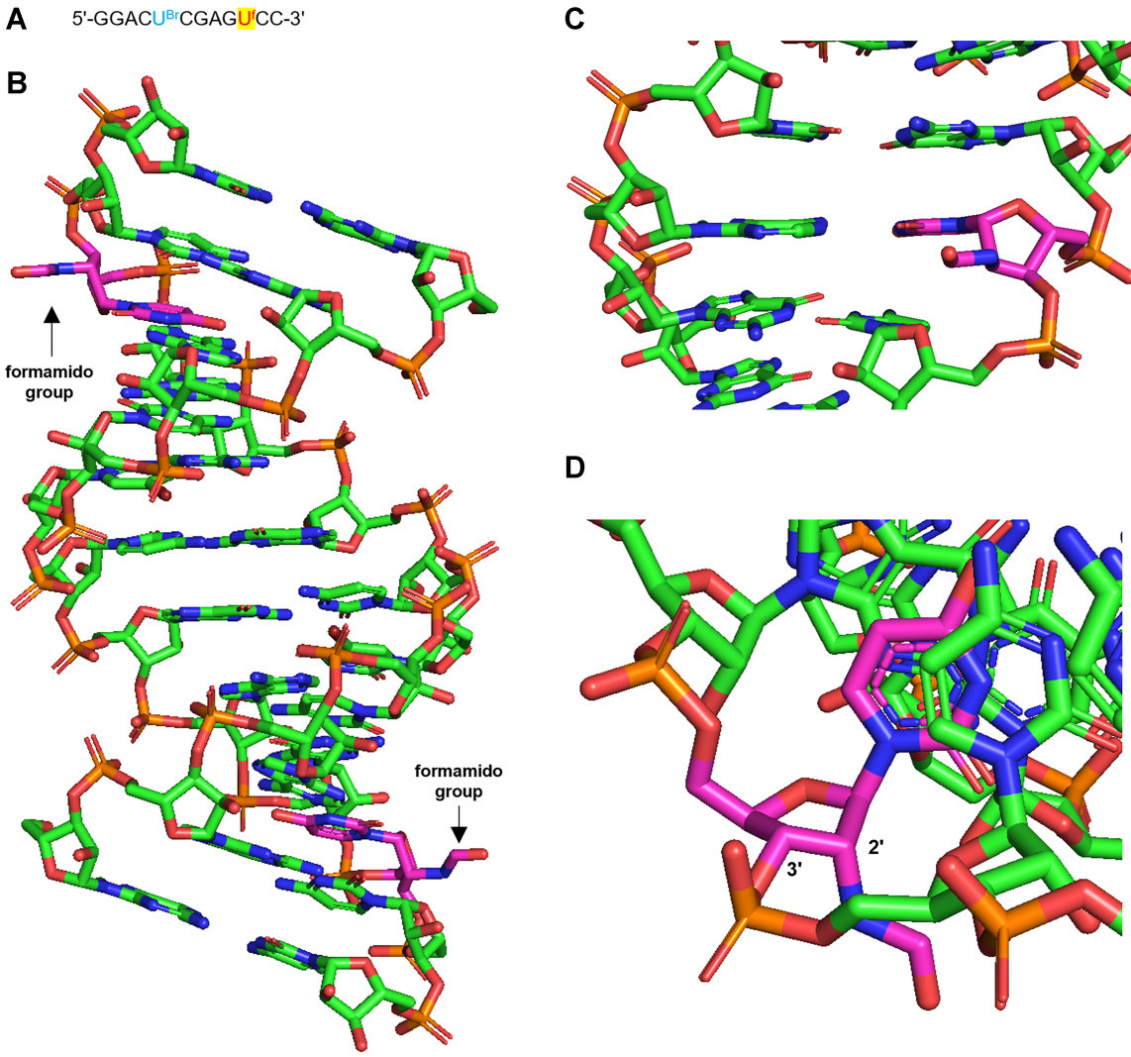
Crystal structure of double-stranded RNA with 2′-formamidouridine. (**A**) Sequence for crystal structure analysis. U^Br^ is 5-bromouridine and U^f^ is 2′-formamidouridine. This is a self-complementary sequence. (**B**–**D**) The result of X-ray structural analysis. The magenta-colored nucleoside was 2′-formamidourisine. (**B**) Overall structure of double-stranded RNA. (**C**) Base pairing of 2′-formamidouridine. (**D**) The sugar conformation of 2′-formamidouridine in double strand RNA.

Furthermore, X-ray structural analysis was conducted on double-stranded RNA with 2′-formamidonucleoside. A self-complementary RNA strand containing formamidonucleoside was designed and synthesized (Figure 2A). Notably, the introduction of 5-bromouridine allows the bromine atom to act as an anomalous scatterer, enabling structural determination via anomalous dispersion methods. The results of the crystal structure analysis using the synthesized RNA strands are shown in Figure 2B-D. Observing the overall structure of the double-stranded RNA (Figure 2B), it was evident that the formamido group protruded into the minor groove. When examining the base-pairing region (Figure 2C), base pairing was confirmed, similar to that in other natural nucleosides. Moreover, the sugar backbone (Figure 2D) also adopts a 3′-endo conformation, akin to that of other nucleosides. From these results, it can be inferred that the introduction of the formamido group at the 2′ position does not sterically hinder double-strand formation, nor does it significantly alter the orientation of the bases or the structure of the sugar backbone, making it unlikely that these factors contributed to the decreased stability of the double strands.

Based on the results of the conformational analysis of the monomer, thermodynamic parameters, CD spectrum, and X-ray structural analysis, several conclusions can be drawn regarding the 2′-formamidonucleoside.

The introduction of a 2′-formamido group makes the monomer unit more prone to adopt a DNA-like 2′-endo conformation. However, when incorporated into double-stranded RNA, 2′-formamidonucleosides assume an RNA-like 3′-endo structure. This means that it is entropically unfavorable due to the conformational change in the sugar backbone. Meanwhile, during the association of the double strands, it is thought that the hydrogen bonds between the formamido group and the hydrated water molecules are broken and dissociated. 2′-formamide groups in natural RNA can interact with a greater number of water molecules than hydroxyl groups. Typically, the cleavage of intermolecular hydrogen bonding is enthalpically unfavorable but entropically favorable. Taking into account these changes in thermodynamic parameters and the experimental Tm results, it is suggested that the decrease in Tm resulting from the introduction of formamide groups is primarily due to the interaction of formamide groups with numerous water molecules in single-stranded RNA. This interaction requires more energy to dissociate the water molecules during double-strand formation.

### Evaluation of on- and off-target effect of siRNA containing 2′-formamidonucleosides

**Figure 3.**
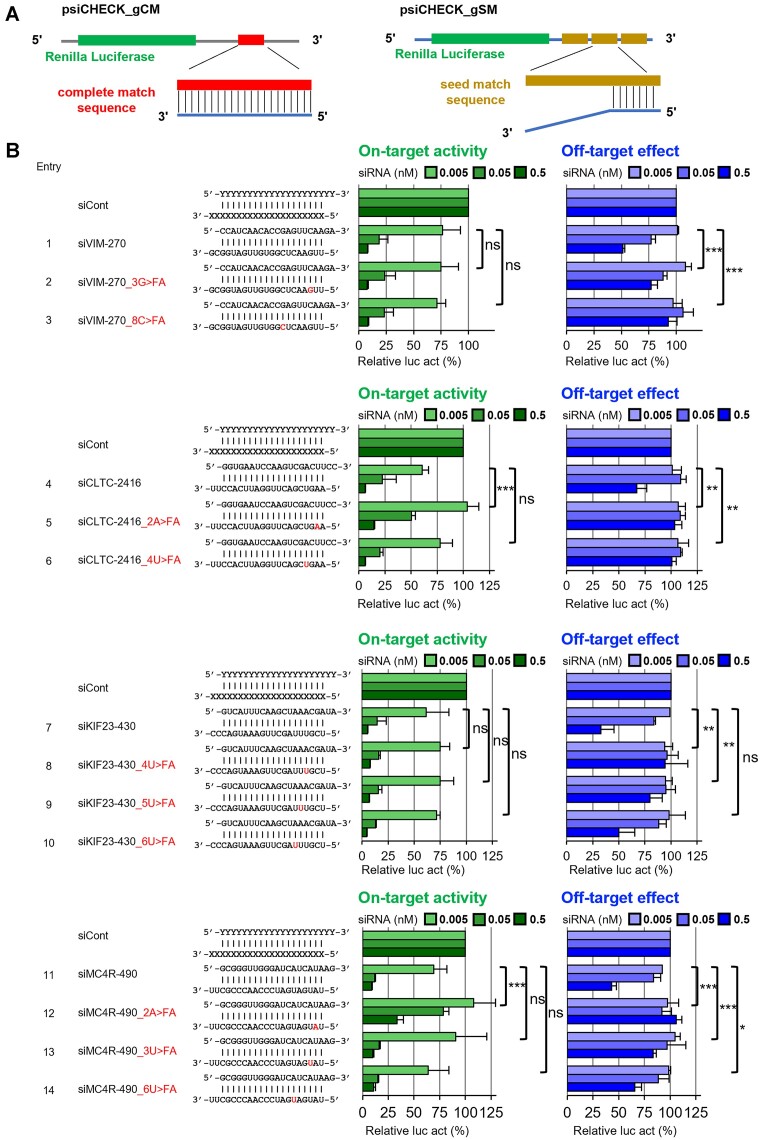
RNAi on-target activity and off-target effects. (**A**) Vector design for evaluating on-target and off-target effects. (**B**) On-target activity and off-target effects. Upper RNA strand indicates the passenger strand, and the lower indicates the guide strand. Green graphs show on-target activity. The blue graphs show off-target effects. In the sequence, the red text indicates the position of the modifier. Relative luc activity (%) was calculated using the activity of *Renilla* luciferase and firefly luciferase transfected as a control. siRNA against green fluorescent protein (GFP) was used as control siRNA (siCont). The *P*-values were calculated using a Two-Way ANOVA test. Significance levels are indicated as follows: *P < 0.05, **P < 0.01, ***P < 0.001. Each experiment was repeated three times.

We evaluated the RNAi on- and off-target effects of natural siRNA and siRNA with a 2′-formamidonucleoside introduced in the seed region by reporter assays. *Renilla* luciferase mRNA was used as the target sequence. As shown in Figure 3A, the psiCHECK-gCM vector, with a complete-match sequence to the guide RNA in its 3′-UTR region, was used to assess the RNAi on-target activity. Conversely, psiCHECK-gSM, containing sequences in the same region that form base pairs only with the seed region of siRNA and repeated three times, was used to evaluate off-target effects (24). siRNAs with formamidonucleosides introduced into each position in the seed region were designed and synthesized as shown in Figure 3B. The *T*_m_ measurements for these siRNAs are detailed in Supplementary Table S5. It has been verified that the incorporation of formamido modifications, as in the model sequence, resulted in a reduction in *T*_m_ values. This reduction was particularly significant when the modification was introduced at an internal position. Using these siRNAs, we assessed the concentration-dependent effects (0.005–0.5 nM) on both on- and off-target activities, the results of which are presented in Figure 3B.

For the RNAi activity, all siRNA with the formamidonucleoside in the seed region maintained on-target activity compared to the unmodified siRNA. However, siRNA with 2′-formamidonucleoside at position 2 reduced RNAi on-target activities about 2 to 5 fold (Figure 3B Entry 5, 12). The observed reductions in the on-target activity is consistent with what has been previously reported with bulky 2′-modifiations such as 2′-*O*-(methylthiomethoxy)methyl uridine and 2′-*O*-methoxyethyl uridine at position 2 of the guide strand (61).

In contrast, all siRNAs with the formamidonucleoside, except for siRNA with the formamidonucleoside at position 6, decreased off-target activity almost completely compared to unmodified siRNA (Figure 3B). This demonstrate that formamido derivatives of all four nucleosides (A, G, C and U) at positions other than 2nd position can effectively decrease the off-target activity without compromising the on-target activity. Since the siRNA seed region with the low *T*_m_ value is reported to enable the suppression of off-target effect, the formamidonucleosides may cause a decrease in off-target activity by lowering *T*_m_ in the duplex between siRNA seed region and target mRNA (19). However, the degree of suppression of the off-target activity may varies depending on the siRNA sequence in addition to the position of the modification. The siMC4R-490_6U > FA decreased the off-target activity, but the same modification in the siKIF23 (siKIF23-430_6U > FA) only slightly reduced the off-target activity (Figure 3B Entry 10, 14). Observing the effect of formamidonucleoside in on-target and the off-target activity varies depending on the siRNA sequence and/or position, we hypothesized that when siRNA binds to Ago2, the introduction of a 2′-formamido group interacts differently than a hydroxyl group, thereby altering the structure of guide RNA on Ago2.

Future studies should investigate in more detail the differences in off-target effect suppression based on the modification site, and explore varying the number of modifications introduced.

### Genome-wide evaluation of off-target activities

**Figure 4.**
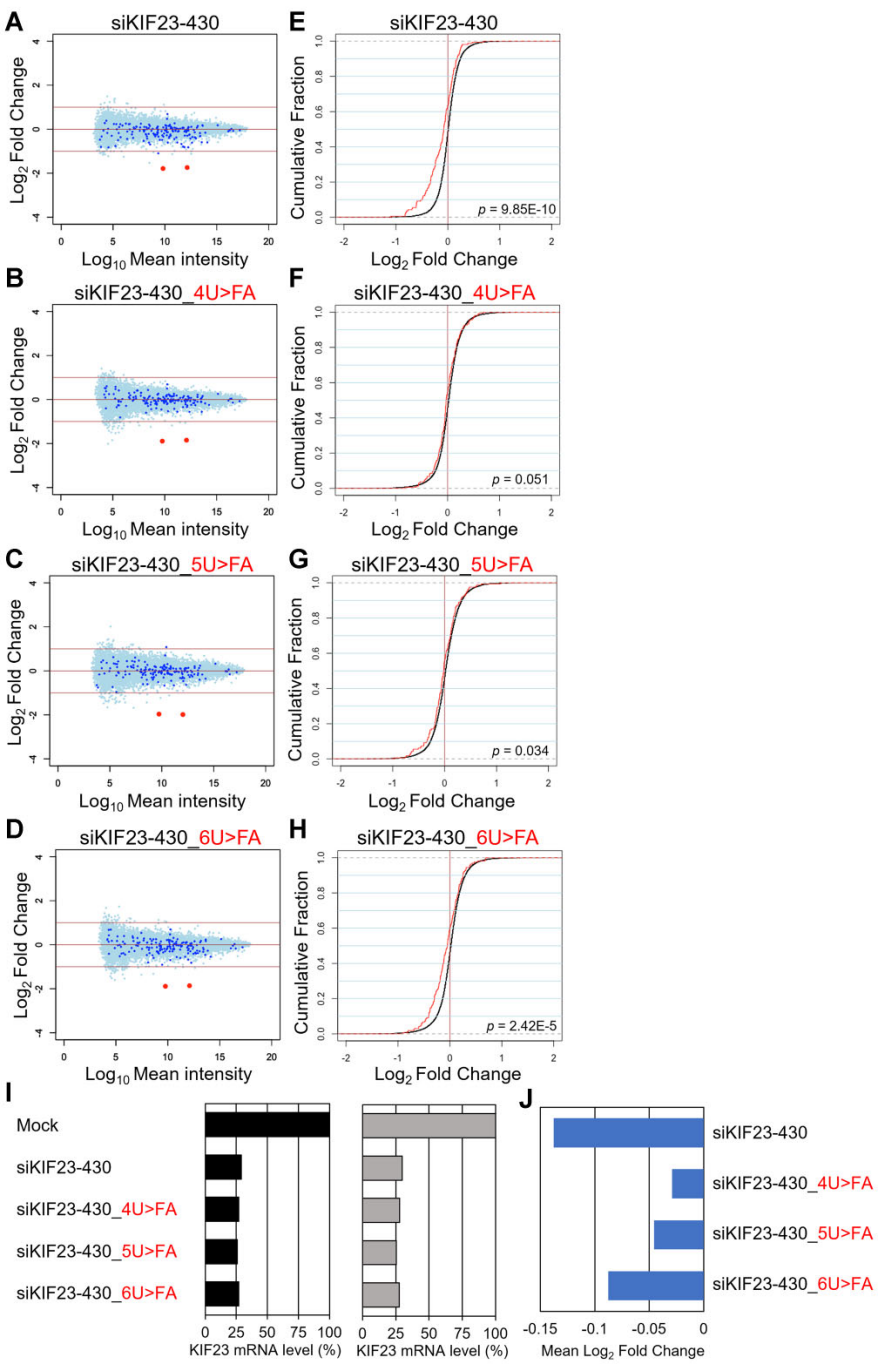
Microarray profiling of on-target and off-target effects of siKIF23-430 with 2′-formamido modifications. (**A**-**D**) MA plots showing log_2_ fold changes (vertical lines) and average log_10_ signal intensities (horizontal lines) of the expression levels of transcripts from the cells transfected with siKIF23-430 and its variants versus those from mock transfected cells. Dark blue dots represent 164 transcripts with siKIF23-430 SM sequence (off-target transcripts) in their 3′ UTRs, light blue dots represent 15 270 other transcripts without SM sequence (not off-target transcripts), and red dots denote two KIF23 splice variants (RefSeq: NM_001367804 and NM_138555, from the left). (**E**-**H**) Cumulative distributions of transcripts showing log_2_ fold changes (horizontal lines) and cumulative fractions (vertical lines). Red and black lines show the results for off-target and non-off-target transcripts, respectively. *P*-values were obtained using Wilcoxon rank-sum test. (**I**) The expression levels of two splice variants of *KIF23* calculated by signal intensities compared to mock (black for RefSeq: NM_001367804, gray for NM_138555). (**J**) Seed-dependent off-target effects shown by mean log_2_ fold change for unmodified siKIF23-430 and 2′-formamido-modified siKIF23-430s at positions 4, 5, or 6.

**Figure 5.**
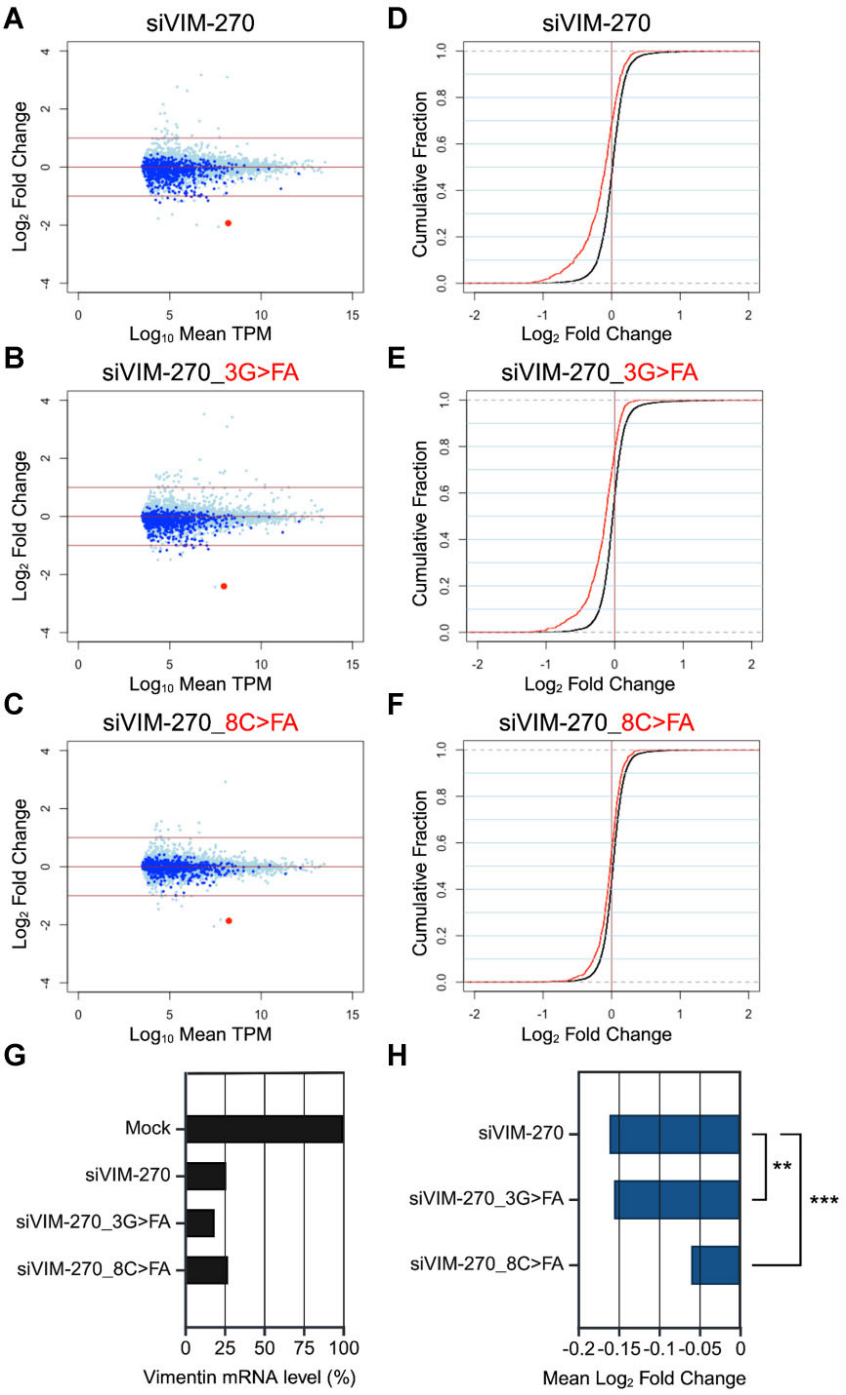
RNA-seq analysis of on-target and off-target effects of siVIM-270 with 2′-formamido modifications (**A**-**C**). MA plots showing log_2_ fold changes (vertical axis) and average log_10_ transcripts per million (TPM) (horizontal axis) of the expression levels of transcripts from cells transfected with siVIM-270 and its variants versus those from mock-transfected cells. Dark blue dots represent 676 transcripts with the siVIM-270 SM sequence (off-target transcripts) in their 3′ UTRs, while light blue dots represent 8076 other transcripts without the SM sequence (not off-target transcripts). (**D**-**F**) Cumulative distributions of transcripts showing log_2_ fold changes (horizontal axis) and cumulative fractions (vertical axis). Red and black lines show the results for off-target and non-off-target transcripts, respectively. (**G**) *VIM* mRNA levels calculated by TPM compared to mock. (**H**) Seed-dependent off-target effects shown by mean log_2_ fold change for unmodified siVIM-270 and 2′-formamido-modified siVIM-270s at positions 3 or 8. Statistical significance for each group was determined by comparing the cumulative distribution differences of siRNA SM transcripts using the Wilcoxon rank-sum test (**P < 0.01, ***P < 0.001).

To evaluate the 2′-formamido modifications of siRNAs on RNAi of the endogenous genes, the dose-dependent effects on the mRNA levels of on-target genes were measured by qRT-PCR (Supplementary Figure S9). As a result, almost no reductions of on-target activities were observed by the 2′-formamido modifications. Furthermore, to evaluate the impact of 2′-formamido modifications on off-target effects against expression of endogenous genes, microarray analyses were performed using siKIF23-430 (Entries 11–14) and siVIM-270 (Entries 1–3). To address the potential reduction in off-target activity, microarray experiments were conducted at 50 nM, a concentration higher than the IC50 of the endogenous on-target gene (Supplementary Figure S9). Unmodified siKIF23-430 and those modified with 2′-formamido modifications at positions 4, 5, and 6 (siKIF23-430_4U > FA, _5U > FA, and _6U > FA) were transfected into HeLa cells, respectively, and total RNA was purified for microarray analysis. Results were visualized using MA plots (Figure 4A-D) and cumulative distributions (Figure 4E–H). Red dots in MA plots represented the expression levels of the target gene, *KIF23*, which has two splice variants (NM_001367804 and NM_138555). The expression levels of both KIF23 transcript variants were clearly downregulated to approximately 25% by all siRNAs (Figure 4A-D, i). In the MA plots, dark blue dots represented the possible off-target transcripts with seed-matched (SM) sequence (s) in their 3′UTRs, while light blue dots corresponded to the other transcripts with no SM sequences. In the cumulative distributions (Figure 4E-H), the horizontal axis showed the log_2_ fold change ratios of the SM off-target and other transcripts, and the vertical axis showed the cumulative fraction. The shift of the cumulative distribution curve to the left means the reduction of the expression levels of the transcripts. The differences between the distributions of SM off-target transcripts and the other transcripts of each siRNA using the Wilcoxon rank sum test revealed that the unmodified siKIF23-430 exhibited a significant difference (*P* = 9.85 × 10^–10^), indicating that it has strong off-target effects. However, siKIF23-430_4U > FA showed no significant off-target effects (*P* = 0.051). Furthermore, siKIF23-430_5U > FA and _6U > FA also exhibited the tendencies of reducing off-target effects. These microarray data were verified by qRT-PCR (Supplementary Figure S11A–C). Likewise, the 2′-formamido modifications of siVIM-270 (Entries 1–3) exhibited decreased off-target effects without affecting the suppression levels of target VIM gene compared to the unmodified siVIM-270 (Supplementary Figure S10). These data were verified by qRT-PCR (Supplementary Figure S11D-F). We further confirmed the off-target effects of RNA-seq using siVIM-270. The results obtained are shown in MA plots (Figure 5A-C) and cumulative distributions (Figure 5D–F), and a similar trend to microarray was observed from RNA-seq analysis (Figure 5, Supplementary Figure S9). The unmodified and 2′-formamido modified siKIF23-430 and siVIM-270 showed strong effects on each target, *KIF23* and *VIM*, respectively. Although the unmodified siKIF23-430 and siVIM-270 showed apparent off-target effects, but other siRNAs with 2′-formamido modifications showed no or reduced off-target effects. The correlation coefficients (*r*) of the results of microarray and qRT-PCR were extremely high at *r* = 0.96 for siKF23-430 and *r* = 0.92 for siVIM-270. There results clearly indicated that the introduction of 2′-formamido modifications into the seed region is an effective strategy to mitigate off-target effects on endogenous genes.

**Figure 6.**
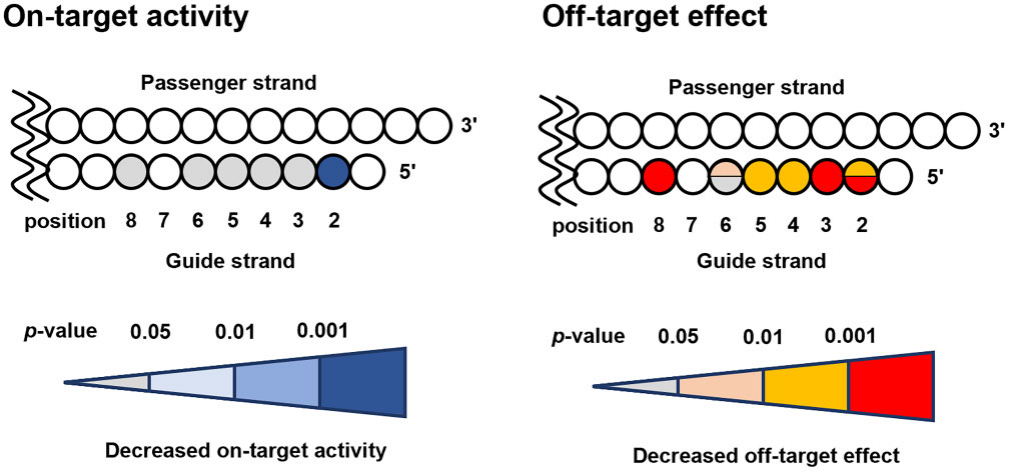
Heatmap of the effect of 2′-formamido modification for on- and off-target activity. The heatmap shows the *P*-value calculated by Two-Way ANOVA test from the reporter assay results for each modification position (Figure 3).

The above reporter assays, microarray analysis, and RNA-seq show that 2′-formamide modifications efficiently suppress off-target effects. Previous studies have shown that spacers, UNA, and GNA effectively suppress off-target effects (21,22,26). Spacers suppress off-target effects between positions 2 and 7 of the seed region, but only position 6 can maintain on-target activity (22). In general, spacers have difficulty maintaining on-target activity because of the lack of bases in the target recognition site. UNA and GNA, acyclic nucleosides with a base, can maintain on-target effects at position 7 in the seed region, maximally suppressing off-target effects (21,26). In contrast, the 2′-formamide modification can effectively suppress off-target effects while exhibiting on-target effects at a wide range of positions from 3 to 8 in the seed region (Figure 6). This difference is thought to be due to the fact that UNA and GNA have a smaller effect on RNA conformational changes in RISC and are more conformationally flexible due to the lack of ring structures in the sugar backbone. On the other hand, a 5-membered ring glycosyl backbone modified with 2′-formamide significantly alters the conformation of RNA in the seed region of RISC. The advantage of 2′-formamide modification is that it can be introduced anywhere in the seed region to suppress off-target effects while maintaining on-target activity (Figure 6).

## Conclusion

In this study, we synthesized 2′-formamidonucleoside phosphoramidites as novel sugar modifications for each of the four nucleobases. These were utilized to create RNA strands, which, upon evaluation, showed decreased stability in the double strands. However, when these modifications were introduced into the seed region of the siRNA, they maintained on-target effects while suppressing off-target activities in a sequence- and position-dependent manner. 2′-formamido modification, even with a single insertion in the seed region, significantly inhibited off-target activity. The novel 2′-formamido nucleic acid, in combination with existing modified nucleosides, shows promise in the development of optimized siRNA molecules that can effectively suppress off-target effects and improve *in vivo* stability. Our future plan involves integrating 2′-formamido nucleosides into fully chemically modified siRNAs with 2′-OMe, 2′-F, PS modifications, which are used as siRNA drugs, and examining their *in vivo* activity, stability, toxicity, and other factors.

## Supplementary Material

gkae741_Supplemental_File

## Data Availability

The data underlying this article are available in the article and online supplementary material. The atomic coordinates and experimental data of the RNA crystals were deposited in the Protein Data Bank under the accession number 8YNO. The microarray and RNA-seq data have been deposited in GEO under accession numbers GSE274299 and GSE274426, respectively.
