# Supplementary material for: Synthesis of 2′-formamidonucleoside phosphoramidites for suppressing the seed-based off-target effects of siRNAs
Source: Nucleic Acids Res. 2024 Sep 5;52(18):10754–74. doi: 10.1093/nar/gkae741 (PMC11472056; doi:10.1093/nar/gkae741)
Supplement: gkae741_Supplemental_File [file gkae741_supplemental_file.pdf]

Supporting Information

Synthesis of 2'-formamidonucleoside phosphoramidites for suppressing the seed-based off-target effects of siRNAs

Table of contents

Details of synthesis of 2'-formamidonucleoside phosphoramidites............................S2
Supplementary table, scheme, and figure..S5
NMR spectra of synthesized compounds..S21

Details of synthesis of 2'-formamidonucleoside phosphoramidites

The phosphoramidite of 2'-formamidouridine was synthesized as depicted in Scheme 1a. We employed 2,2'-*O*-cyclo-uridine (**1**), where the 2' carbon and the 2 position oxygen of the base form a cyclic structure, as the starting material, enabling the one-step introduction of an azide group at the 2'-α position. The commercially available compound **1** was dimethoxytritylated to yield compound **2**. Subsequently, the introduction of an azide at the 2' position in the treatment with NaN_3 provided compound **3**. Next, the azide group was converted to an amino group by Pd-catalyzed reduction, resulting in compound **4**(1). The resulting amino group was then condensed with formic acid to transform it into a formamido group, yielding compound **5**. Finally, compound **6**, the phosphoramidite form, was obtained using amidite reagent(2). Having successfully obtained the desired compound through the above reactions for the uridine derivative, we planned to introduce the formamido group into cytidine, adenosine, and guanosine derivatives by converting the azide group to an amino group and condensing it with formic acid.

The phosphoramidite of 2'-formamidocytidine was synthesized as outlined in Scheme 1b. Because cytidine cannot form a 2,2'-*O*-cyclo derivative, we used 2,2'-*O*-cyclo-uridine (**1**) as the starting material and decided to convert the uracil base to cytosine after introducing the formamido group. Initially, compound **7** was obtained by azidation at the 2' position using an azide ion as a nucleophile in an S_N2 reaction, followed by the protection of the 3' and 5' hydroxyl groups with TBS groups to yield compound **8**(3). Similar to the uridine derivative, the azide group was converted to a formamido group, resulting in compound **10**. Subsequently, the oxygen atom at the 4-position of the uracil base was transformed into a leaving group using 2,4,6-triisopropylbenzenesulfonyl chloride and then treated with aqueous ammonia to convert the uracil base to cytosine, yielding compound **11**(4). The exocyclic amino group of the cytosine base was then acetylated using acetic anhydride to obtain compound **12**(5), followed by deprotection of the 3' and 5' TBS groups with triethylamine trihydrofluoride to yield compound **13**(6). After protecting the 5' position with a DMTr group to obtain compound **14**, phosphoramidite form **15** was synthesized using an amidite reagent.

For purine bases, the 2'-α-OH group was converted to a β-OH group, followed by triflation to introduce an azide group. The phosphoramidite of 2'-formamidoadenosine was synthesized as shown in Scheme 1c. Initially, the 3' and 5' hydroxyl groups of commercially available adenosine (**16**) were protected with TIPDS to yield compound **17**(7). The 2'-α hydroxyl group was oxidized using chromium (VI) oxide to form compound **18** and then reduced with sodium borohydride to obtain compound **19** with a hydroxyl group at the 2'-β position(8). Although a yield of 49% was achieved on a small scale, it dropped to 29% when scaled up. This decrease in yield is thought to be due to precipitation of the target compound during column purification to remove chromium oxide when scaling up the

reaction. Although not attempted in the synthesis of the adenosine derivative, conducting the reaction used in the synthesis of the guanosine derivative, which is described later, might yield higher recovery rates when synthesized on a larger scale. Compound **20** was obtained by triflating the 2'-hydroxyl group using *N*-phenylbis(trifluoromethanesulfonimide)(9). Subsequently, the 2' position was azidated to yield compound **21**, followed by acetylation of the exocyclic amino group of adenosine using acetyl chloride to obtain compound **22**(10). From this point onward, the same reactions as those for the cytidine analog were performed to obtain the desired 2'-formamidoadenosine phosphoramidite **27**.

The phosphoramidite of 2'-formamidoguanosine was synthesized largely in a similar manner to the adenosine derivative, as shown in Scheme 1d. First, the exocyclic amino group of the commercially available guanosine (**28**) was protected with an isobutyryl group to yield compound **29**(11). Next, the 3' and 5' hydroxyl groups were protected with TIPDS to form compound **30**. In the reaction to introduce the hydroxyl group at the 2'-β position, an attempt to oxidize with chromic acid, as in the case of the adenosine derivative, was made. However, owing to the presence of unreacted compound **30** and a byproduct of isobutyryl group cleavage, the yield was below 15%. Therefore, the synthesis method was modified as follows. Initially, compound **31** was obtained by triflating the 2'-α hydroxyl group with trifluoromethanesulfonyl chloride. After an S_N2 reaction with trifluoroacetate as the nucleophile and subsequent hydrolysis of the trifluoroacetyl group, compound **32** with a hydroxyl group at the 2'-β position was obtained(10). Then, the 2'-β hydroxyl group was triflated using trifluoromethanesulfonyl chloride to yield **33**. In the triflation reaction, trifluoromethanesulfonyl chloride was used because it was difficult to separate *N*-phenylbis (trifluoromethanesulfonimide), which was used in the synthesis of the adenosine derivative, from the target product. Although this resulted in high purity of the target compound, the yield was still low at 28% for compound **31** and 20% for compound **33**, as shown in Scheme S1, because of the side products **31b** and **33b,** where the oxygen at the 6-position of the guanine base was triflated and then substituted by nucleophilic attack of DMAP. The side product emitted fluorescence at approximately 430 nm when excited at 340 nm (Figure S1). This phenomenon was attributed to the expansion of the conjugated system upon binding of DMAP to the guanine base(12). Subsequently, compound **34** was obtained by azidation at the 2'-α position using an azide ion as a nucleophile in an S_N2 reaction. From this point on, the same reactions as those for the cytidine analog were performed to obtain the desired 2'-formamidoguanosine phosphoramidite **39**.

References

1. Patra,A. and Richert,C. (2009) High fidelity base pairing at the 3'-terminus. *J. Am. Chem. Soc.*, **131**, 12671-12681.

2. Chapuis,H., Bui,L., Bestel,I. and Barthélémy,P. (2008) 2′-Lipid-modified oligonucleotides via a 'Staudinger-Vilarrasa' reaction. *Tetrahedron Lett.*, **49**, 6838–6840.
3. Seamon,K.J., Hansen,E.C., Kadina,A.P., Kashemirov,B.A., McKenna,C.E., Bumpus,N.N. and Stivers,J.T. (2014) Small molecule inhibition of SAMHD1 dNTPase by tetramer destabilization. *J. Am. Chem. Soc.*, **136**, 9822–9825.
4. Ogawa,A., Tanaka,M., Sasaki,T. and Matsuda,A. (1998) Nucleosides and nucleotides. 180. Synthesis and antitumor activity of nucleosides that have a hydroxylamino group instead of a hydroxyl group at the 2'- or 3'-position of the sugar moiety. *J. Med. Chem.*, **41**, 5094–5107.
5. Fauster,K., Hartl,M., Santner,T., Aigner,M., Kreutz,C., Bister,K., Ennifar,E. and Micura,R. (2012) 2'-azido RNA, a versatile tool for chemical biology: Synthesis, X-ray structure, siRNA applications, click labeling. *ACS Chem. Biol.*, **7**, 581-589.
6. Yamada,H., Kitauchi,Y., Tanabe,K., Ito,T. and Nishimoto,S.I. (2011) Anthraquinone-sensitized photooxidation of 5-methylcytosine in DNA leading to piperidine-induced efficient strand cleavage. *Chem. Eur. J.*, **17**, 2225–2235.
7. Zhong,M. and Strobel,S.A. (2006) Synthesis of the ribosomal P-site substrate CCA-pcb. *Org. Lett.*, **8**, 55–58.
8. Marriott,J.H., Mottahedeh,M. and Reese,C.B. (1991) Synthesis of 2'-thioadenosine. *Carbohydr. Res.*, **216**, 257–269.
9. Wnuk,S.F., Lewandowska,E., Companioni,D.R., Garcia,P.I. and Secrist,J.A. (2004) Synthesis and cytotoxicity of 9-(2-deoxy-2-alkyldithio-β-D-arabinofuranosyl)purine nucleosides which are stable precursors to potential mechanistic probes of ribonucleotide reductases. *Org. Biomol. Chem.*, **2**, 120–126.
10. Puffer,B., Moroder,H., Aigner,M. and Micura,R. (2008) 2'-Methylseleno-modified oligoribonucleotides for X-ray crystallography synthesized by the ACE RNA solid-phase approach. *Nucleic Acids Res.*, **36**, 970-983.
11. Eisenfu¨hr,A.E., Arora,P.S., Sengle,G., Takaoka,L.R., Nowick,J.S. and Famulok,M. (2003) A Ribozyme with Michaelase Activity: Synthesis of the Substrate Precursors. *Bioorg. Med. Chem.*, **11**, 235-249.
12. Wenska,G., Skalski,B., Paszyc,S., Wnuk,S. and A,I.W. (1994) Fluorescent Nucleoside Derivatives: Luminescence Study of 4-Dimethylaminopyridinium Chloride Derived from Guanosine. *J. Fluoresc.*, **4**, 283-286.

Table S1. Sequences and molecular weight of synthesized RNA

Name	Sequence[a]	Calculated M.W.	Observed M.W.	Isolation yield
RNA_1	5'-ACUGCUACGAU-3'	3450.1266	3452.878	12.9%
RNA_2	5'-ACUGC**U**^{f}ACGAU-3'	3477.1526	3476.363	16.8%
RNA_3	5'-AUCGUAGCAGU-3'	3490.1516	3497.095	14.7%
RNA_4	5'-AUCGU**A**^{f}GCAGU-3'	3517.1776	3520.098	9.3%
RNA_5	5'-GUCAUCGUAGC-3'	3466.1256	3468.738	6.1%
RNA_6	5'-GUCAU**C**^{f}GUAGC-3'	3493.1516	3495.675	4.0%
RNA_7	5'-GCUACGAUGAC-3'	3489.1676	3491.485	9.7%
RNA_8	5'-GCUAC**G**^{f}AUGAC-3'	3516.1936	3517.756	5.2%
RNA_9	5'-AUCGUUGCAGU-3'	3467.1096	3471.725	7.6%
RNA_10	5'-AUCGUGGCAGU-3'	3506.1506	3510.158	10.6%
RNA_11	5'-AUCGUCGCAGU-3'	3466.1256	3472.798	12.8%
RNA_12	5'-GGAC**U**^{Br}CGAG**U**^{f}CC-3'	3916.2714	3918.622	24.8%
RNA_13	5'-UU**G**^{f}AACUCGGUGUUGAUGGCG-3'	6773.0662	6772.463	14.4%
RNA_14	5'-UUGAACU**C**^{f}GGUGUUGAUGGCG-3'	6773.0662	6774.720	17.5%
RNA_15	5'-A**A**^{f}GUCGACUUGGAUUCACCUU-3'	6661.0182	6661.840	4.4%
RNA_16	5'-AAG**U**^{f}CGACUUGGAUUCACCUU-3'	6661.0182	6663.679	25.2%
RNA_17	5'-UCG**U**^{f}UUAGCUUGAAAUGACCC-3'	6661.0182	6665.582	7.8%
RNA_18	5'-UCGU**U**^{f}UAGCUUGAAAUGACCC-3'	6661.0182	6661.997	11.6%
RNA_19	5'-UCGUU**U**^{f}AGCUUGAAAUGACCC-3'	6661.0182	6661.973	15.9%
RNA_20	5'-U**A**^{f}UGAUGAUCCCAACCCGCUU-3'	6620.0092	6620.810	6.5%
RNA_21	5'-UA**U**^{f}GAUGAUCCCAACCCGCUU-3'	6620.0092	6620.003	28.4%
RNA_22	5'-UAUGA**U**^{f}GAUCCCAACCCGCUU-3'	6620.0092	6619.946	29.2%

[a] **N**^{f} is 2'-formamidonucleoside. **U**^{Br} is 5-bromo-uridine.

Table S2. Crystallization conditions

	conc.
RNA solution	
RNA	1 mM
Crystallization solution	
Sodium cacodylate (pH = 7.0)	50 mM
Spermine tetrahydrochloride	10 mM
Lithium chloride	300 mM
2-Methyl-2,4-pentanediol	10%
Reservoir solution	
2-Methyl-2,4-pentanediol	40%

Table S3. Crystal data, and statistics of data collection and structure refinement

Crystal data	
Space group	$P2_1$
Unit cell (Å)	a = 27.6, b = 43.1, c = 28.0, β = 109.5
Z [a]	1
Data collection	
Beamline	BL-17A of Photon Factory
Wavelength (Å)	0.98
Resolution (Å)	26.4-1.3
of the outer shell (Å)	1.33-1.30
Unique reflections	15146
Completeness (%)	98.3
in the outer shell (%)	84.2
R_{merge} (%)	5.9
in the outer shell (%)	14.6
Redundancy	6.5
in the outer shell	5.1
Structure refinement	
Resolution range (Å)	26.4-1.3
Used reflections	15137
R-factor (%)	15.1
R_{free} (%)	17.3
R.m.s.d. bond length (Å)	0.006
R.m.s.d. bond angles (°)	1.1

[a] Number of duplexes in the asymmetric unit.

Table S4. Primer Sequences

Primer name[a]	Sequence
GAPDH_F	5'-TGCACCACCAACTGCTTAG-3'
GAPDH_R	5'-AGAGGCAGGGATGATGTTC-3'
KIF23_F	5'-AGGGCTGCTTCCTCGTTGTT-3'
KIF23_R	5'-ACTCTGGATCTACTTGTCGTTTGCT-3'
SPIN1_F	5'-CCTCCCTGATAGAGTTGCGACA-3'
SPIN1_R	5'-AGGTGCACGTGCTAAGACCA-3'
PTP4A2_F	5'-TTGCAGGATTGGGAAGGGCA-3'
PTP4A2_R	5'-GCAGCTGTTTGGAATTGAACGC-3'
SRSF7_F	5'-GAGCAGGGGACGAAGGTCAA-3'
SRSF7_R	5'-GACCTCGACGGGGATTGGAA-3'
SURF4_F	5'-GTCAAGGTTGGTTGGCTGAT-3'
SURF4_R	5'-GCCAGGAGAAACAGGAACAC-3'
VIM_F	5'-CAGGACTCGGTGGACTTCTC-3'
VIM_R	5'-GTCGATGTAGTTGGCGAAGC-3'
VAPA_F	5'-TTCAGGAAATGCCAAGAGGT-3'
VAPA_R	5'-TCAACAACTGCCTCACAAGG-3'
MTPN_F	5'-TAGGTGCAGTGTGTGGAAGC-3'
MTPN_R	5'-TGCATGGAAGAAAACAGCAG-3'
PTPRF_F	5'-CTGGTTTGCAGCTGTTTTCA-3'
PTPRF_R	5'-CCTCAGCAAGCTGGGATAAT-3'

[a] F = forward primer, R = reverse primer

Table S5. T_m value of siRNAs with modification

Sample name	Sequence[a]	T_m (°C)[b]	ΔT_m (°C)
siVIM-270	5' - CCAUCAACACCGAGUUCAAGA - 3'<br>3' - GCGGUAGUUGUGGCUCAAGUU - 5'	64.7±0.1	-
siVIM-270_3G>FA	5' - CCAUCAACACCGAGUUCAAGA - 3'<br>3' - GCGGUAGUUGUGGCUCAA**G**^{f}UU - 5'	62.5±0.1	-2.2
siVIM-270_8C>FA	5' - CCAUCAACACCGAGUUCAAGA - 3'<br>3' - GCGGUAGUUGUGG**C**^{f}UCAAGUU - 5'	61.1±0.1	-3.6
siCLTC-2416	5' - GGUGAAUCCAAGUCGACUUCC - 3'<br>3' - UUCCACUUAGGUUCAGCUGAA - 5'	66.4±0.1	-
siCLTC-2416_2A>FA	5' - GGUGAAUCCAAGUCGACUUCC - 3'<br>3' - UUCCACUUAGGUUCAGCUG**A**^{f}A - 5'	65.6±0.1	-0.8
siCLTC-2416_4U>FA	5' - GGUGAAUCCAAGUCGACUUCC - 3'<br>3' - UUCCACUUAGGUUCAGC**U**^{f}GAA - 5'	62.2±0.3	-4.2
siKIF23-430	5' - GUCAUUUCAAGCUAAACGAUA - 3'<br>3' - CCCAGUAAAGUUCGAUUUGCU - 5'	56.6±0.0	-
siKIF23-430_4U>FA	5' - GUCAUUUCAAGCUAAACGAUA - 3'<br>3' - CCCAGUAAAGUUCGAUU**U**^{f}GCU - 5'	52.8±0.1	-3.8
siKIF23-430_5U>FA	5' - GUCAUUUCAAGCUAAACGAUA - 3'<br>3' - CCCAGUAAAGUUCGAU**U**^{f}UGCU - 5'	52.7±0.1	-3.9
siKIF23-430_6U>FA	5' - GUCAUUUCAAGCUAAACGAUA - 3'<br>3' - CCCAGUAAAGUUCGA**U**^{f}UUGCU - 5'	52.7±0.0	-3.9
siMC4R-490	5' - GCGGGUUGGGAUCAUCAUAAG - 3'<br>3' - UUCGCCCAACCCUAGUAGUAU - 5'	71.8±0.0	-
siMC4R-490_2A>FA	5' - GCGGGUUGGGAUCAUCAUAAG - 3'<br>3' - UUCGCCCAACCCUAGUAGU**A**^{f}U - 5'	71.4±0.1	-0.4
siMC4R-490_3U>FA	5' - GCGGGUUGGGAUCAUCAUAAG - 3'<br>3' - UUCGCCCAACCCUAGUAG**U**^{f}AU - 5'	70.5±0.0	-1.3
siMC4R-490_6U>FA	5' - GCGGGUUGGGAUCAUCAUAAG - 3'<br>3' - UUCGCCCAACCCUAG**U**^{f}AGUAU - 5'	68.1±0.1	-3.7

[a] **X**^{f}: 2'-formamidonucleoside, [b] Three measurements were taken, and the mean and standard error of the measurements were shown.

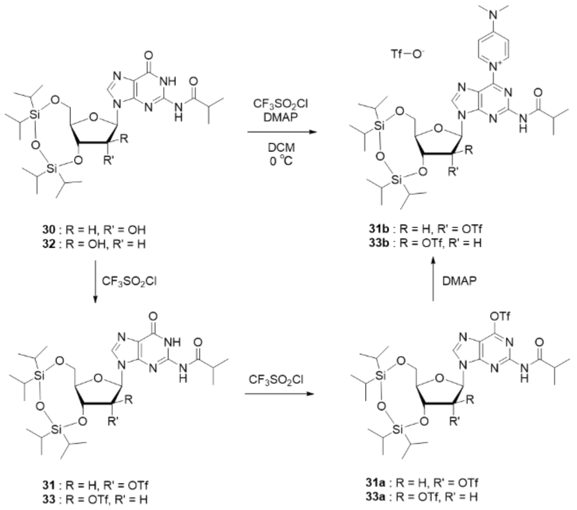

Scheme S1. Production of byproduct DMAP reacted with guanine base.

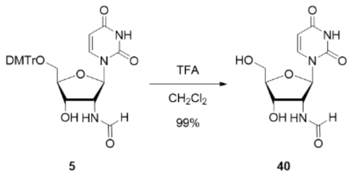

Scheme S2. Synthesis of 2′-formamidouridine

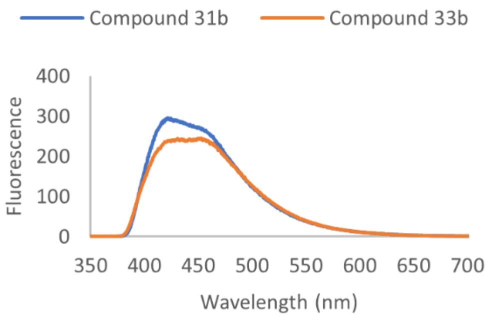

Figure S1. Fluorescence of compounds 31b and 33b. The measurement conditions were an excitation wavelength of 340 nm and sample concentration of 40 mg/mL in MeOH.

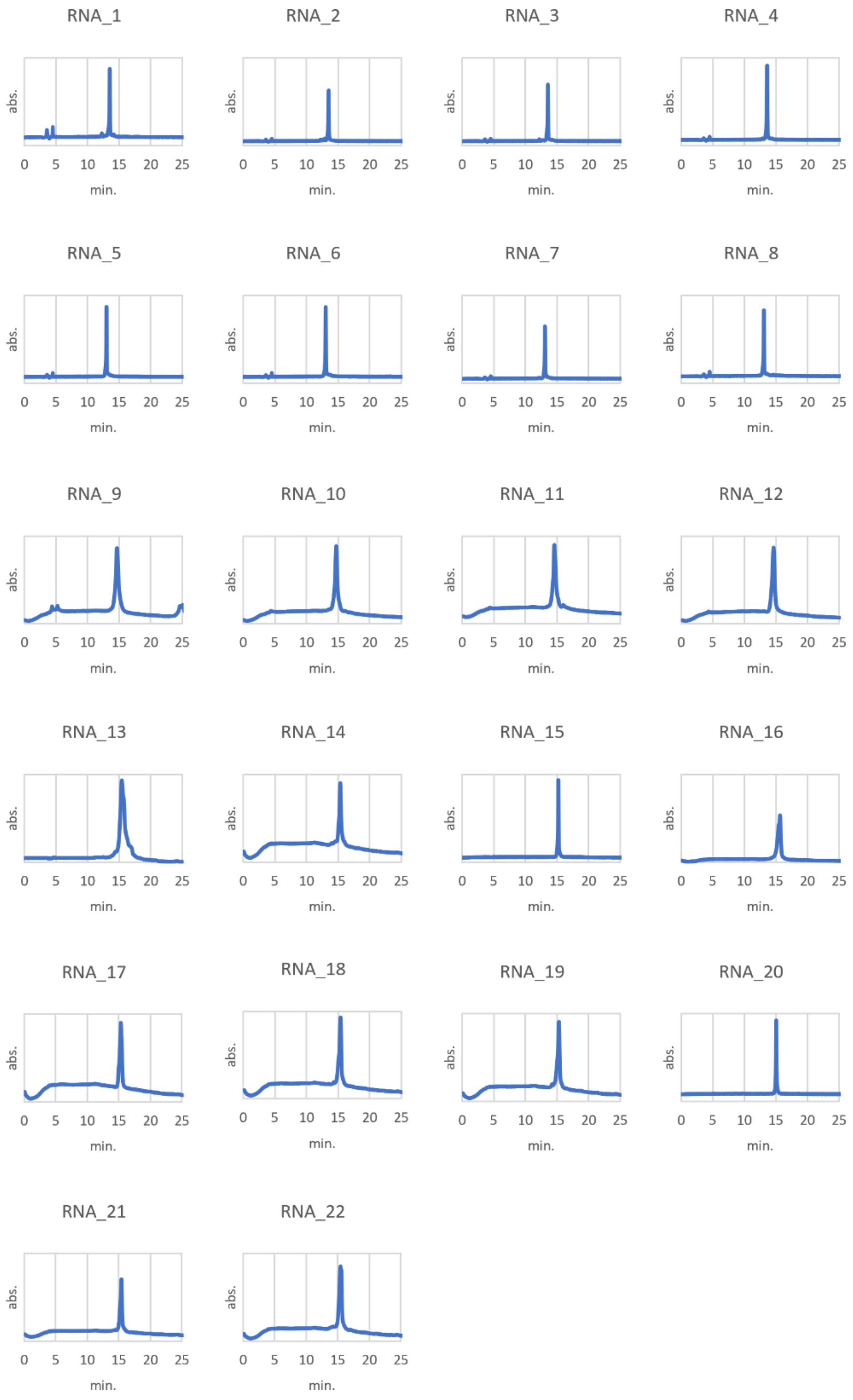

Figure S2. The HPLC chromatograms of synthesized RNA. The condition of HPLC analysis is following. communications bus module: CBM-20A (Shimadzu), liquid chromatograph: LC-20AD (Shimadzu), Autosampler: SIL-20AC (Shimadzu), column oven: CTO-20AC (Shimadzu), degassing unit: DGU-20A5R (Shimadzu), valve unit: FCV-11AL (Shimadzu), photo diode array detector: SPD-M40 (Shimadzu), column: YMC Hydrosphere C18 (250 x 4.6 mmI.D., S-5 µm, 12 nm), elution A: 50 mM TEAA (pH 7.0) with 5% ACN, elution B: ACN, gradient: 0-30%B/0-20 min, 80%B/20-25 min, column temperature: 40 °C, flow rate: 1 mL/min.

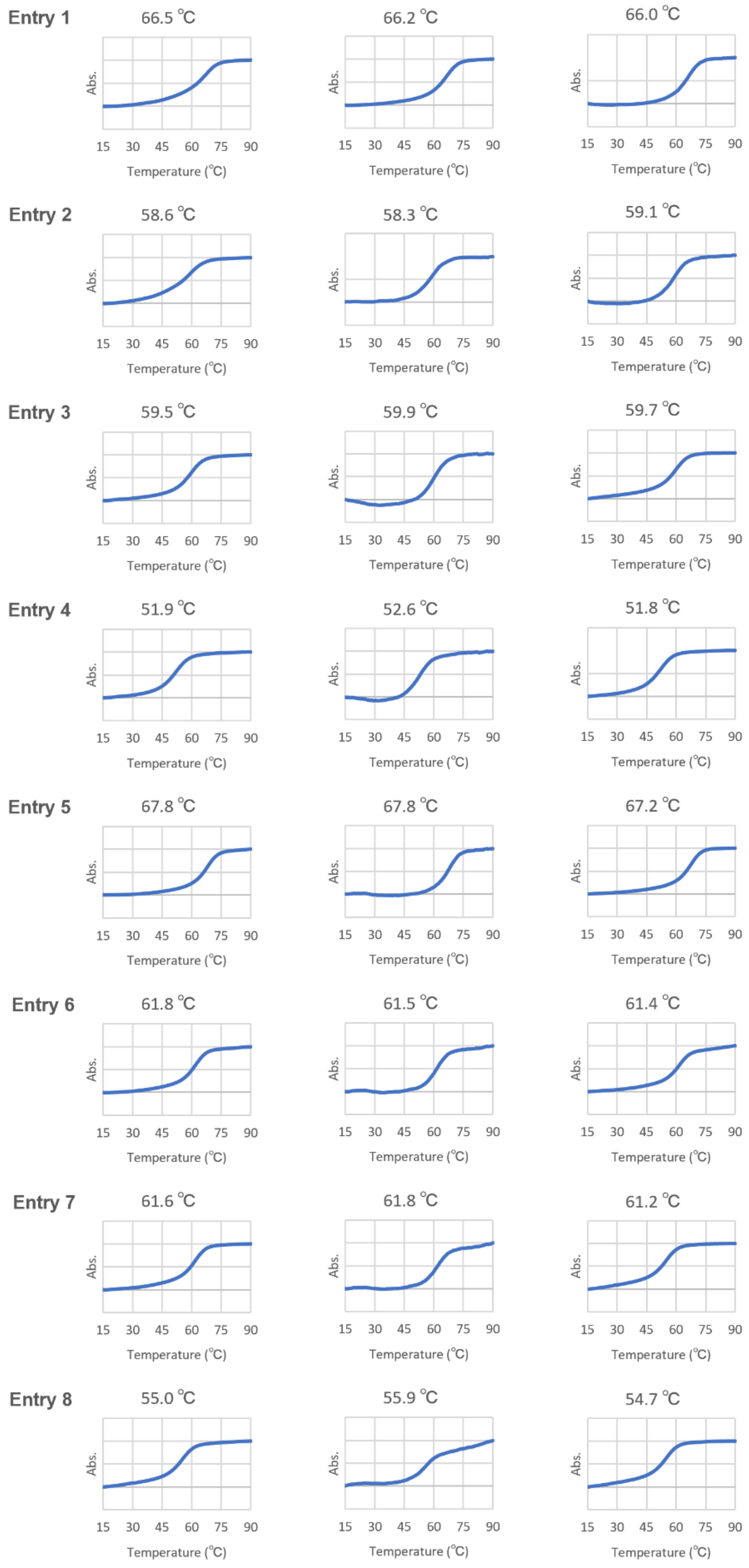

Figure S3. The melting curves of double strand RNA which has 1 or 2 2'-formamidonucleoside in the center of 11 mer RNA. Measurement conditions: 3 μM each strand of oligo RNA, 10 mM phosphate buffer (pH 7), 1 M NaCl, 15-90 °C (0.5 °C/min).

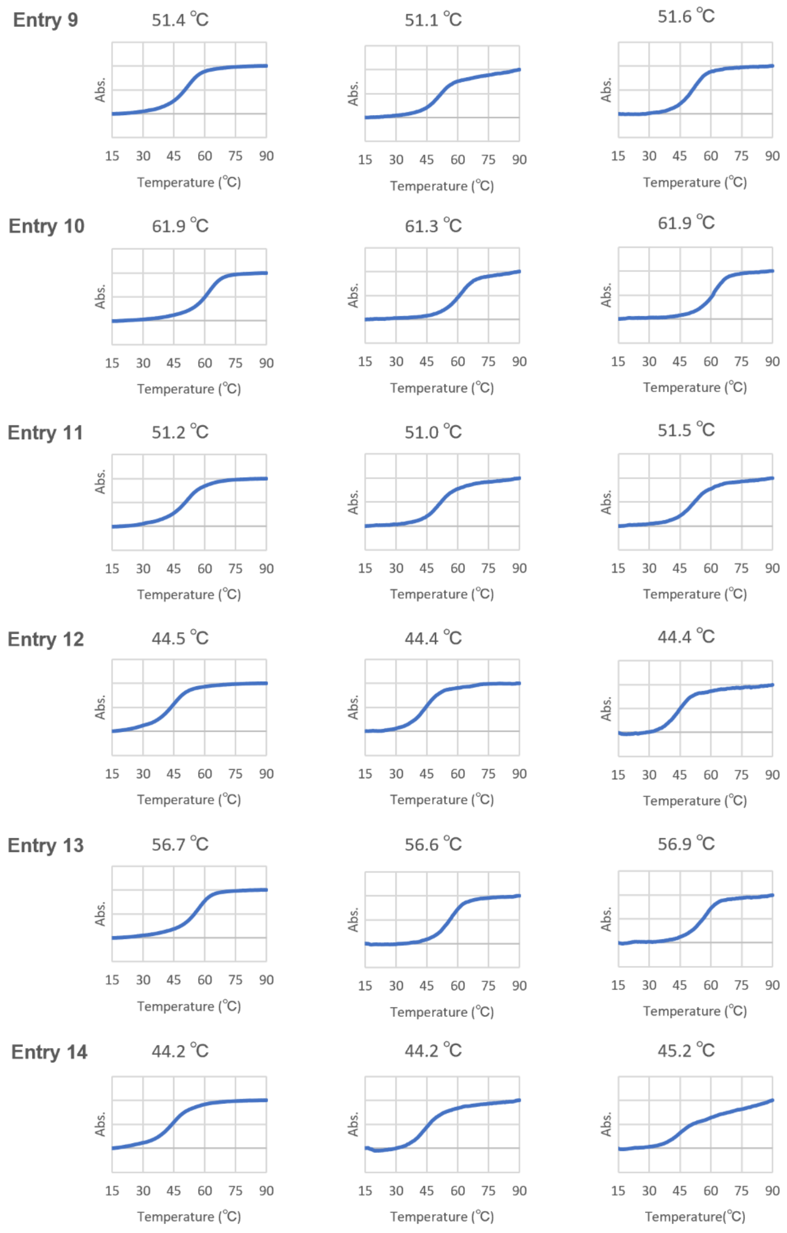

Figure S4. The melting curves of double strand RNA which has a mismatch base pairing with uridine or 2'-formamidouridine at the center of 11 mer RNA. Measurement conditions: 3 μM each strand of oligo RNA, 10 mM phosphate buffer (pH 7), 1 M NaCl, 15-90 °C (0.5 °C/min).

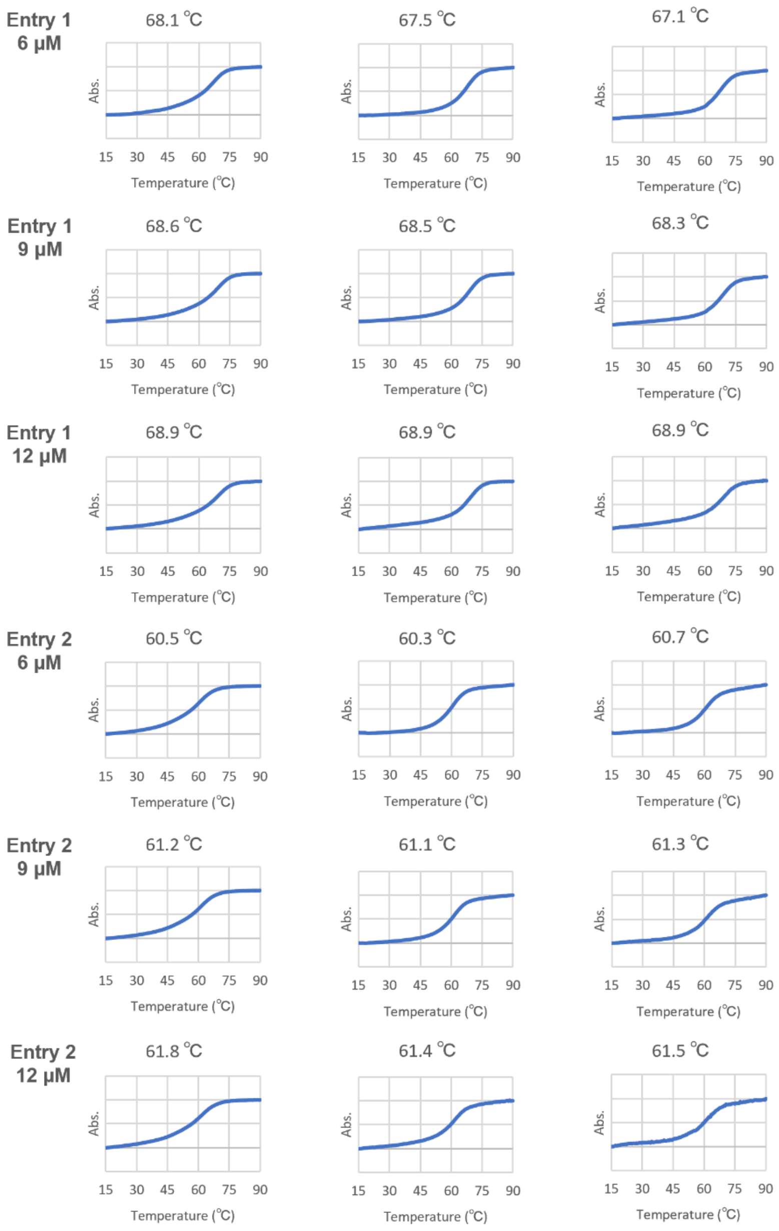

Figure S5. The melting curves of different concentration double strand RNA for calculation of thermodynamic parameters. Measurement conditions: 3, 6, 9, or 12 μM each strand of oligo RNA, 10 mM phosphate buffer (pH 7), 1 M NaCl, 15-90 °C (0.5 °C/min).

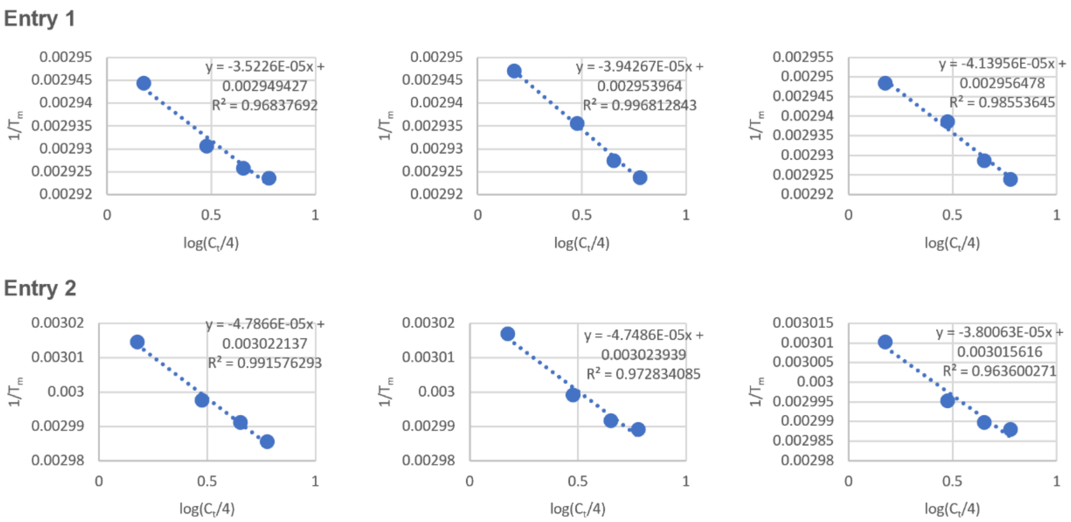

Figure S6. The results of the $1/T_m$ versus log (C_t/4) plot. T_m value were measured at 3, 6, 9, or 12 µM each strand of oligo RNA, and the results were used for plot.

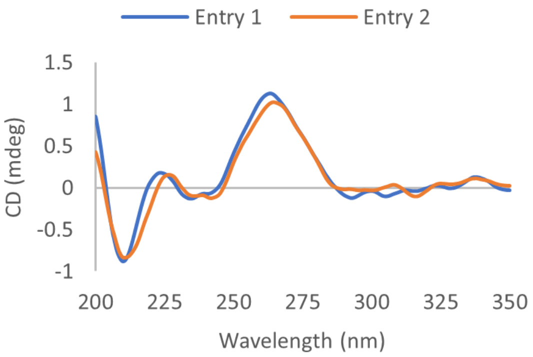

Figure S7. CD spectra of double-stranded strand RNA. Spectra of natural RNA (blue line) and RNA with 2'-formamidonucleoside (orange line).

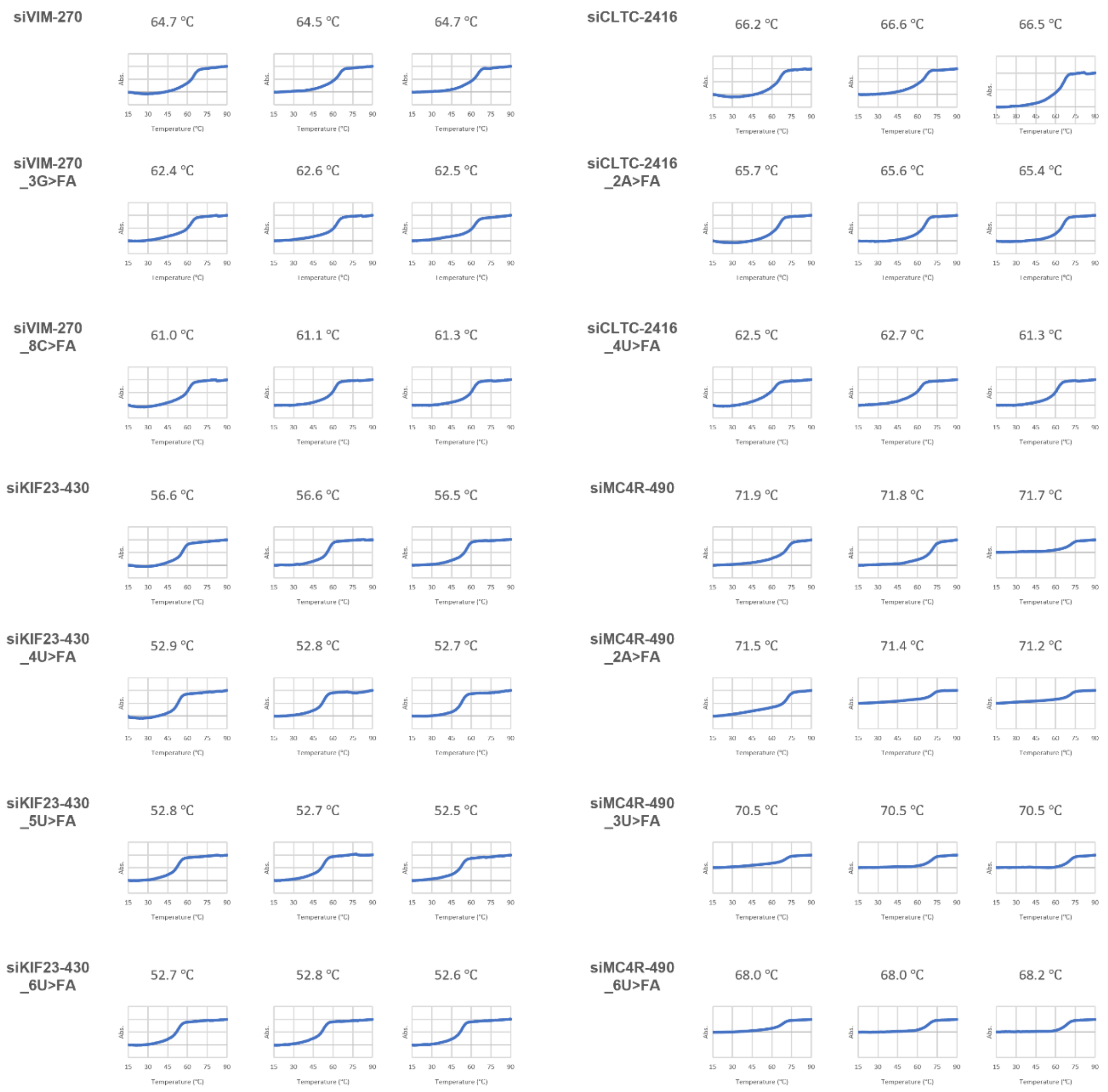

Figure S8. The melting curves of double strand siRNA which has a 2'-formamidonucleoside in the seed region of guide strand. Measurement conditions: 0.3 µM each strand of siRNA, 10 mM phosphate buffer (pH 7), 25 mM NaCl, 15-90 °C (0.5 °C/min).

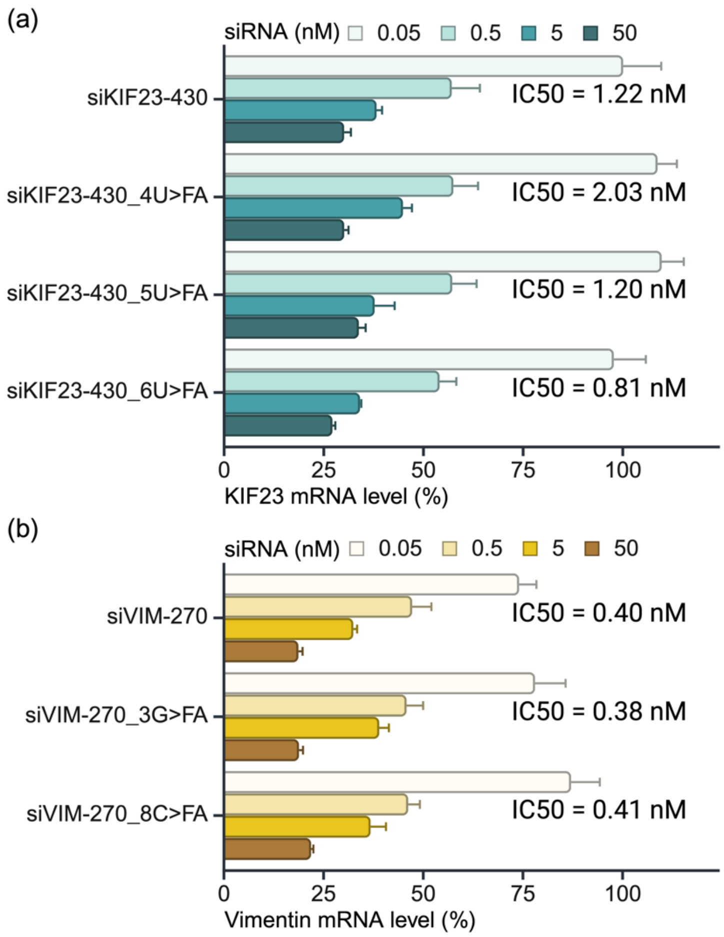

Figure S9. Dose-dependent effects of siRNAs on endogenous on-target genes. (a) Expression levels of KIF23 mRNAs by siKIF23-430 and its 2′-FA modified siKIF23-430s measured by qRT-PCR. (b) Expression levels of siVIM-270 and 2′-FA modified measured by qRT-PCR. The half maximal inhibitory concentrations (IC50s) of siRNAs were estimated using the following linear model. Expression level of each gene was calculated by comparing that in the control sample transfected with the same concentrations of siCont. IC50=10^(LOG10(A/B)*(50-C)/(D-C)+LOG10(B)), A: Concentration when inhibitory efficiency is lower than 50%, B: Concentration when inhibitory efficiency is higher than 50%, C: Inhibitory efficiency in B, D: Inhibitory efficiency in A.

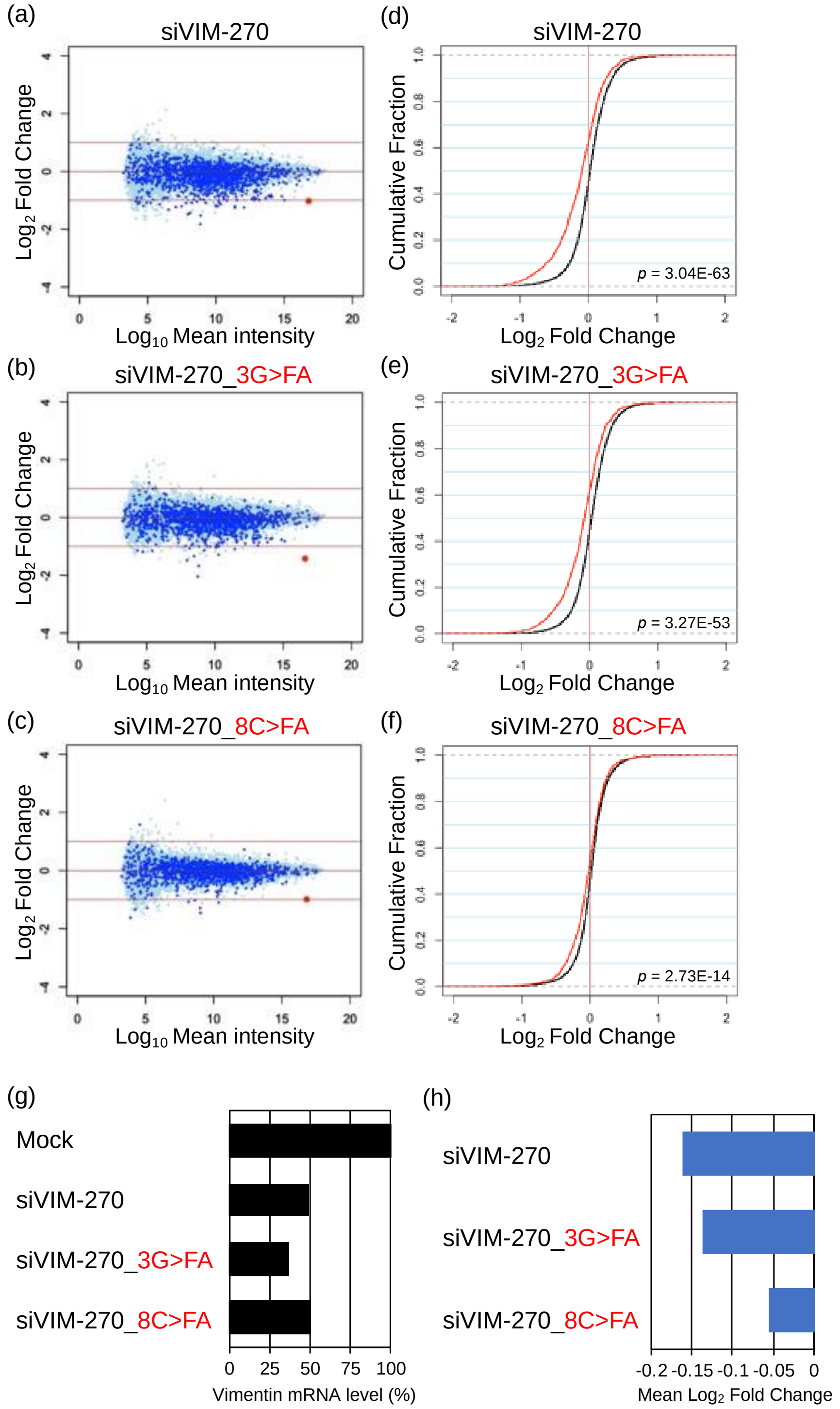

Figure S10. Microarray profiling of on-target and off-target effects of siVIM-270 with 2'-formamido modifications. (a-c) MA plots showing $\log_2$ fold changes (vertical lines) and average $\log_{10}$ signal intensities (horizontal lines) of the expression levels of transcripts from the cells transfected with siVIM-270 and its variants versus those from mock transfected cells. Dark blue dots represent 1377 transcripts with siVIM-270 SM sequence (off-target transcripts) in their 3′ UTRs, light blue dots represent 14376 other transcripts without SM sequence (not off-target transcripts),. (d-f) Cumulative distributions of transcripts showing $\log_2$ fold changes (horizontal lines) and

cumulative fractions (vertical lines). Red and black lines show the results for off-target and non-off-target transcripts, respectively. P-values were obtained using Wilcoxon rank-sum test. (g) *VIM* mRNA levels calculated by signal intensities compared to mock. (h) Seed-dependent off-target effects shown by mean $\log_2$ fold change for unmodified siVIM-270 and 2′-formamido-modified siVIM-270s at positions 3 or 8.

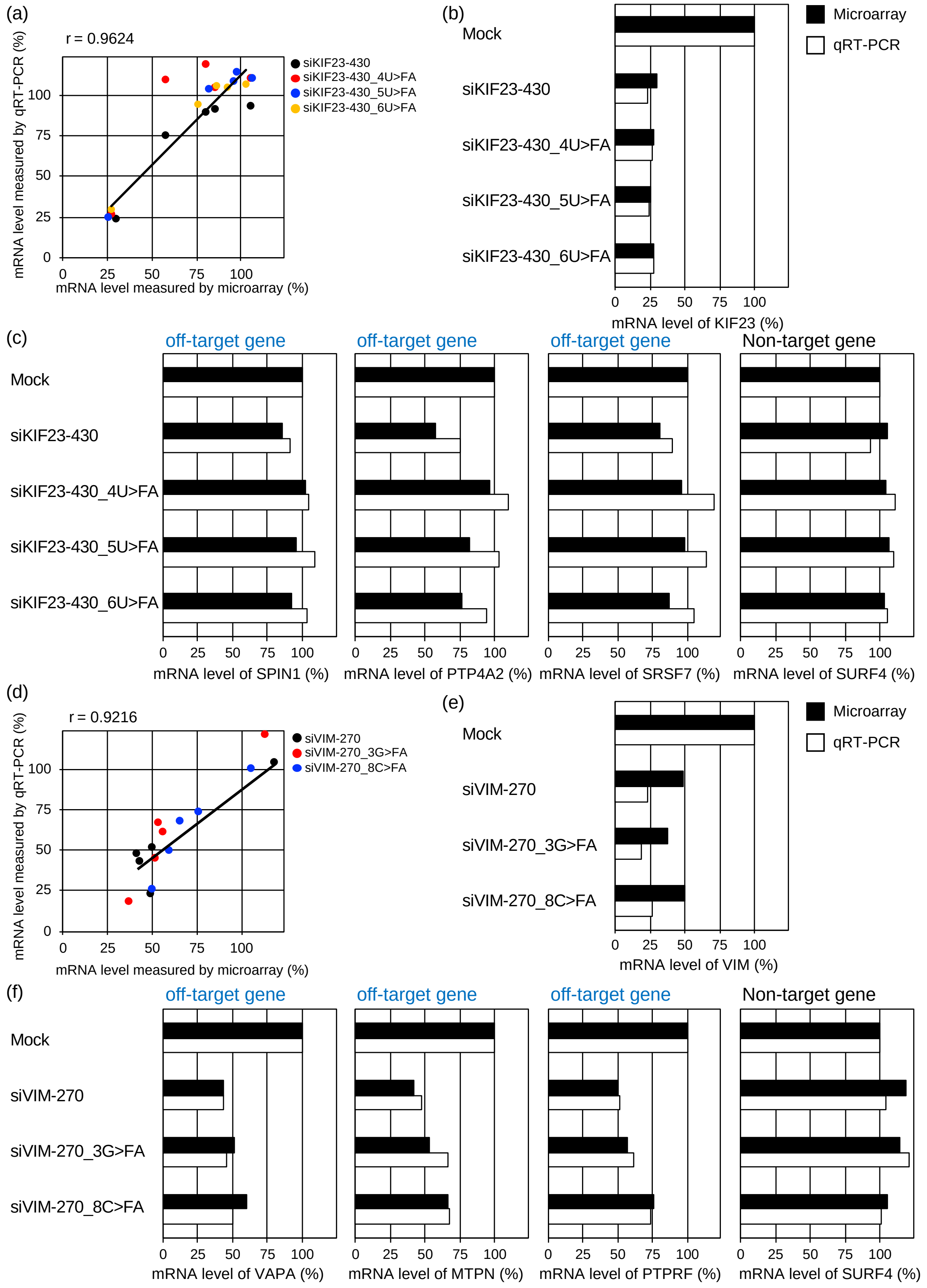

Figure S11. Comparison of gene expression levels analyzed by microarray and qRT-PCR. (a) Comparative analysis of typical gene expression levels of target, off-target and non-target genes of siKIF23-430. As off-target genes with SM sequences in their 3′ UTRs, *Spindlin 1* [SPIN1], *Protein*

Tyrosine Phosphatase 4A2 [PTP4A2], *Serine And Arginine Rich Splicing Factor 7* [SRSF7]) were selected, and as a non-target gene, *Surfeit 4* [SURF4] was used. Black, red, blue, and yellow dots indicating data from unmodified siKIF23-430, siKIF23-430_4U>FA, siKIF23-430_5U>FA, and siKIF23-430_6U>FA, respectively. Their correlation coefficient was 0.9624. (b-c) Detailed comparisons of microarray and qRT-PCR data of off-target and non-target genes. (d) Same analysis of siVIM-270. Three off-target genes (*vesicle associated membrane protein-associated protein A* [VAPA], *myotrophin* [MTPN], *protein tyrosine phosphatase receptor type F* [PTPRF]) and non-target SURF4 were used for comparison. Black, red, and blue dots indicating data from unmodified siVIM-270, siVIM-270-430_3G>FA, and siVIM-270-430_8C>FA, respectively. Their correlation coefficient was 0.9216. (e-f) Detailed comparisons of microarray and qRT-PCR data of off-target and non-target genes.

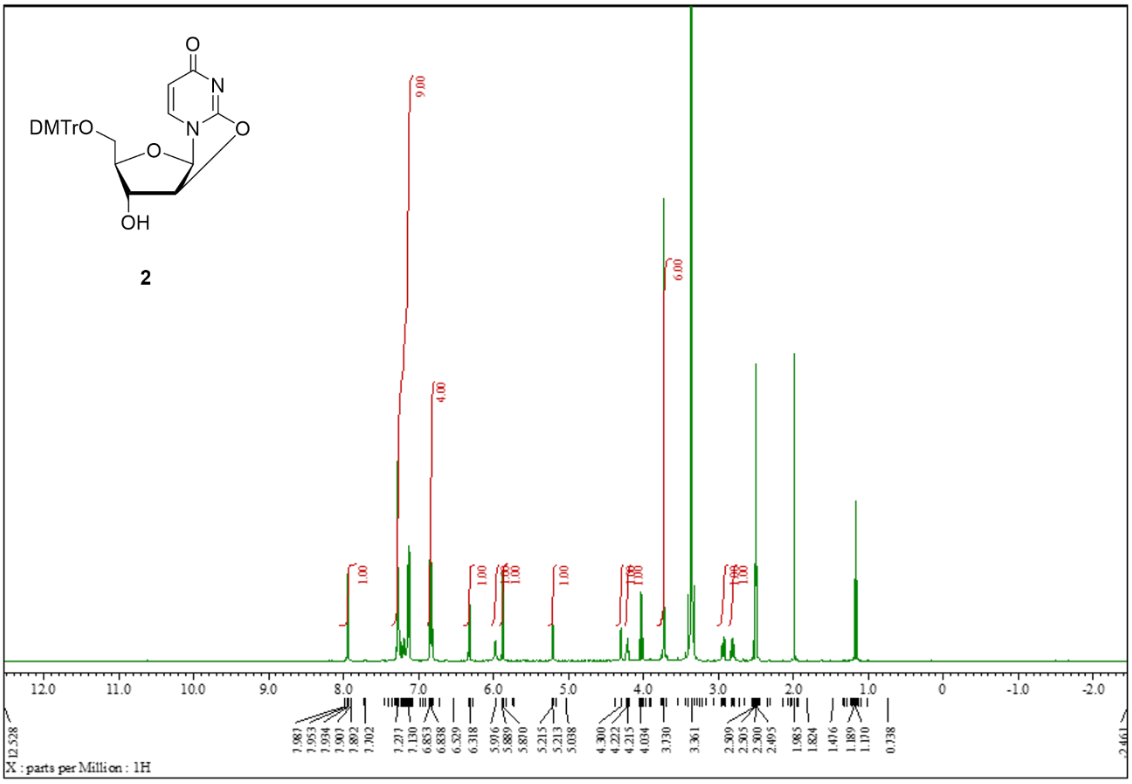

^{1}H-NMR (400 MHz, DMSO-d_6) of Compound **2**

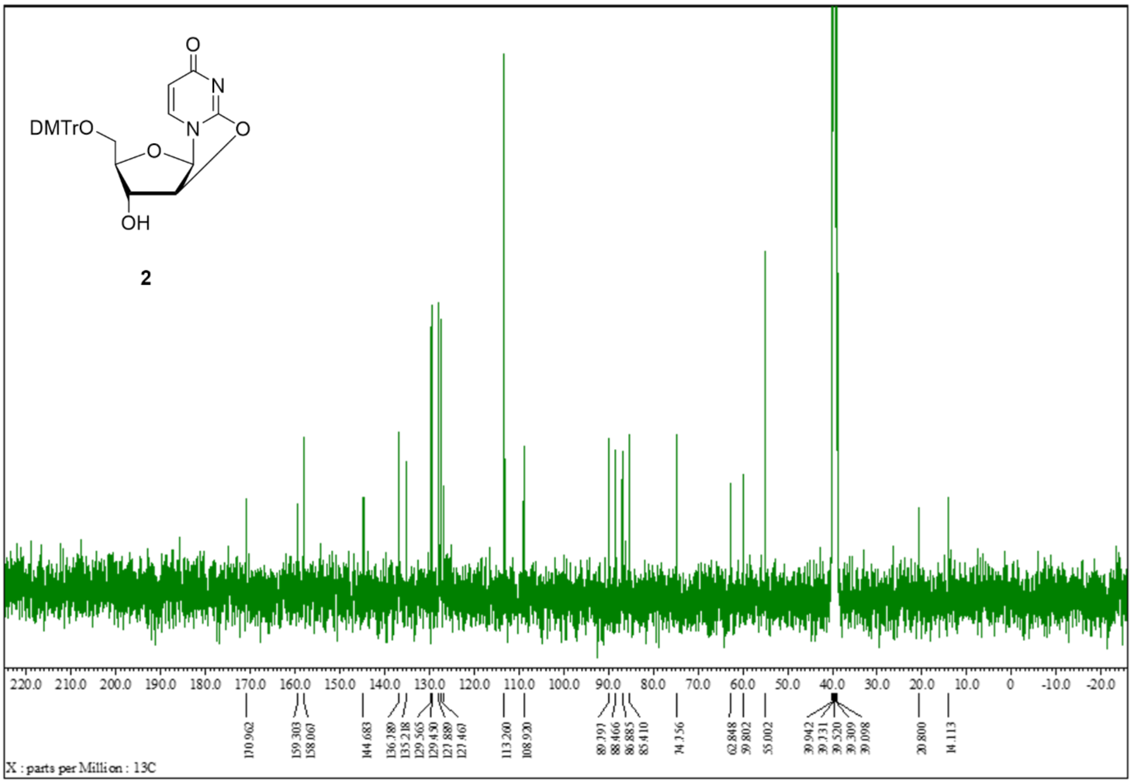

^{13}C-NMR (100 MHz, DMSO-d_6) of Compound **2**

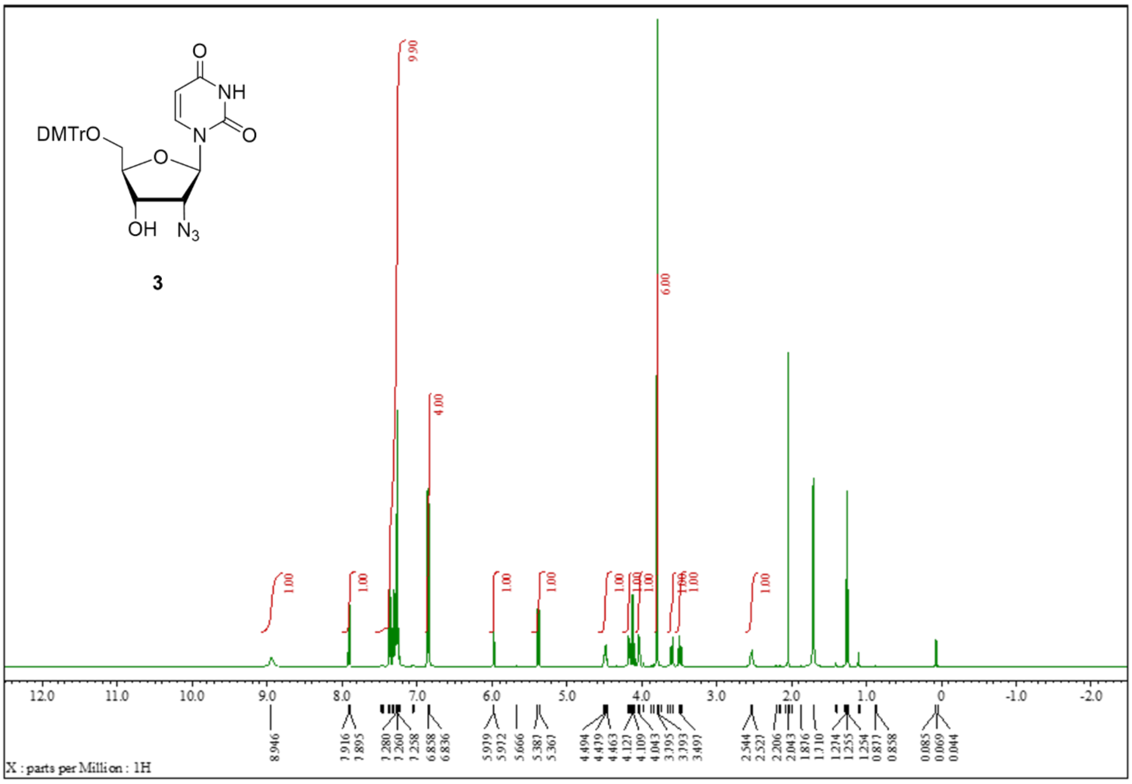

^{1}H-NMR (400 MHz, $CDCl_3$) of Compound **3**

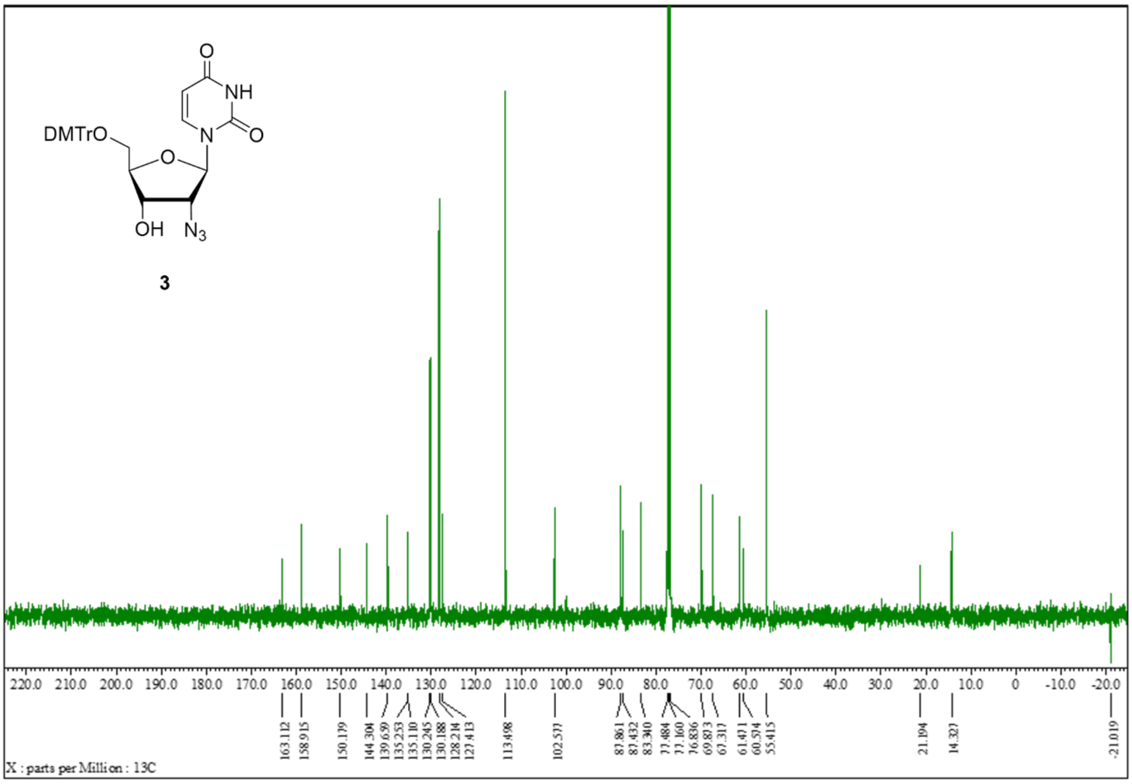

^{13}C-NMR (100 MHz, $CDCl_3$) of Compound **3**

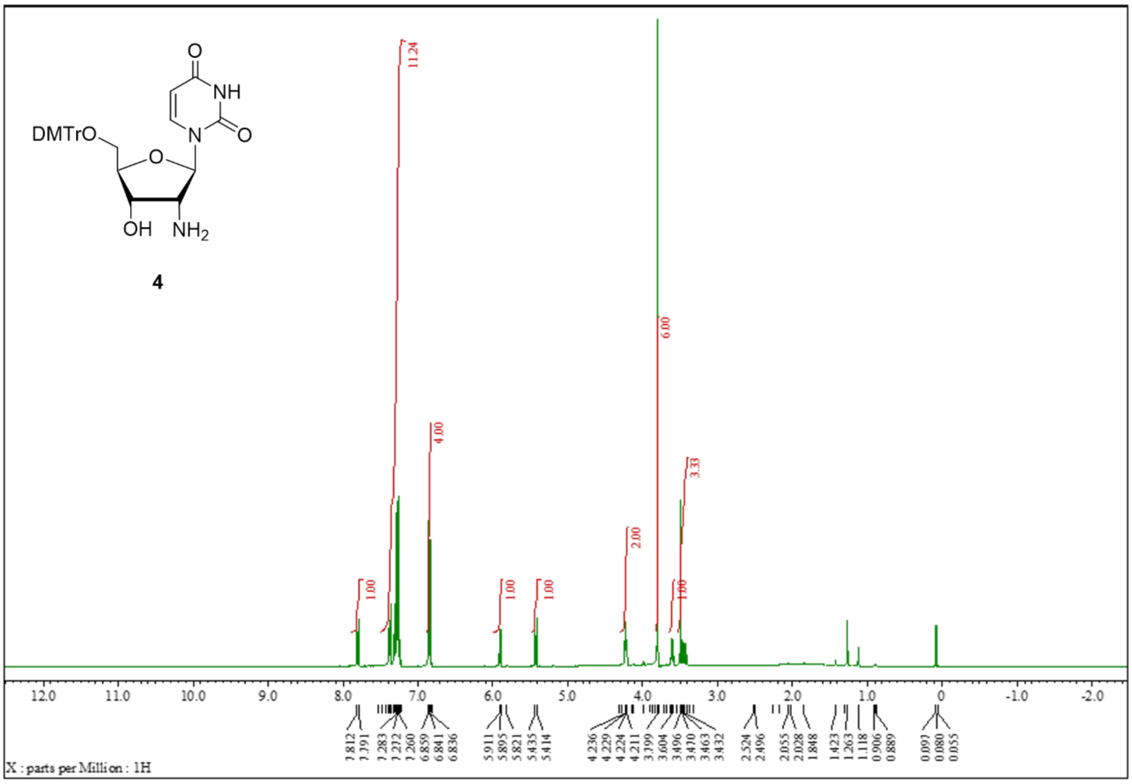

^{1}H-NMR (400 MHz, $CDCl_3$) of Compound **4**

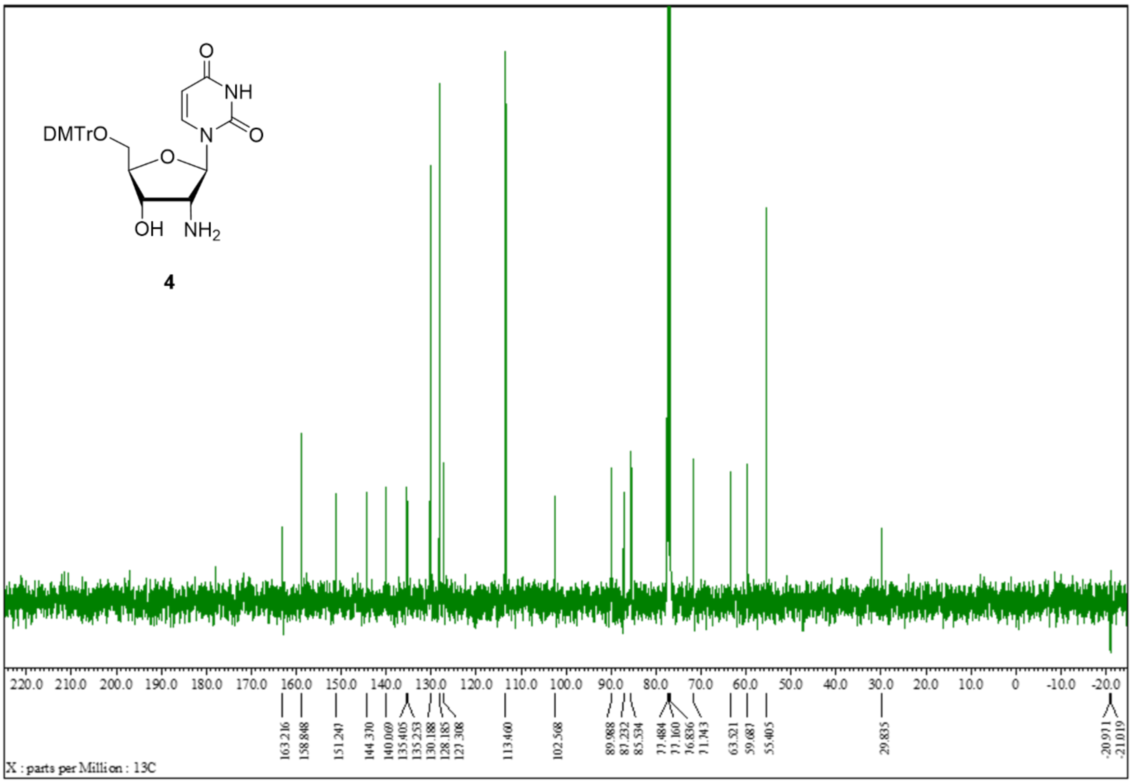

^{13}C-NMR (100 MHz, $CDCl_3$) of Compound **4**

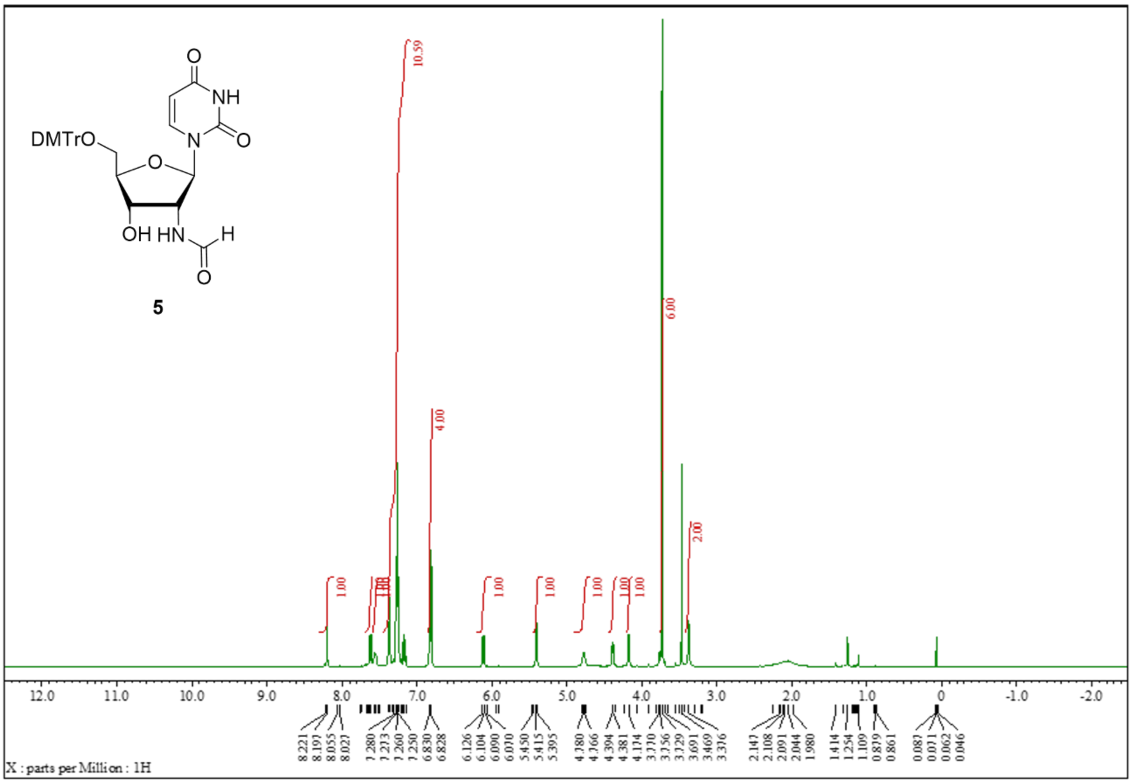

^{1}H-NMR (400 MHz, $CDCl_3$) of Compound **5**

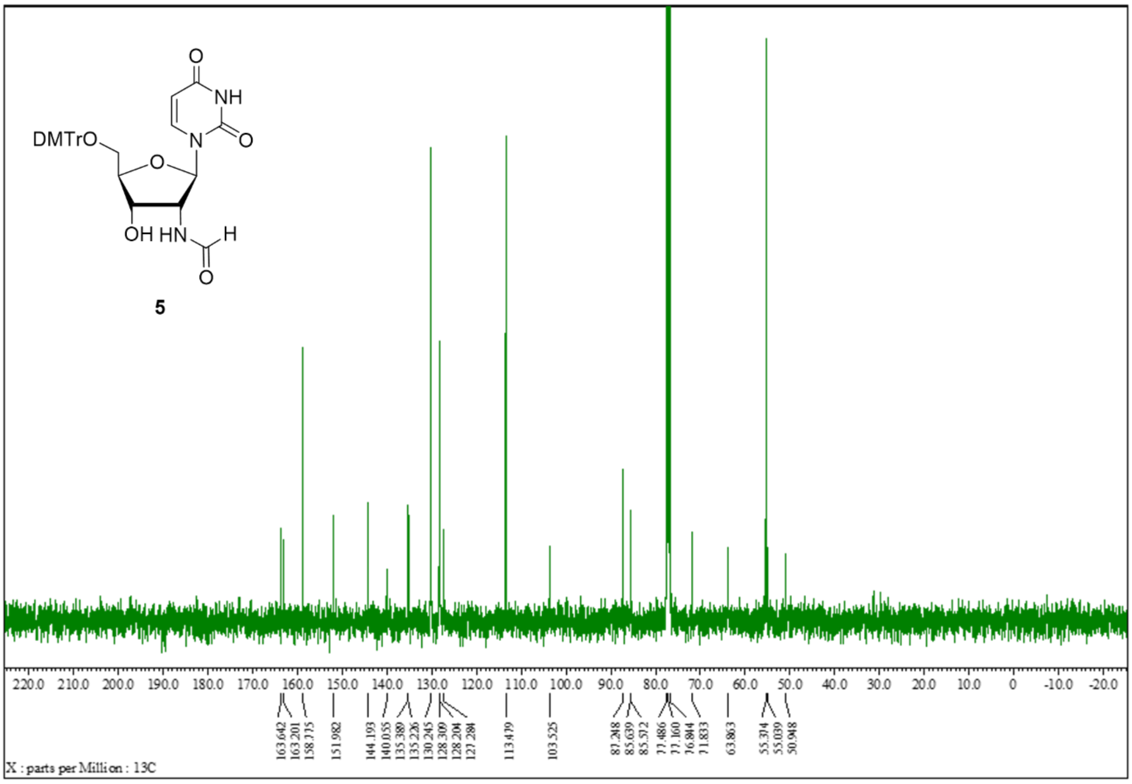

^{13}C-NMR (100 MHz, $CDCl_3$) of Compound **5**

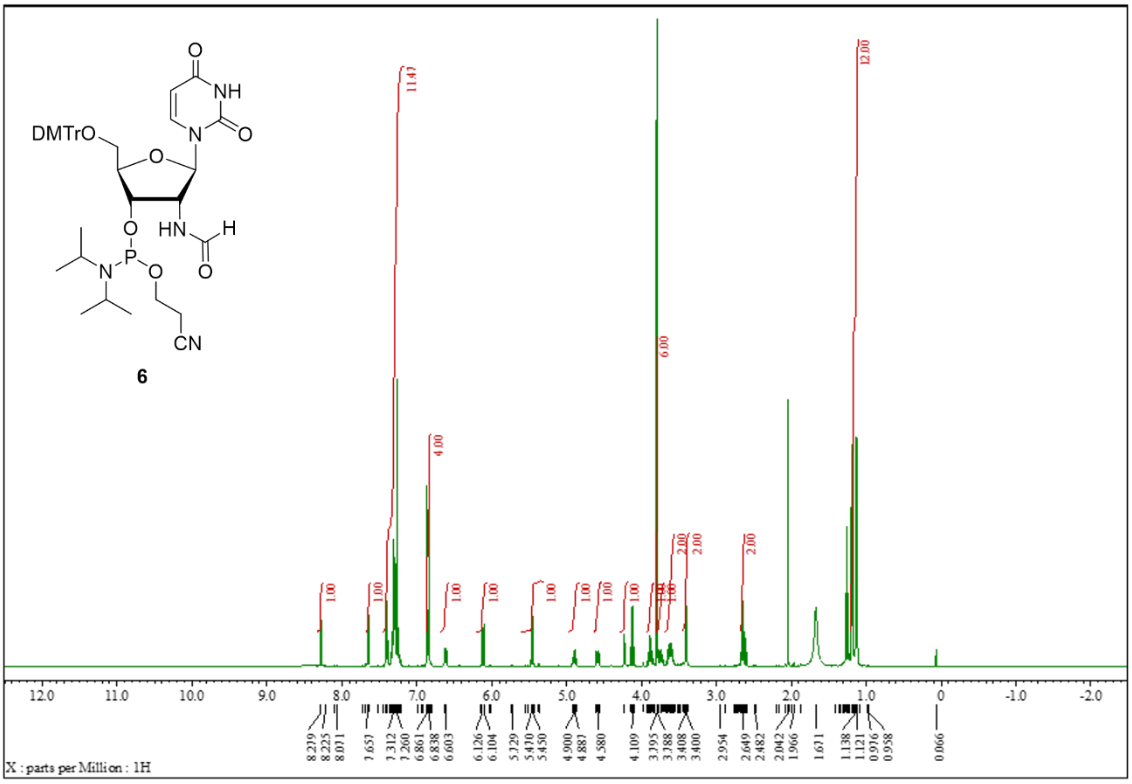

^{1}H-NMR (400 MHz, $CDCl_3$) of Compound **6**

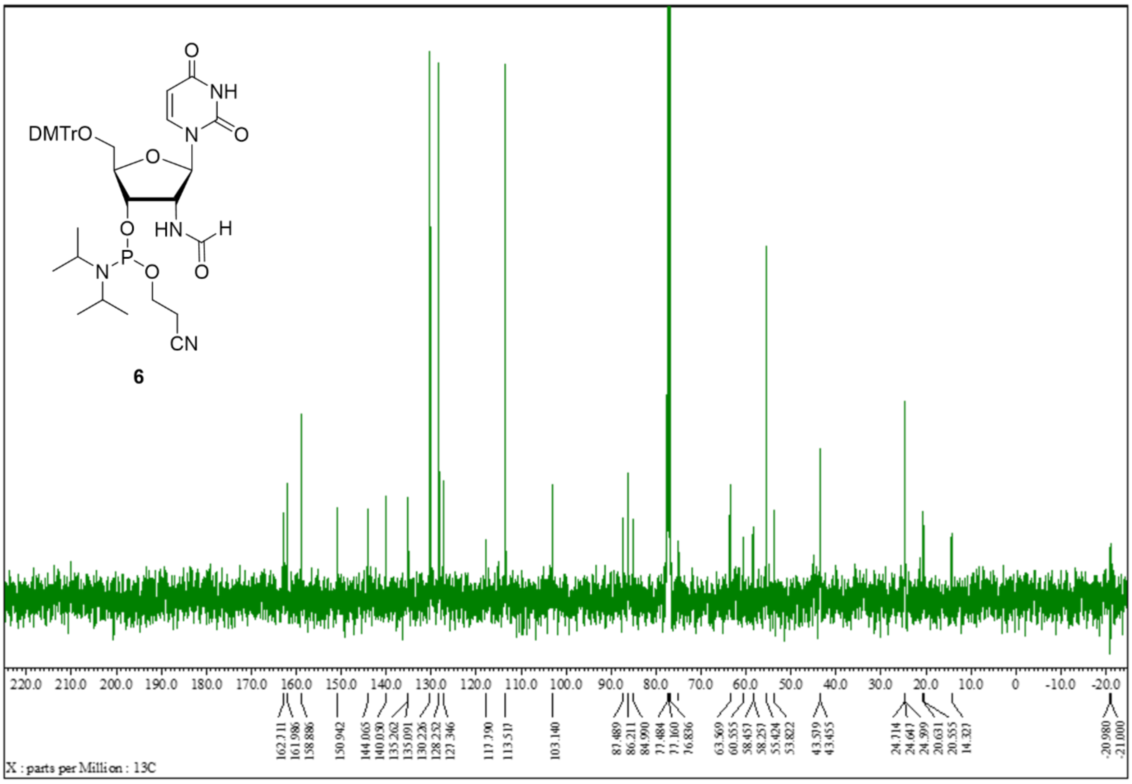

^{13}C-NMR (100 MHz, $CDCl_3$) of Compound **6**

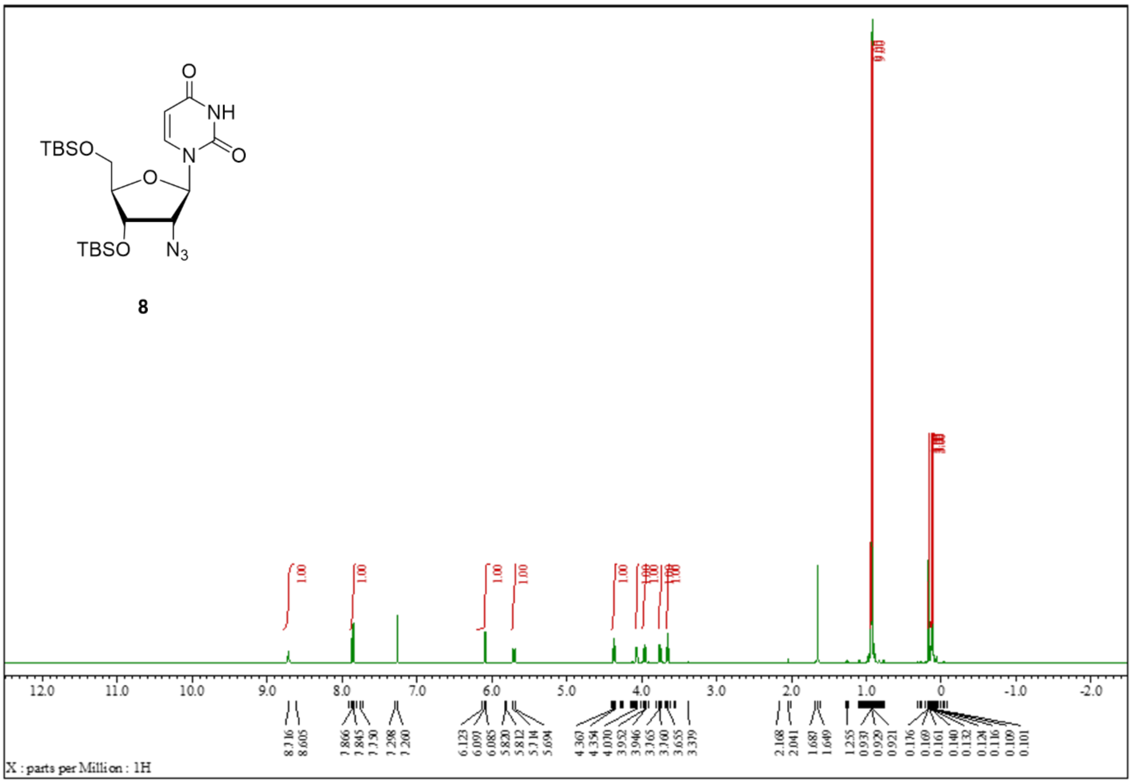

^{31}P-NMR (160 MHz, $CDCl_3$) of Compound **6**

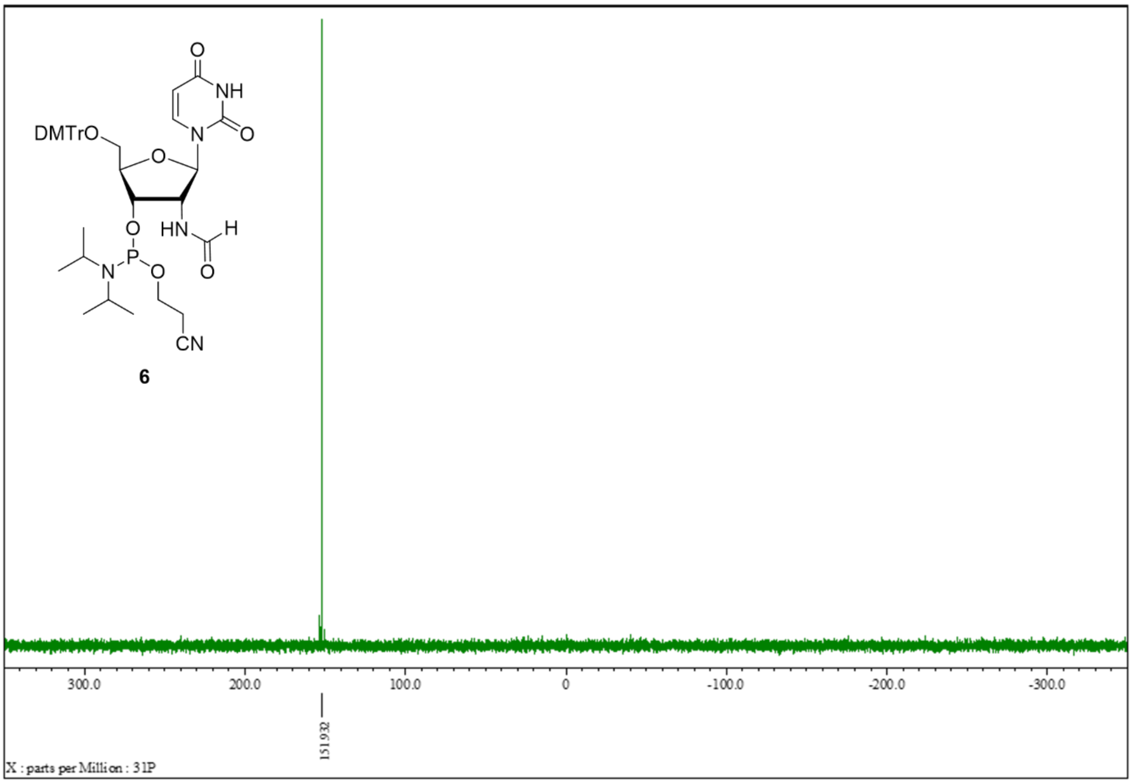

^{1}H-NMR (400 MHz, $CDCl_3$) of Compound **8**

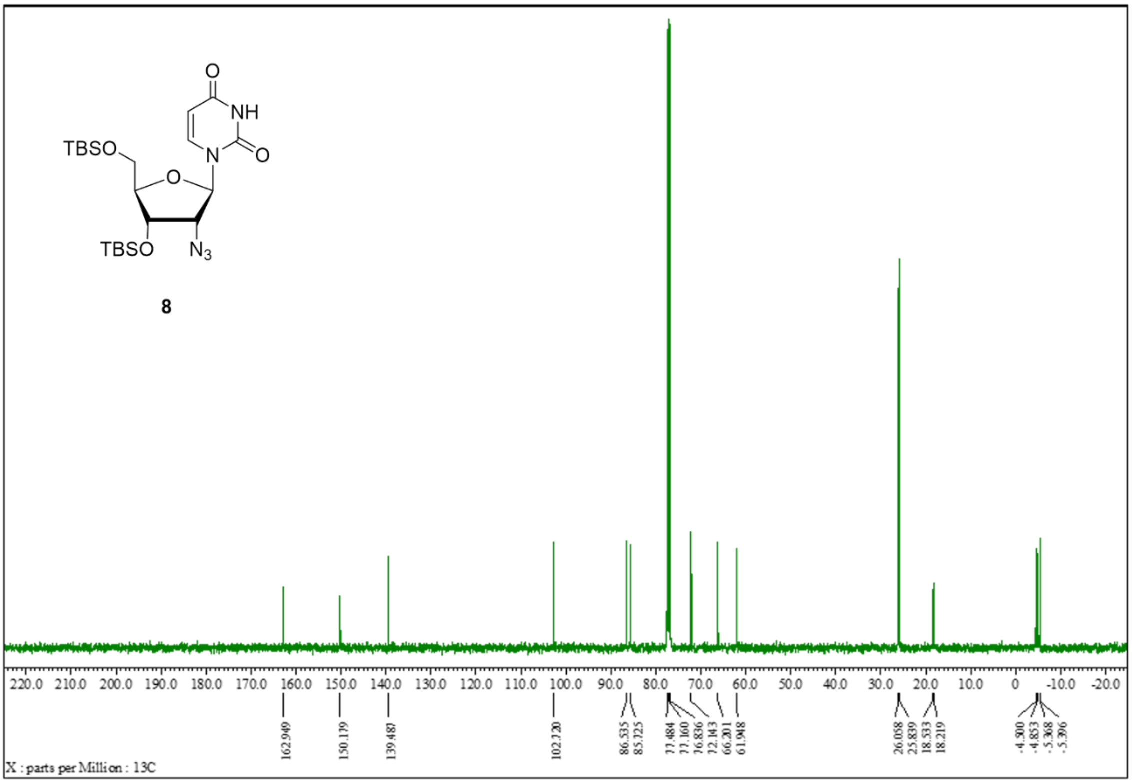

^{13}C-NMR (100 MHz, $CDCl_3$) of Compound **8**

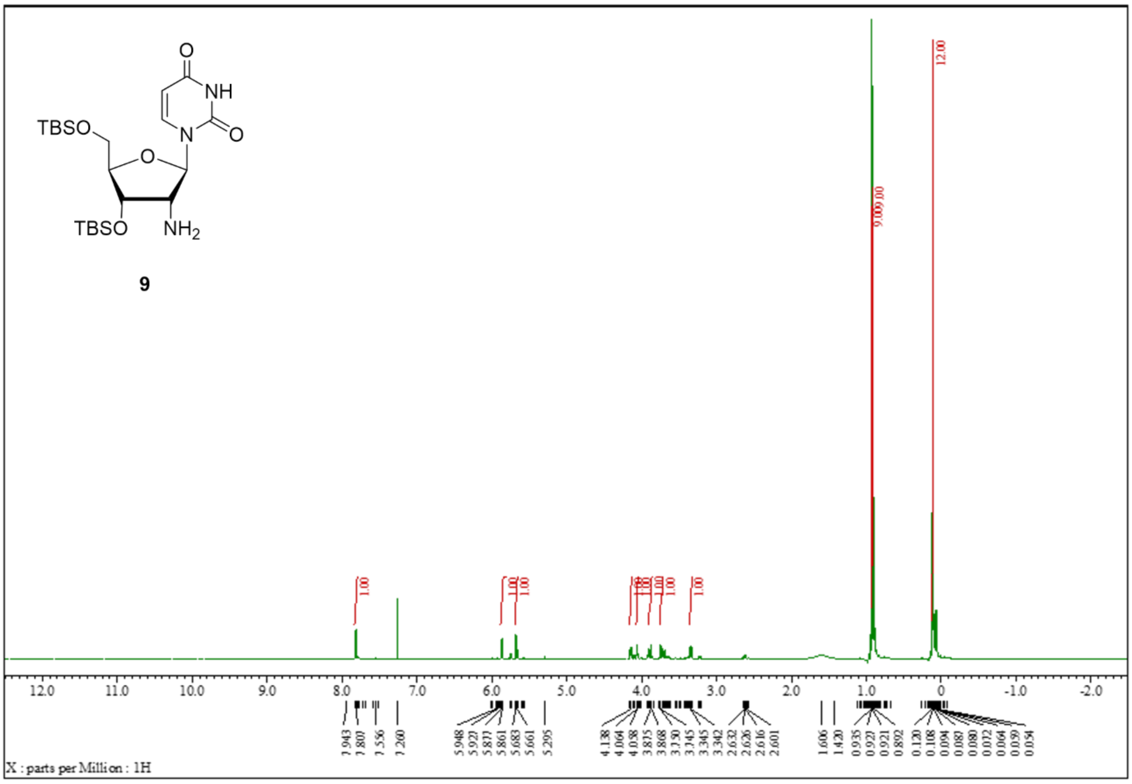

^{1}H-NMR (400 MHz, $CDCl_3$) of Compound **9**

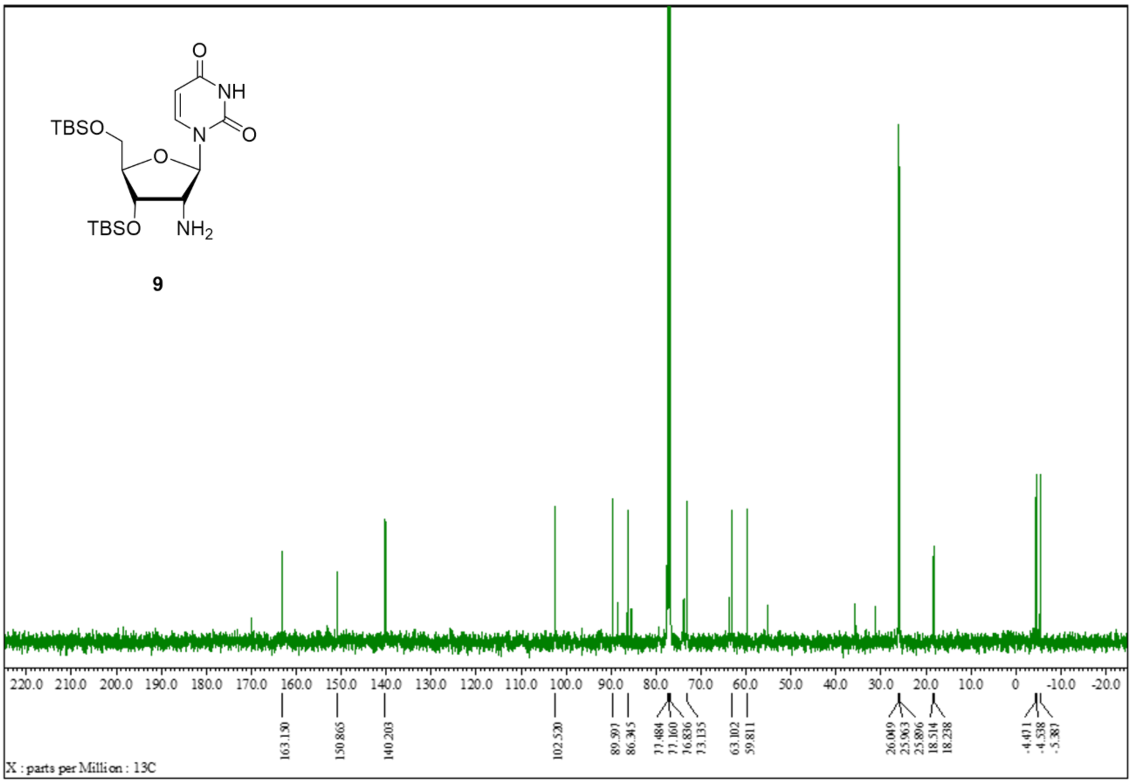

^{13}C-NMR (100 MHz, $CDCl_3$) of Compound **9**

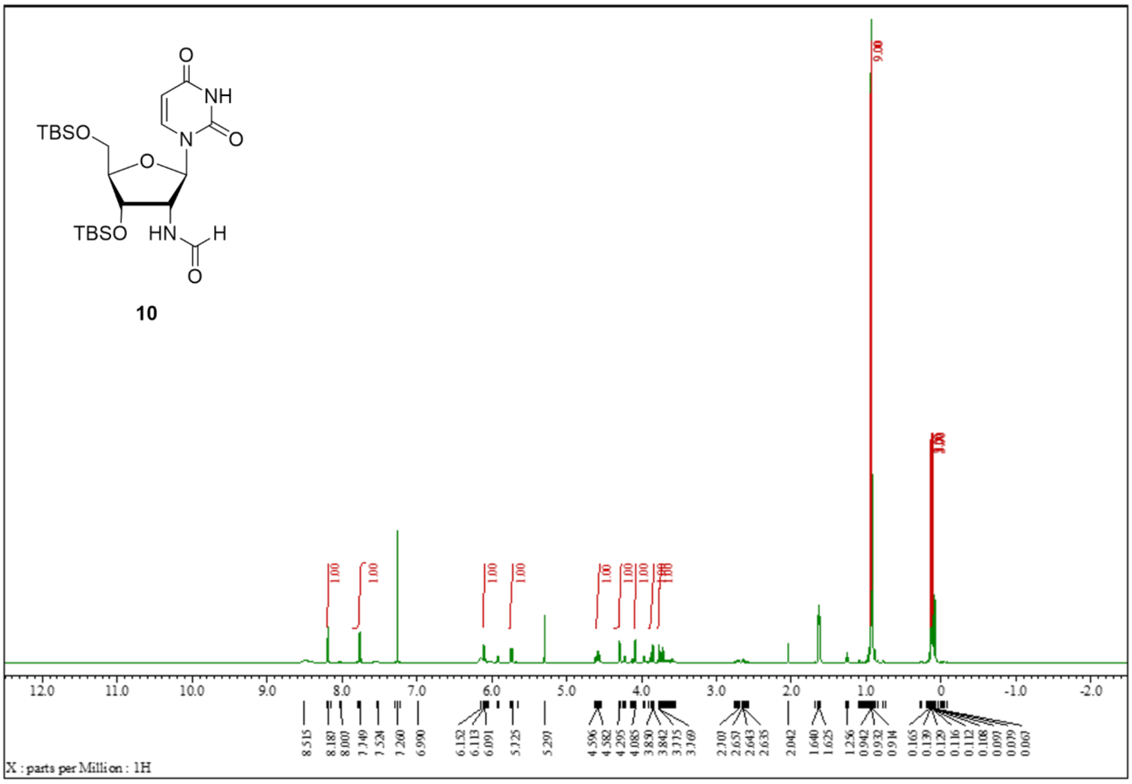

^{1}H-NMR (400 MHz, $CDCl_3$) of Compound **10**

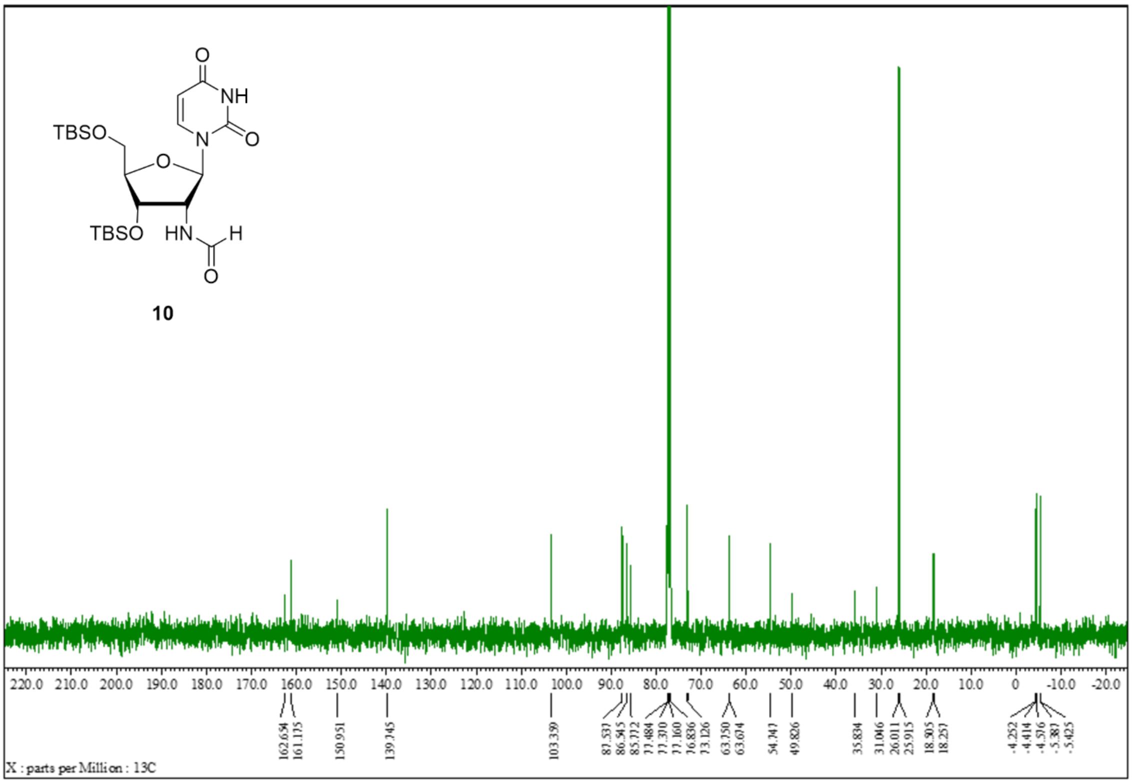

^{13}C-NMR (100 MHz, $CDCl_3$) of Compound **10**

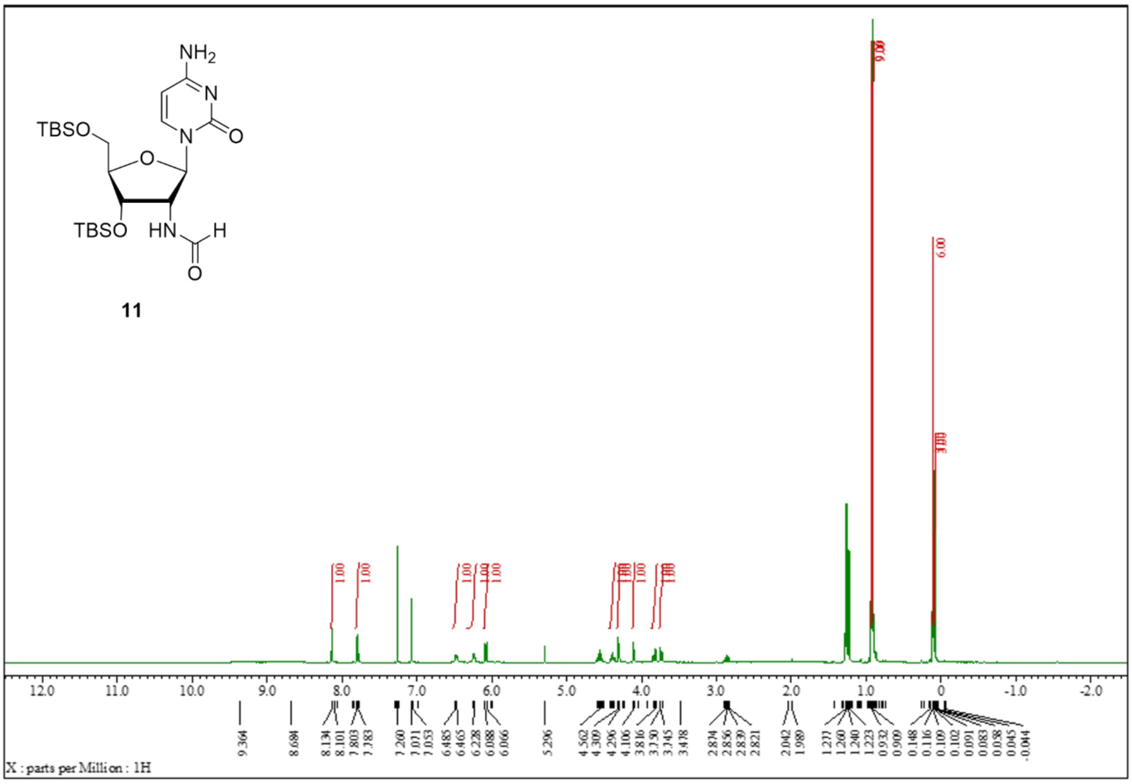

^{1}H-NMR (400 MHz, $CDCl_3$) of Compound **11**

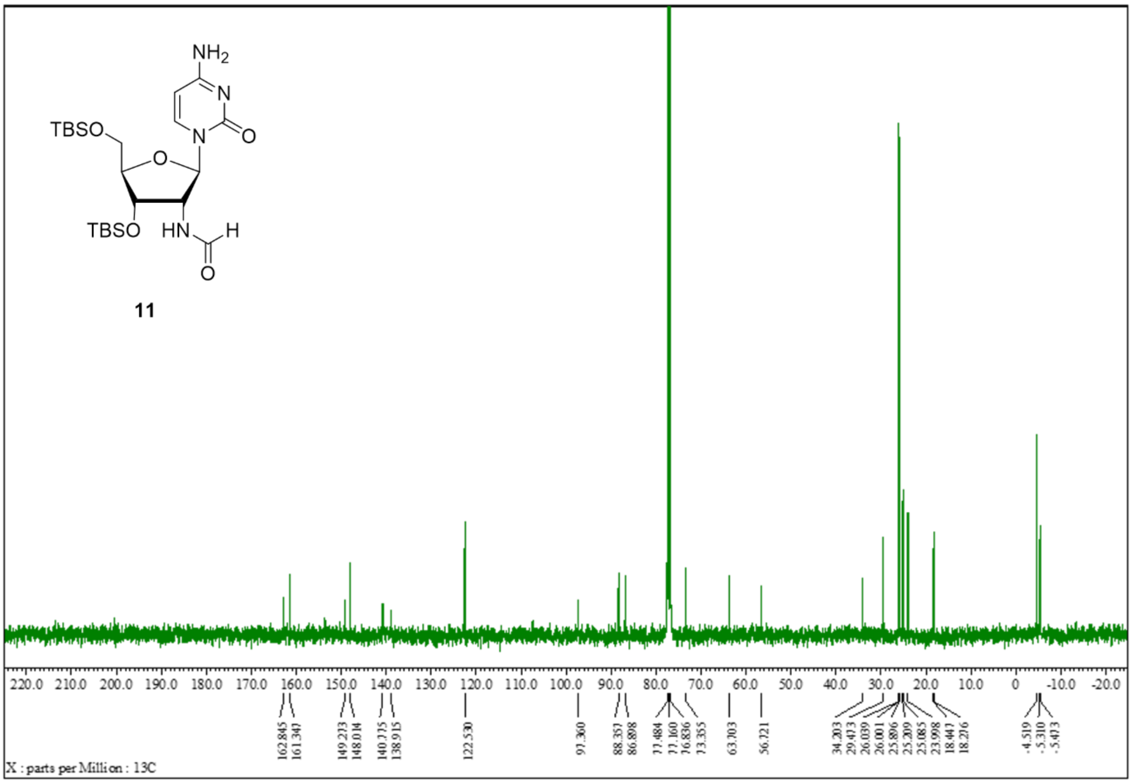

^{13}C-NMR (100 MHz, $CDCl_3$) of Compound **11**

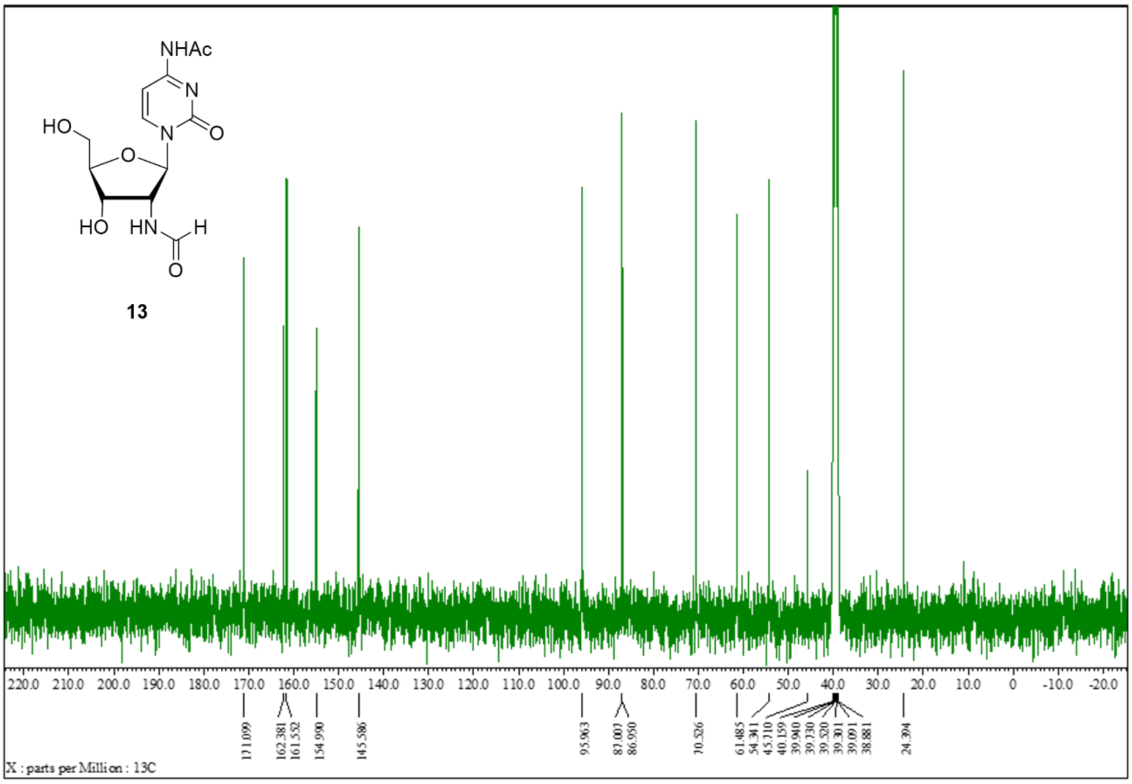

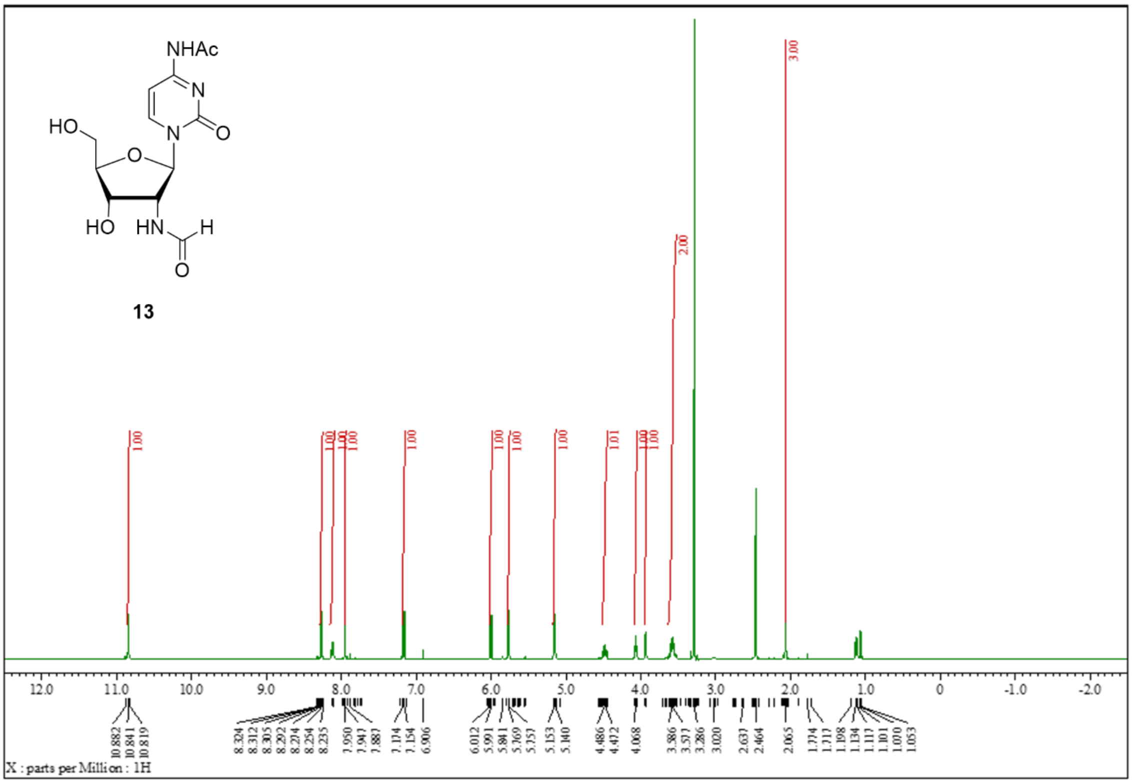

^{1}H-NMR (400 MHz, DMSO-d_6) of Compound **13**

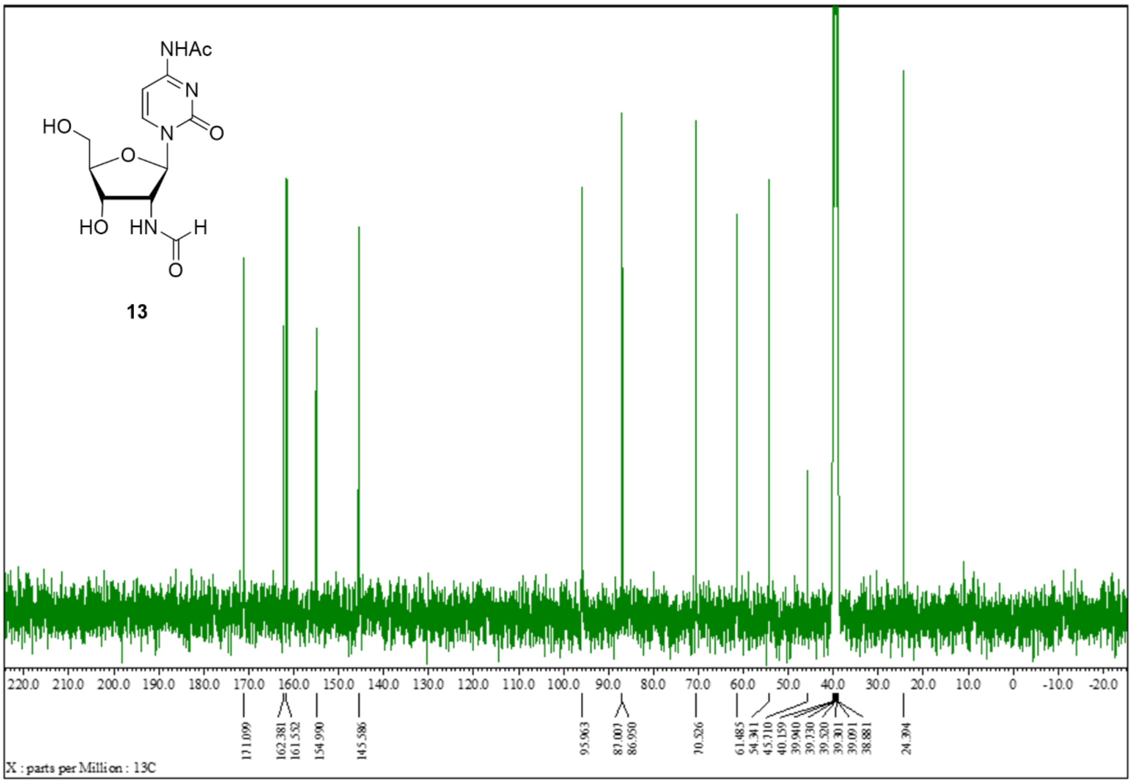

^{13}C-NMR (100 MHz, DMSO-d_6) of Compound **13**

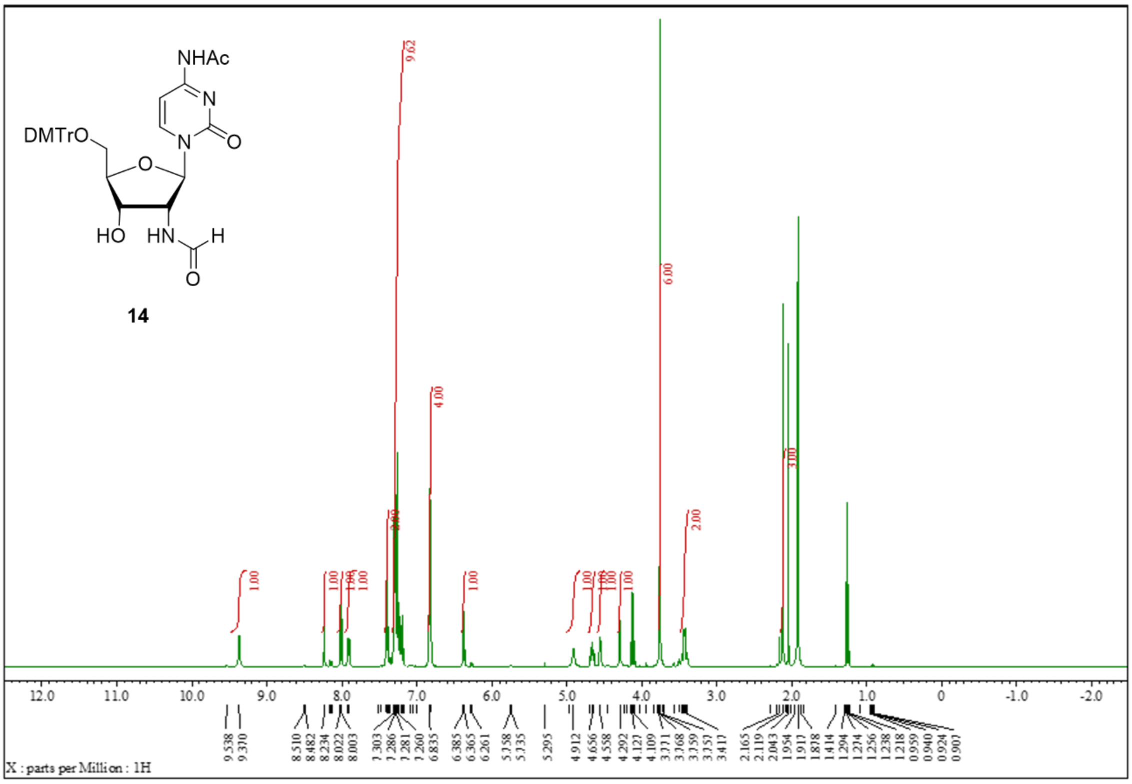

^{1}H-NMR (400 MHz, $CDCl_3$) of Compound **14**

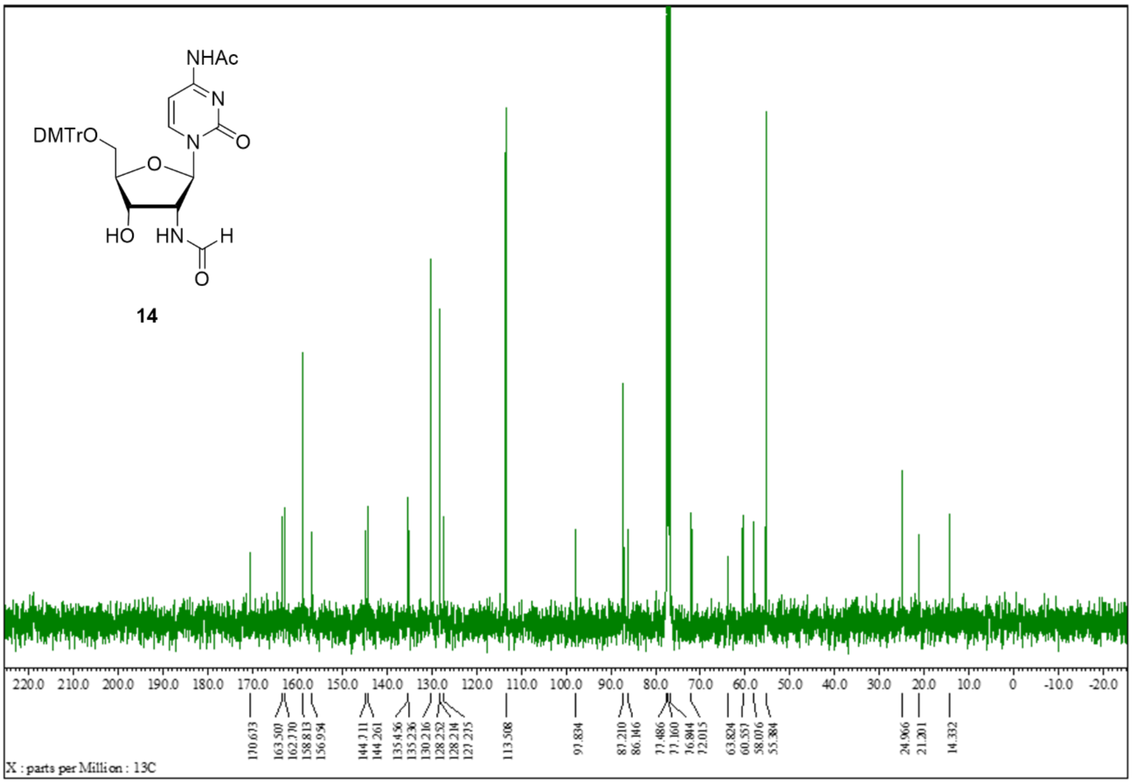

^{13}C-NMR (100 MHz, $CDCl_3$) of Compound **14**

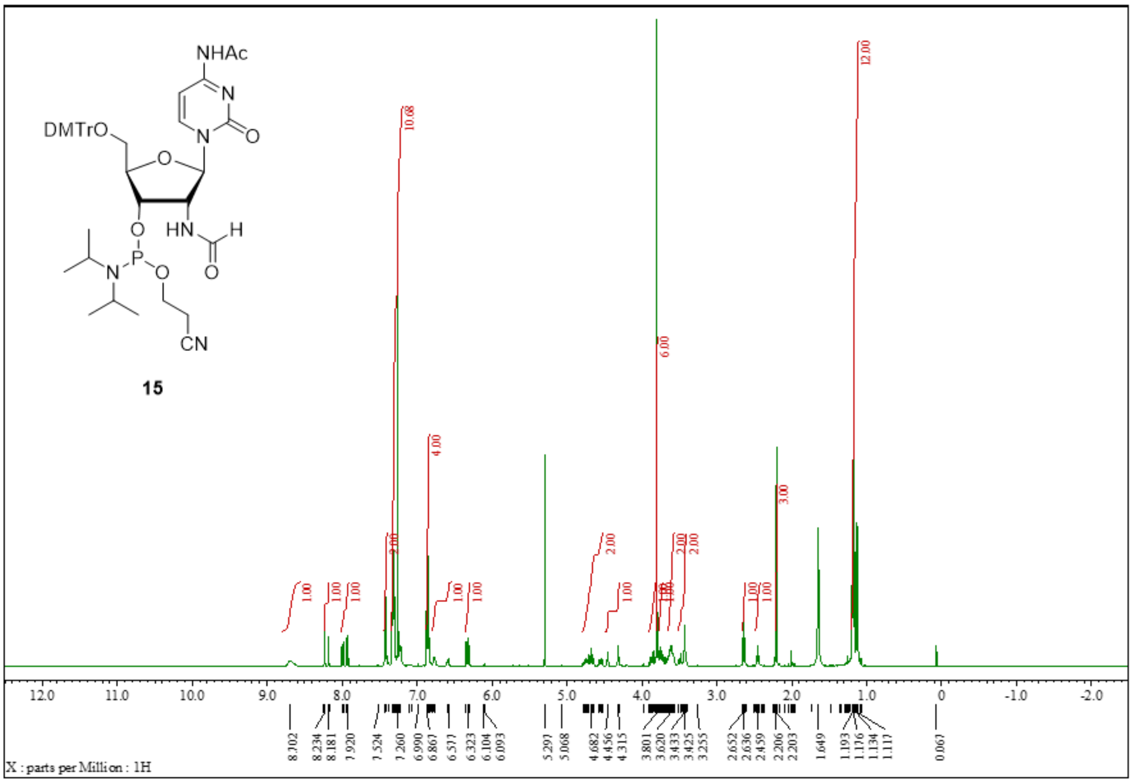

^{1}H-NMR (400 MHz, $CDCl_3$) of Compound **15**

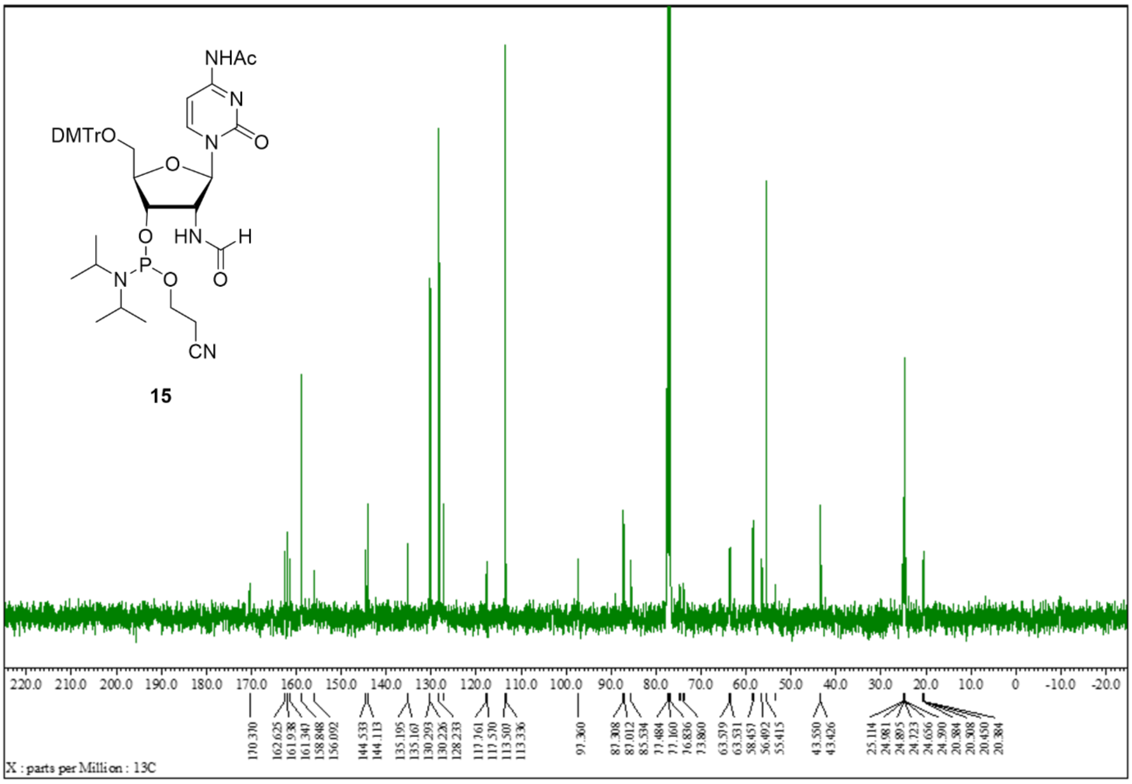

^{13}C-NMR (100 MHz, $CDCl_3$) of Compound **15**

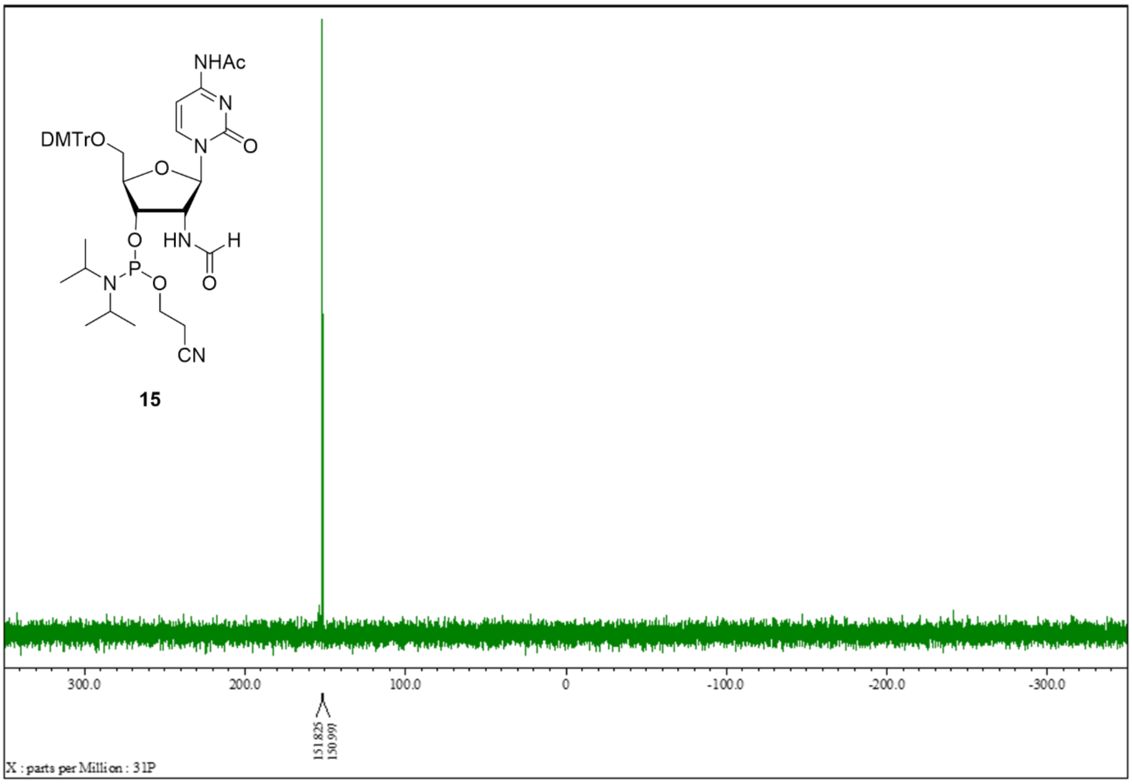

^{31}P-NMR (160 MHz, $CDCl_3$) of Compound **15**

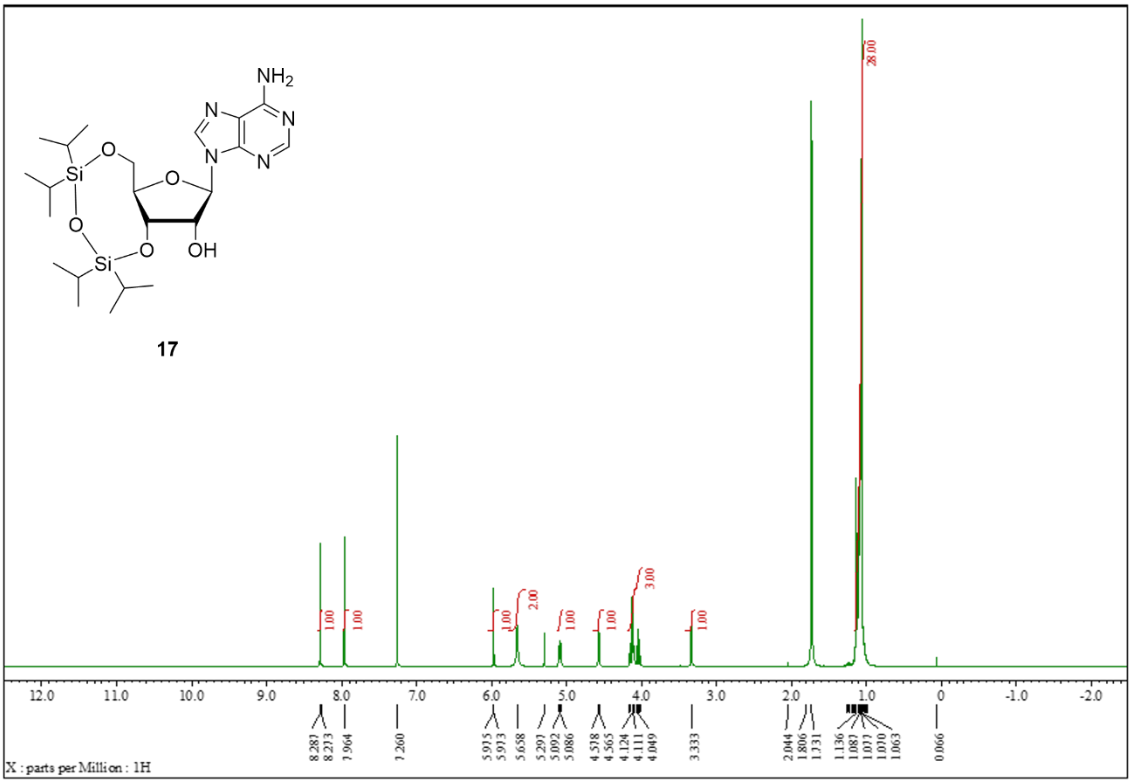

^{1}H-NMR (400 MHz, $CDCl_3$) of Compound **17**

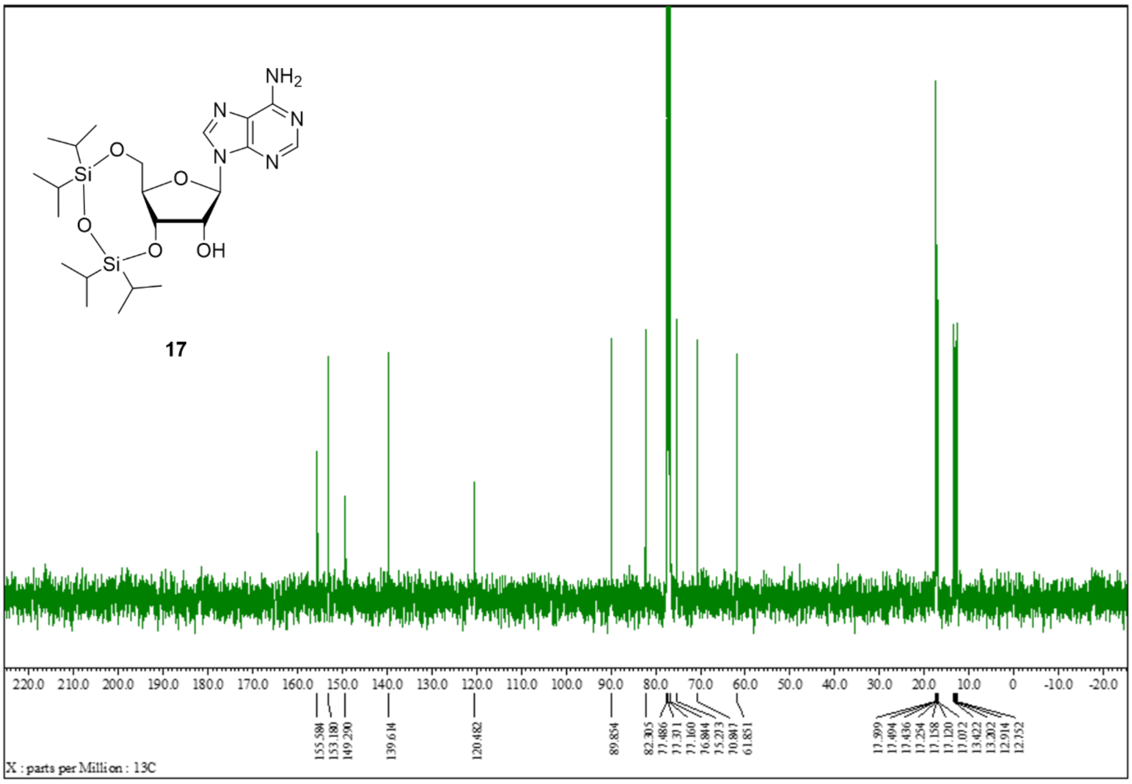

^{13}C-NMR (100 MHz, $CDCl_3$) of Compound **17**

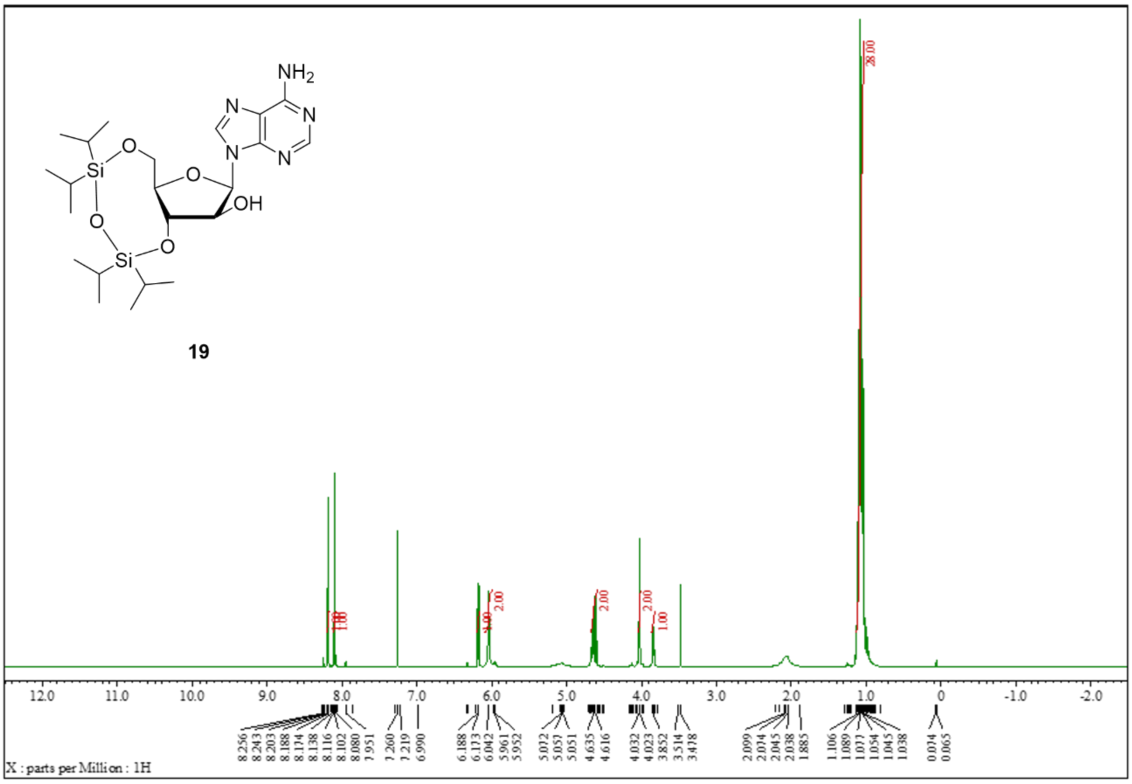

^{1}H-NMR (400 MHz, $CDCl_3$) of Compound **19**

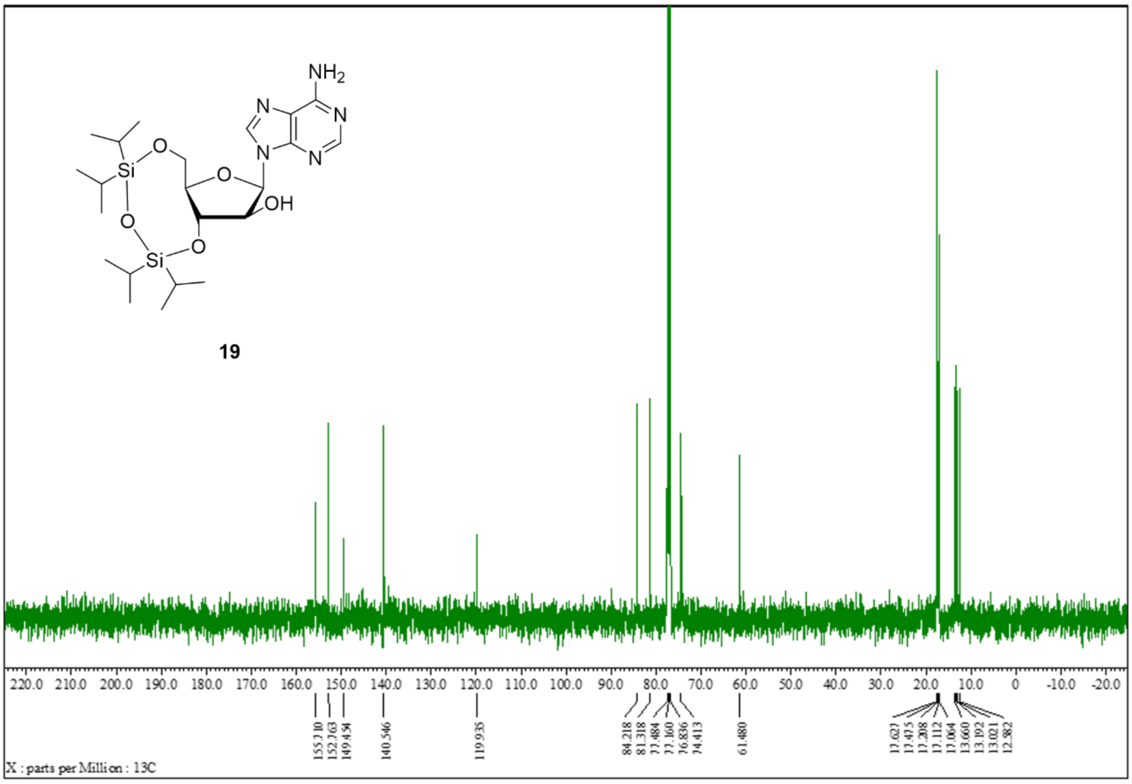

^{13}C-NMR (100 MHz, $CDCl_3$) of Compound **19**

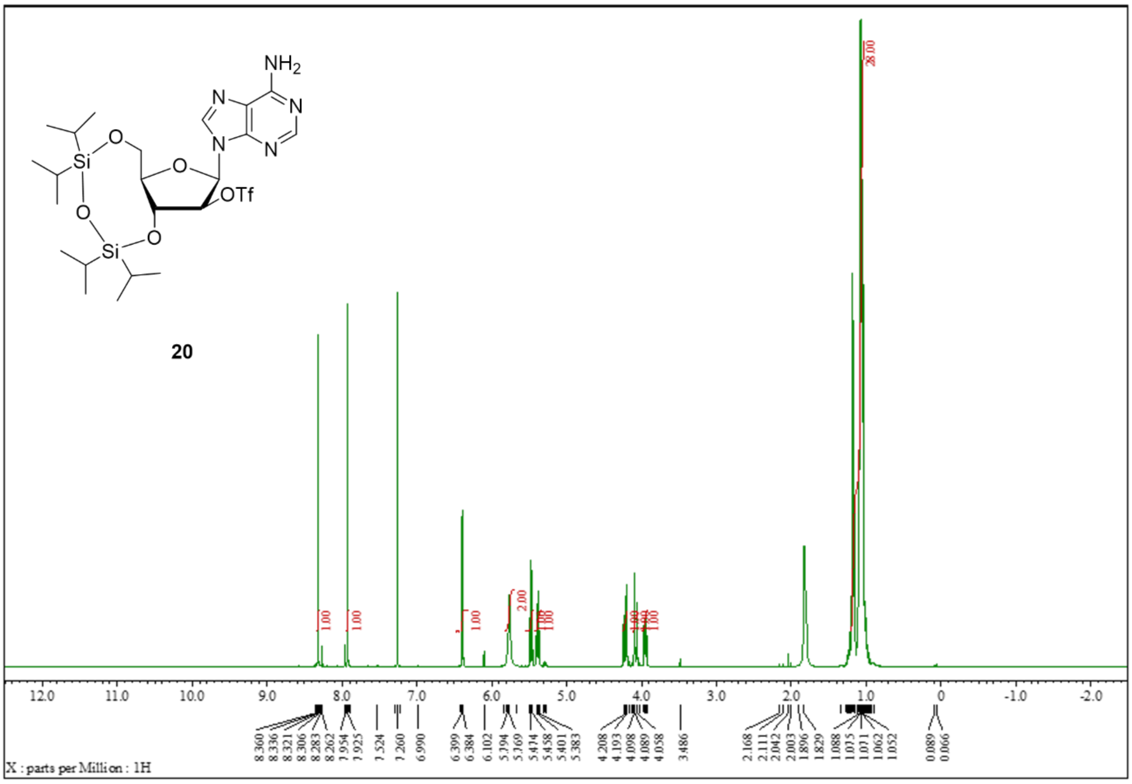

^{1}H-NMR (400 MHz, $CDCl_3$) of Compound **20**

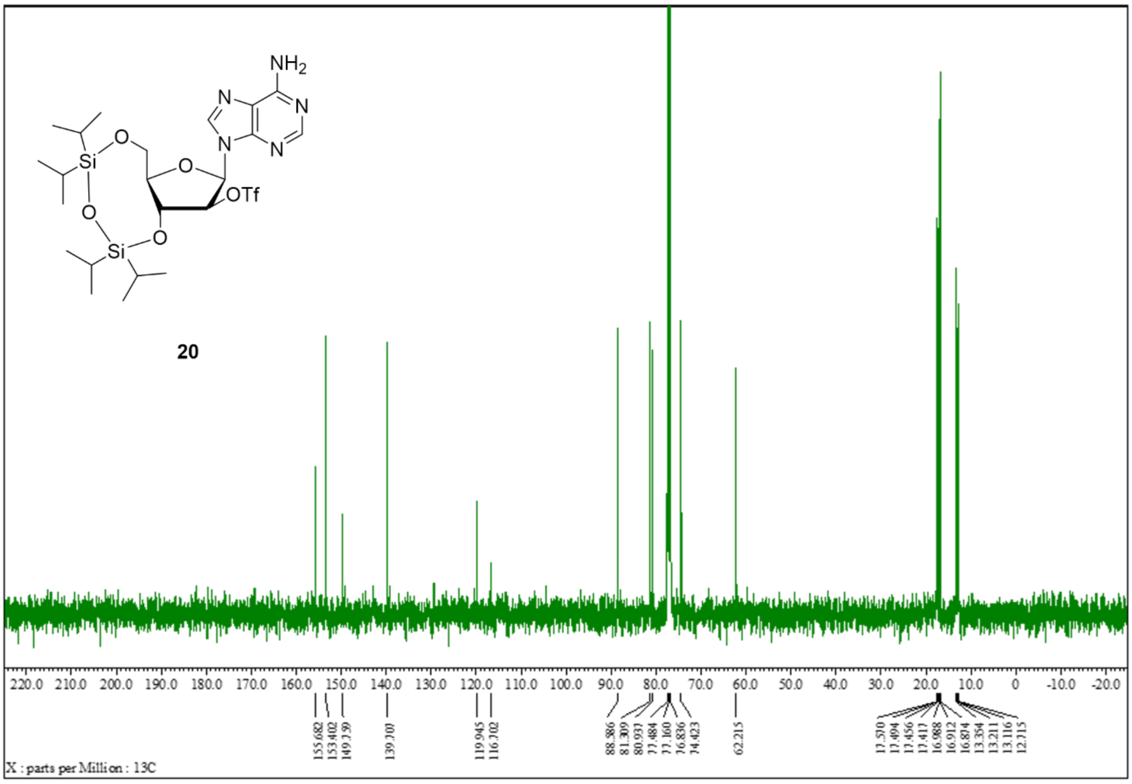

^{13}C-NMR (100 MHz, $CDCl_3$) of Compound **20**

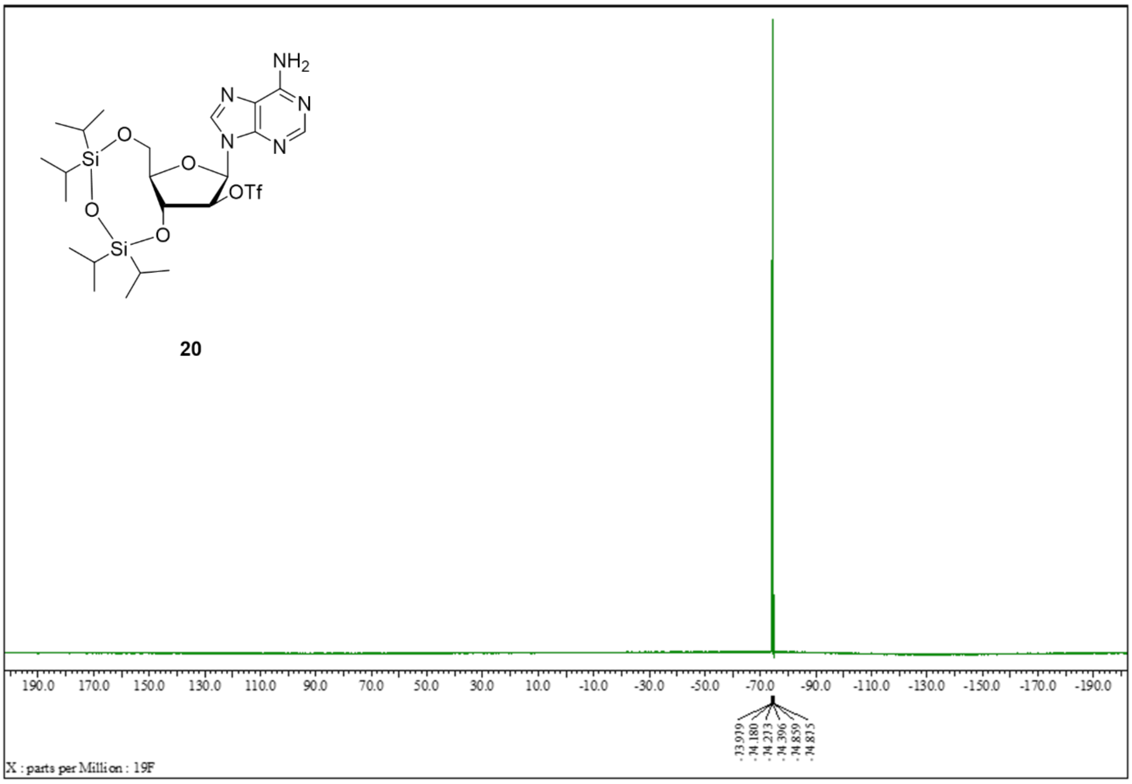

^{19}F-NMR (380 MHz, $CDCl_3$) of Compound **20**

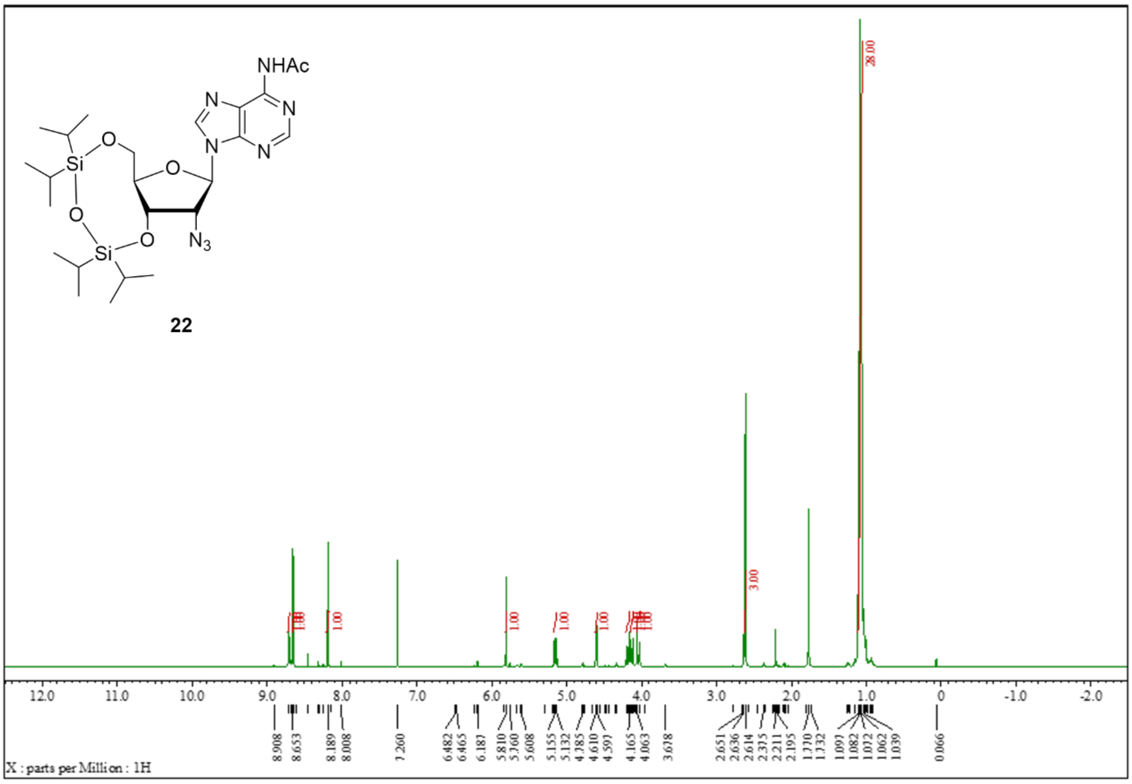

^{1}H-NMR (400 MHz, $CDCl_3$) of Compound **22**

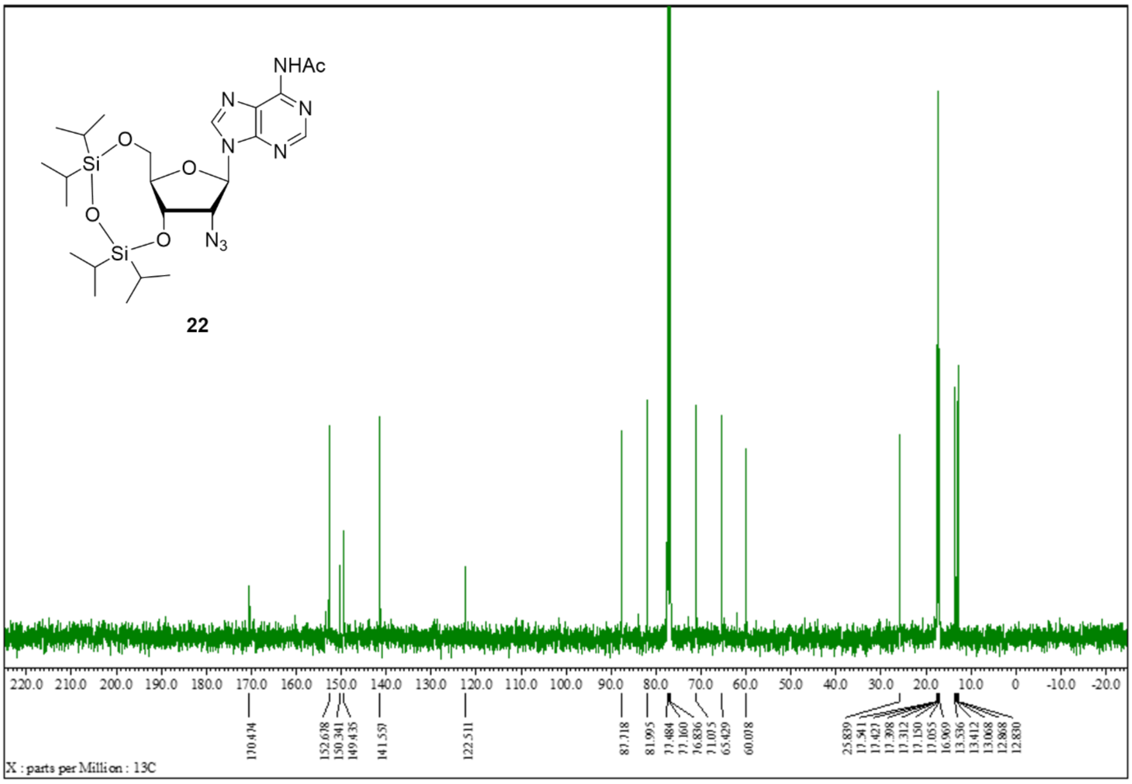

^{13}C-NMR (100 MHz, $CDCl_3$) of Compound **22**

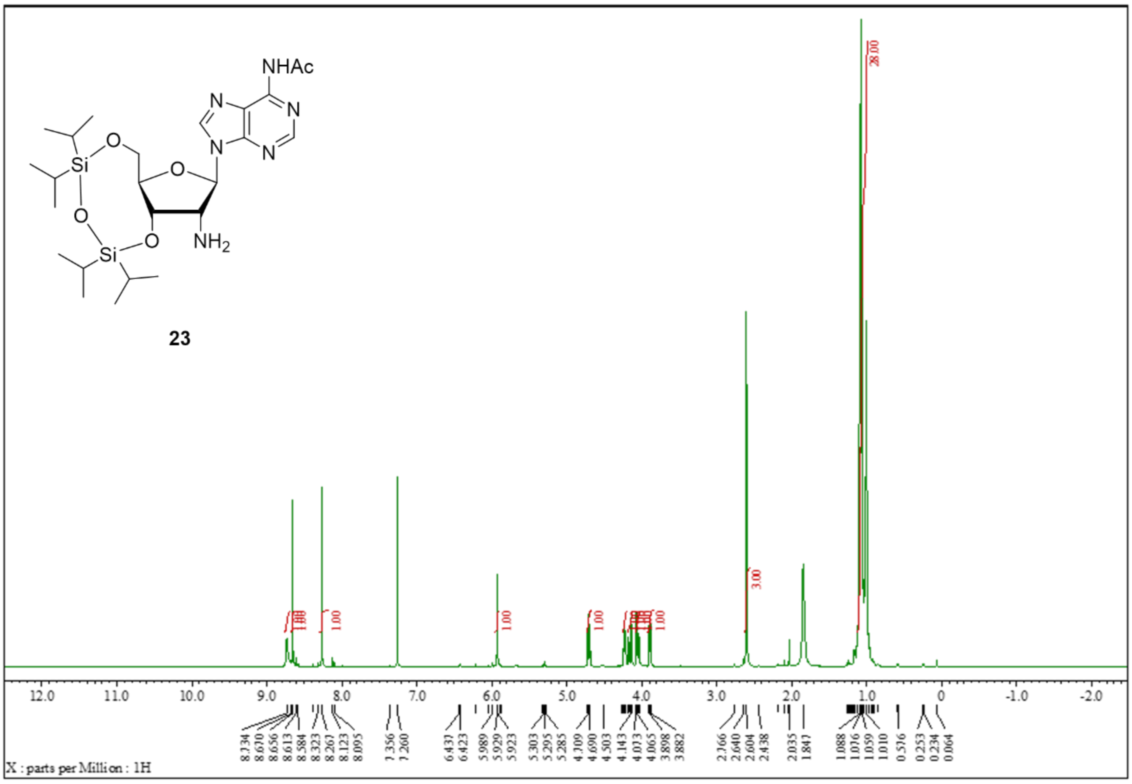

^{1}H-NMR (400 MHz, $CDCl_3$) of Compound **23**

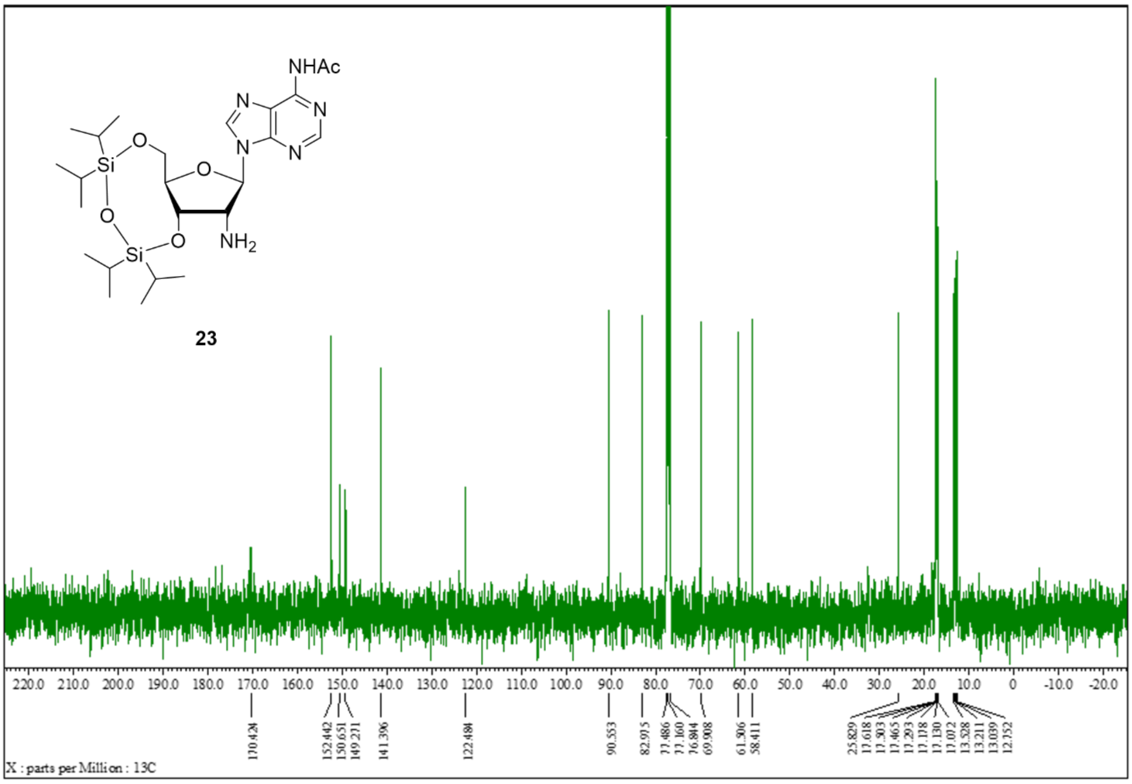

^{13}C-NMR (100 MHz, $CDCl_3$) of Compound **23**

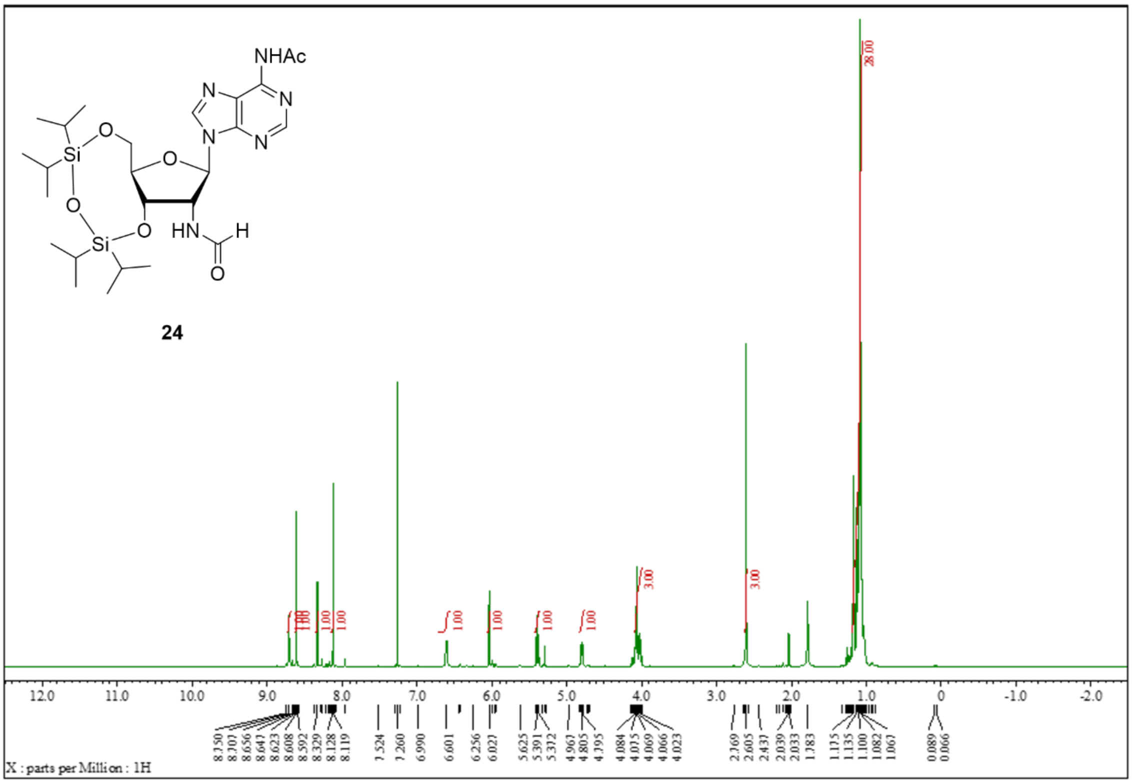

^{1}H-NMR (400 MHz, $CDCl_3$) of Compound **24**

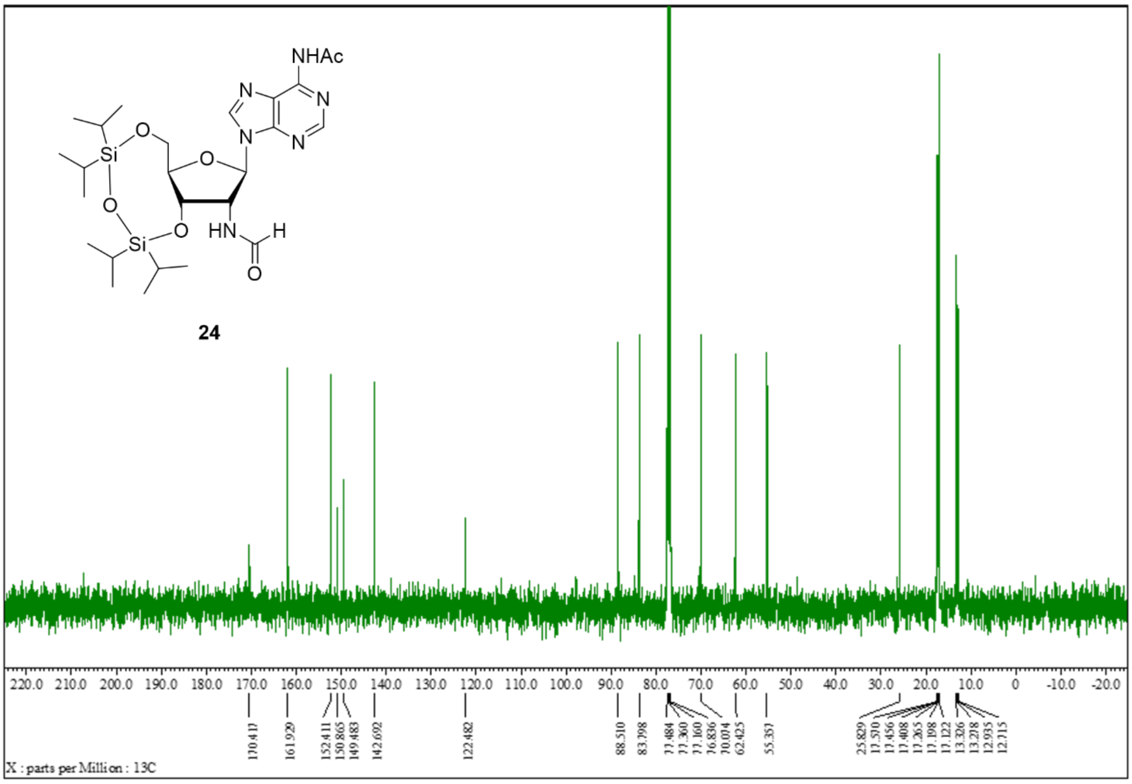

^{13}C-NMR (100 MHz, $CDCl_3$) of Compound **24**

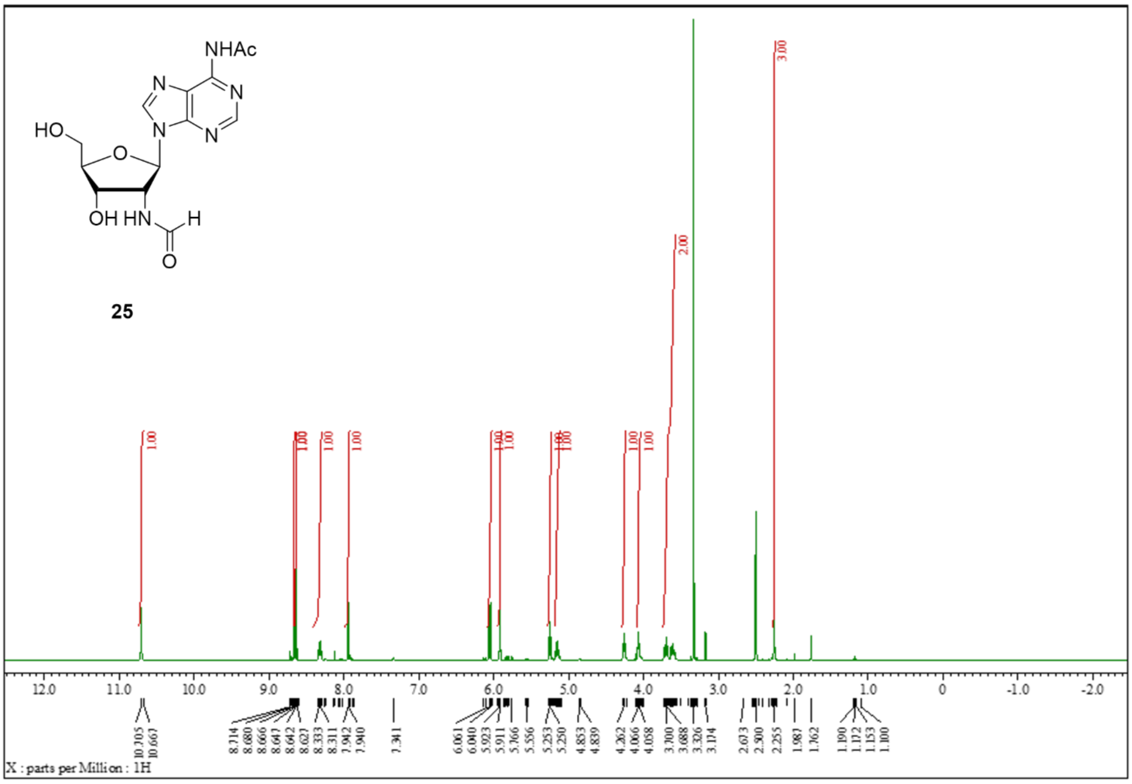

^{1}H-NMR (400 MHz, DMSO-d_6) of Compound **25**

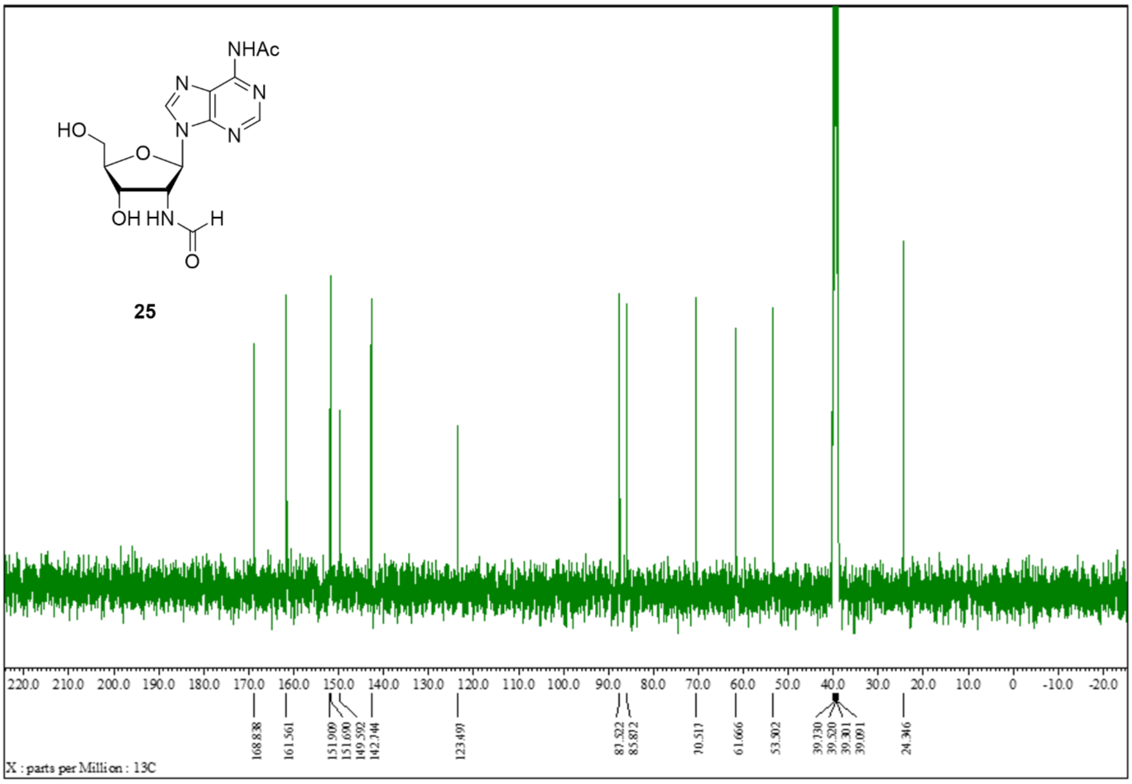

^{13}C-NMR (100 MHz, DMSO-d_6) of Compound **25**

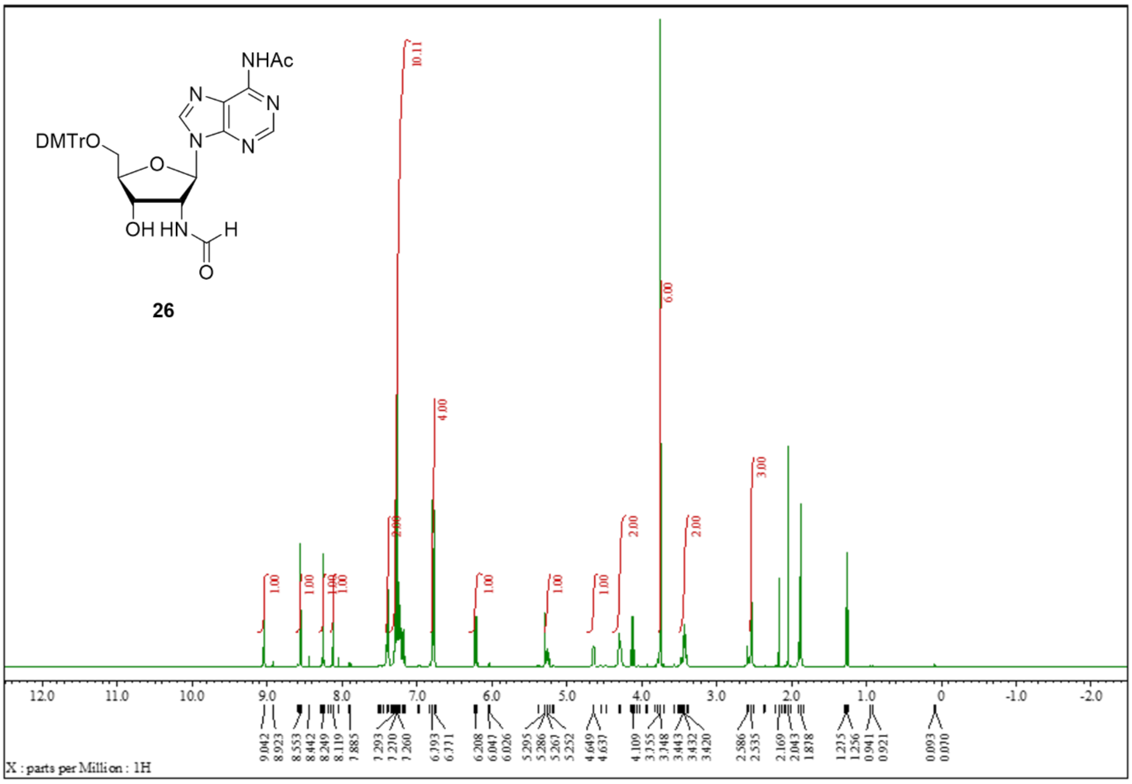

^{1}H-NMR (400 MHz, $CDCl_3$) of Compound **26**

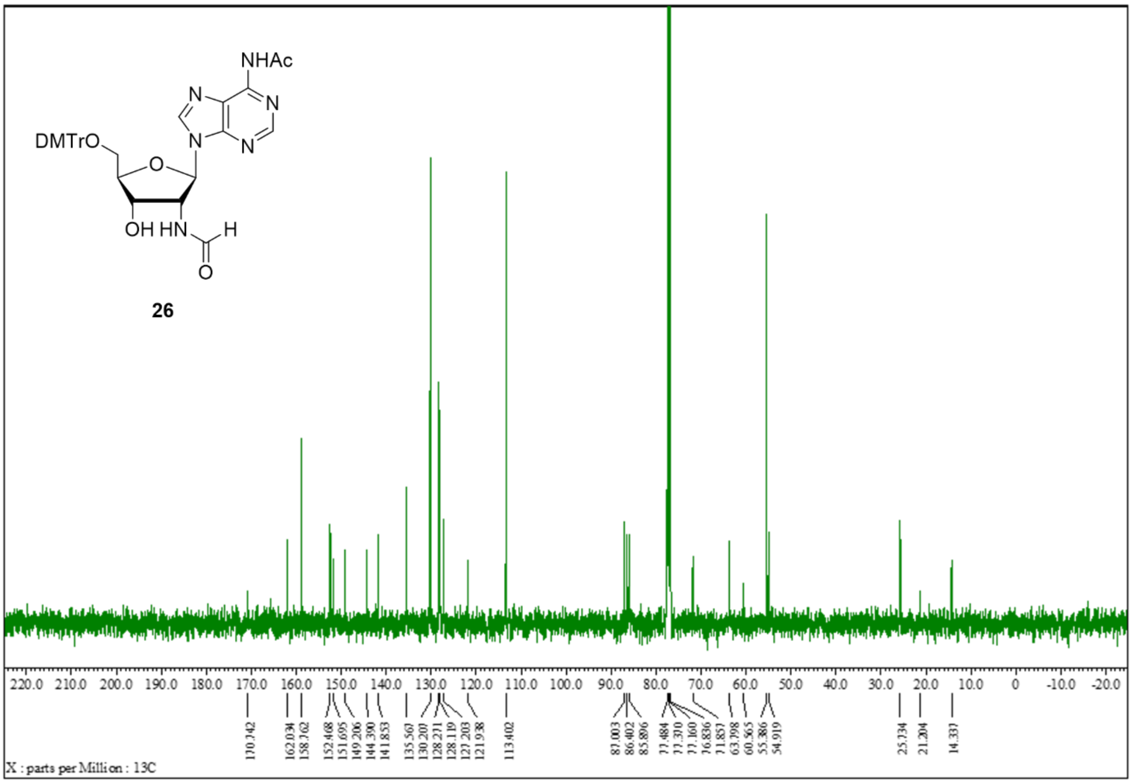

^{13}C-NMR (100 MHz, $CDCl_3$) of Compound **26**

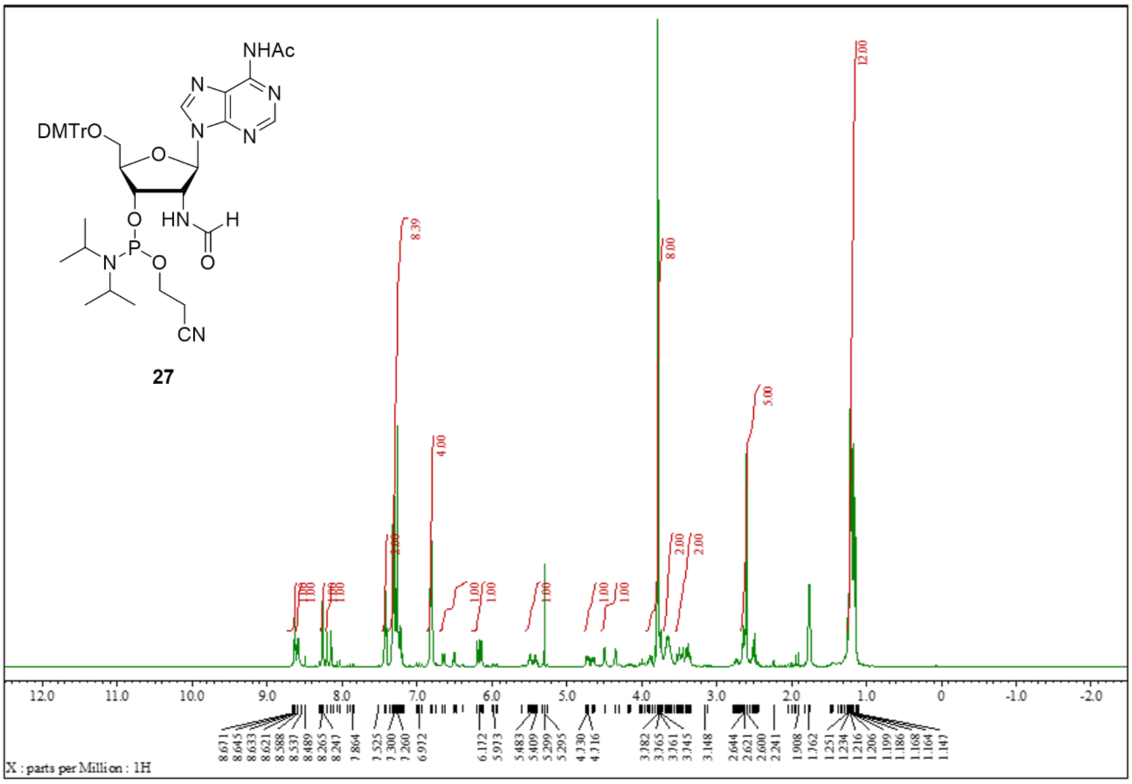

^{1}H-NMR (400 MHz, $CDCl_3$) of Compound **27**

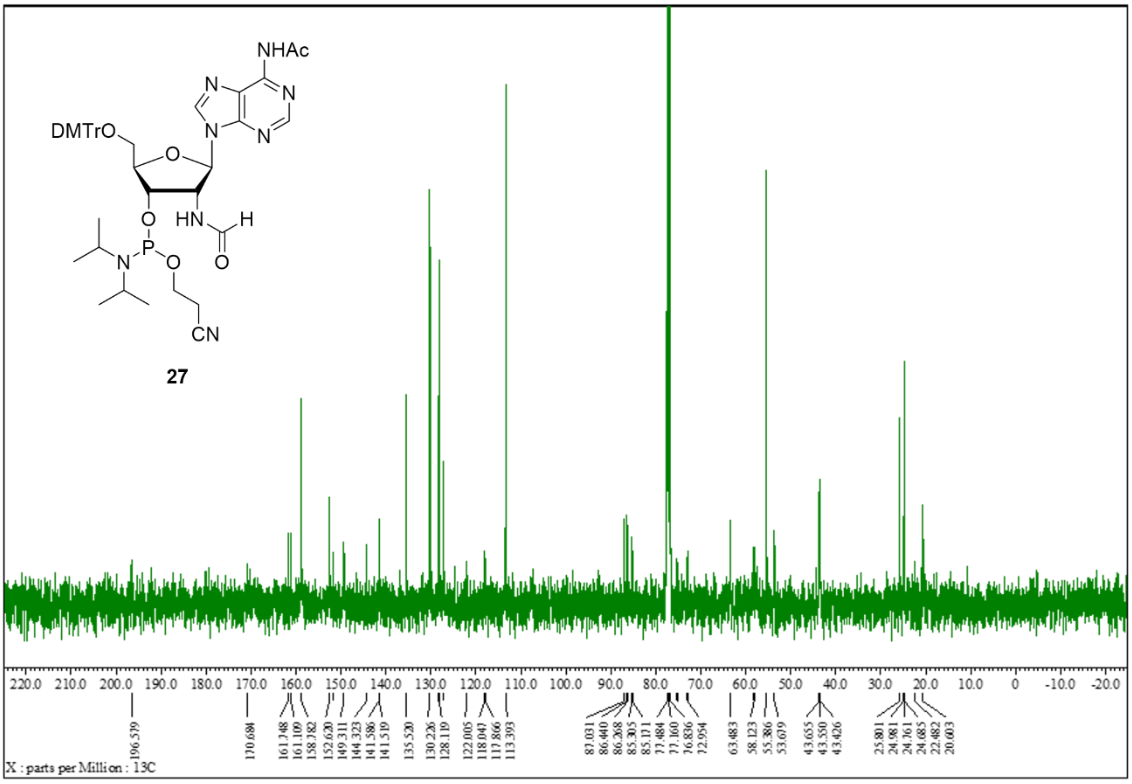

^{13}C-NMR (100 MHz, $CDCl_3$) of Compound **27**

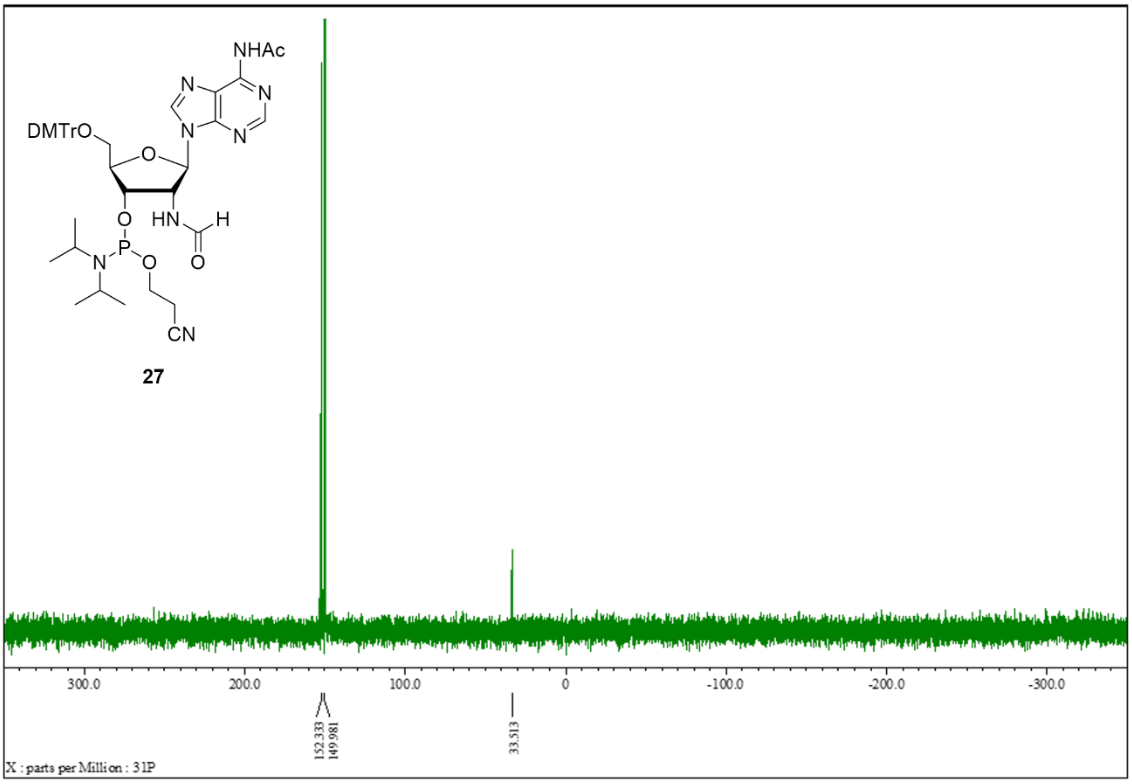

^{31}P-NMR (160 MHz, $CDCl_3$) of Compound **27**

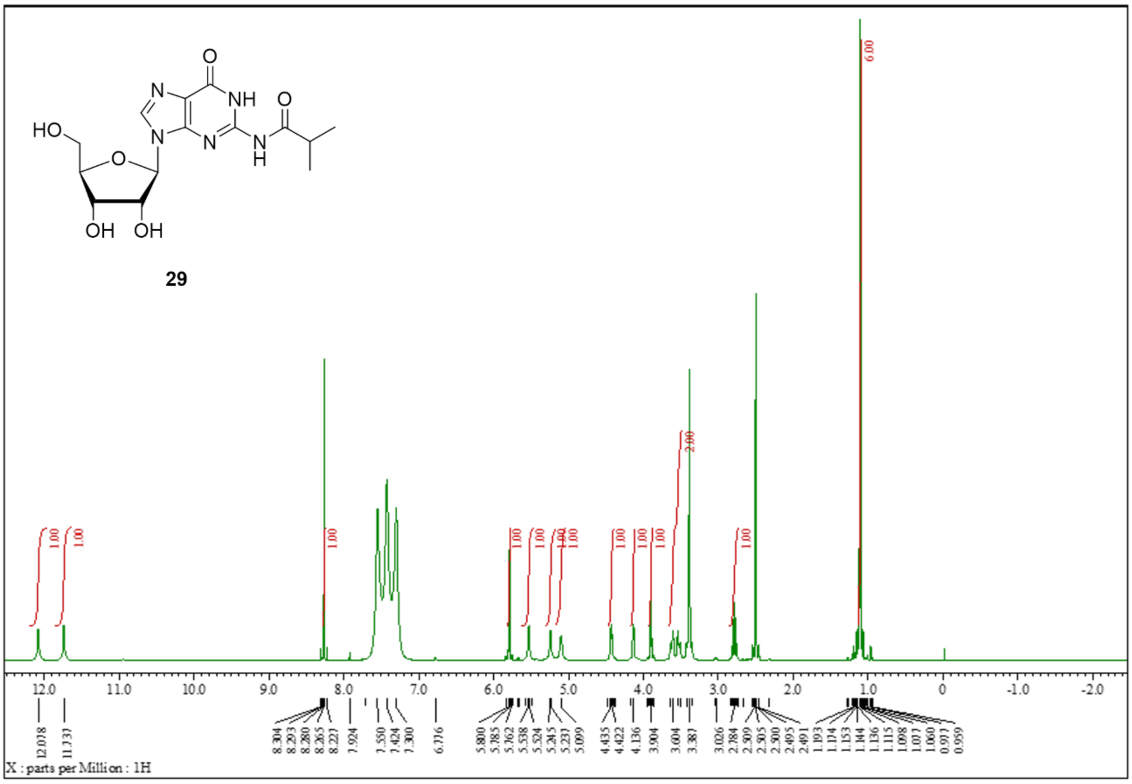

^{1}H-NMR (400 MHz, DMSO-d_6) of Compound **29**

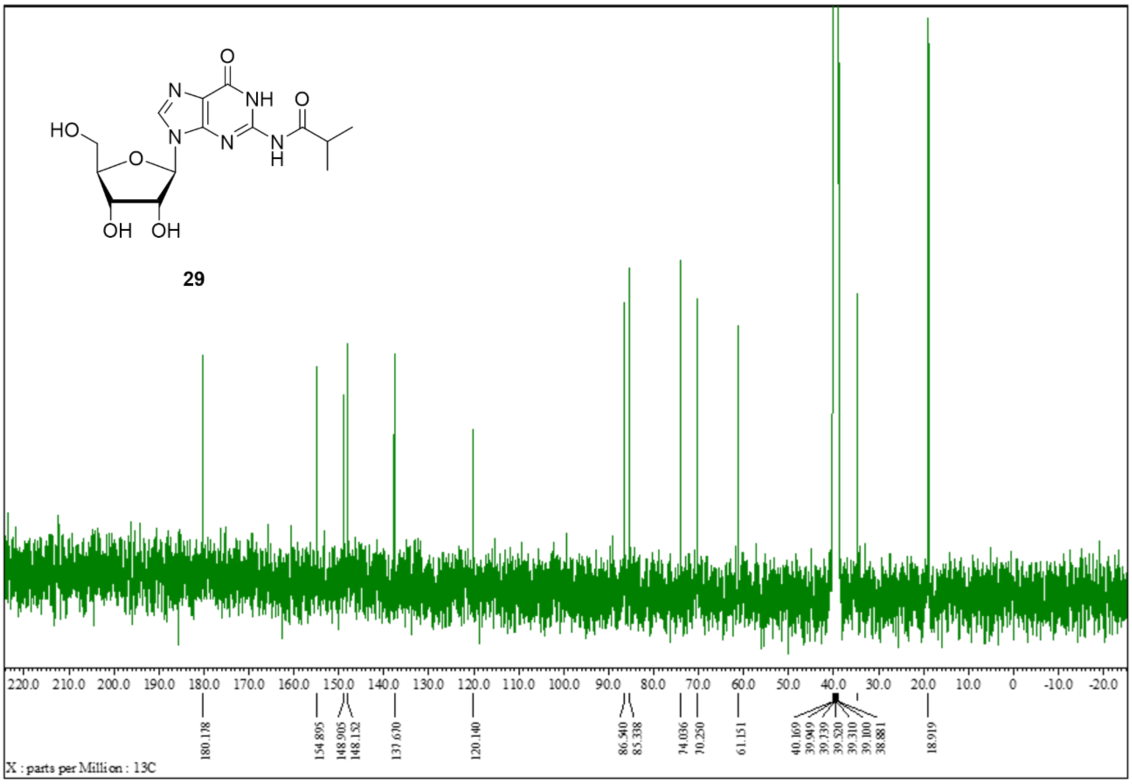

^{13}C-NMR (100 MHz, DMSO-d_6) of Compound **29**

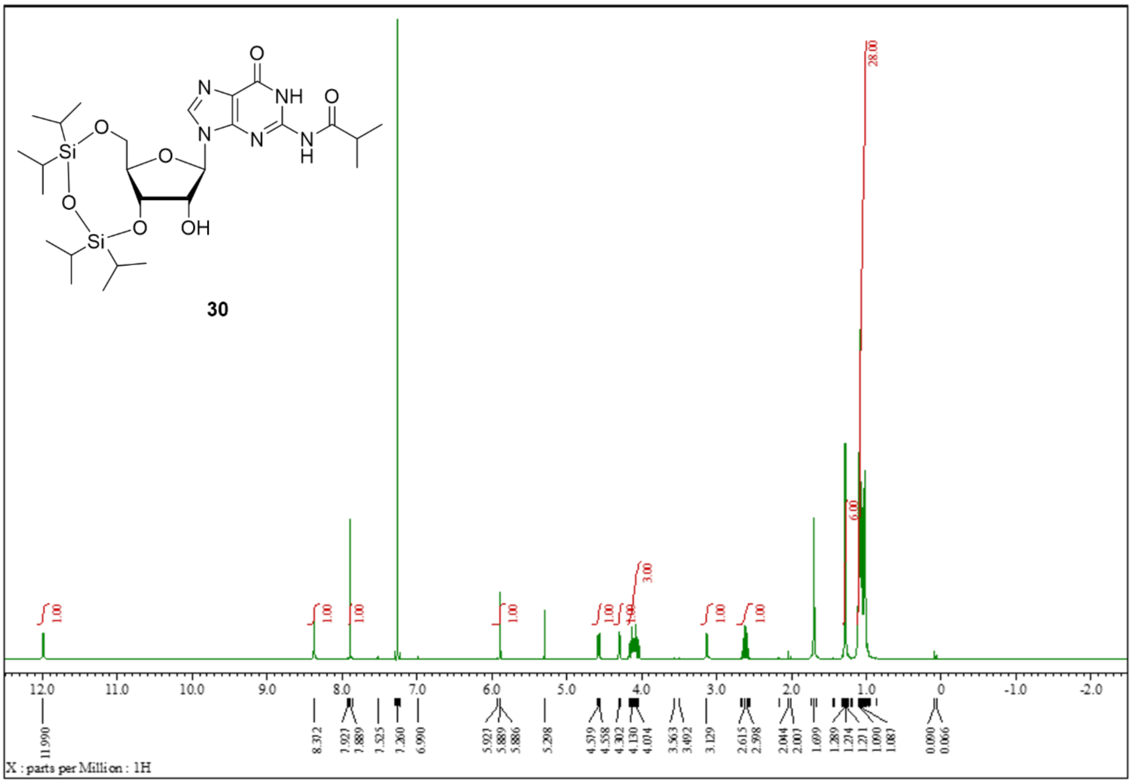

^{1}H-NMR (400 MHz, $CDCl_3$) of Compound **30**

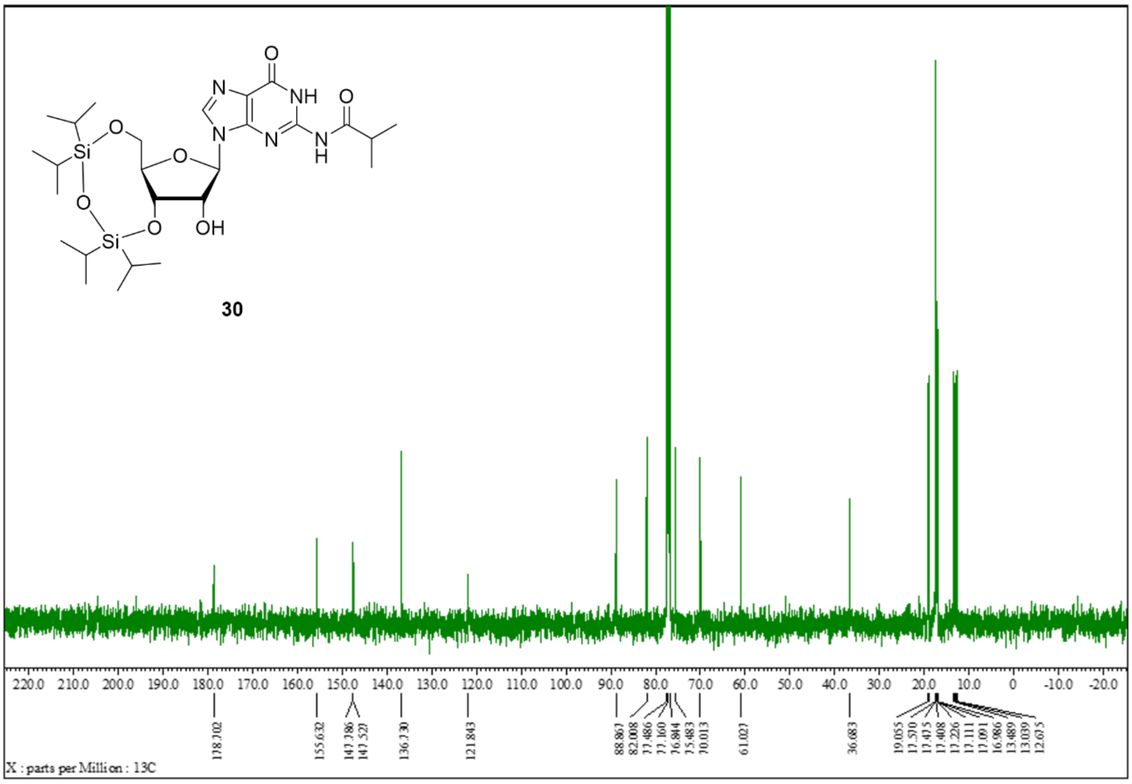

^{13}C-NMR (100 MHz, $CDCl_3$) of Compound **30**

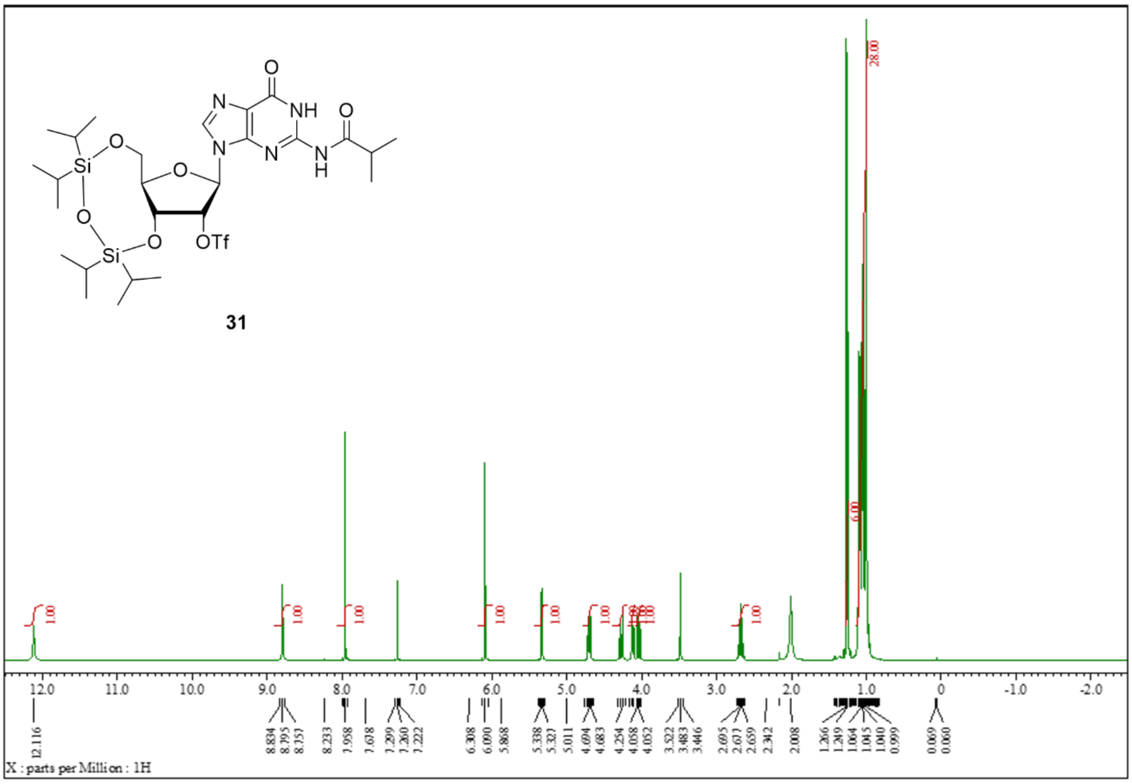

^{1}H-NMR (400 MHz, $CDCl_3$) of Compound **31**

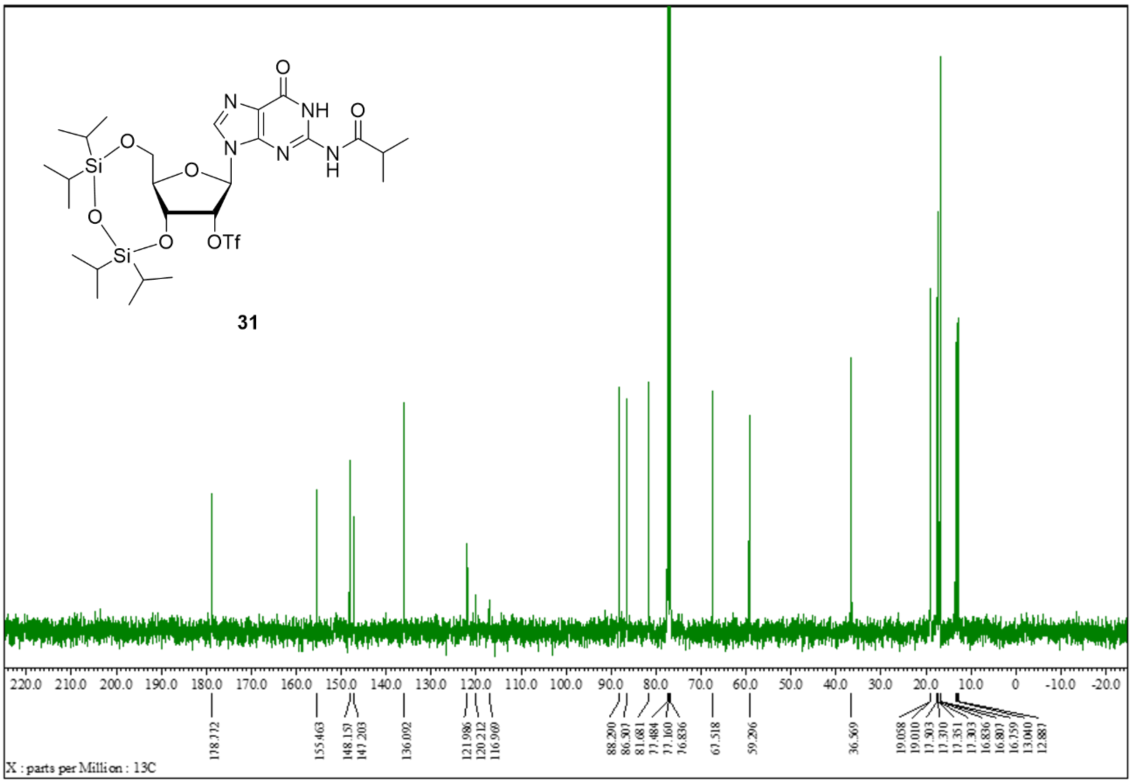

^{13}C-NMR (100 MHz, $CDCl_3$) of Compound **31**

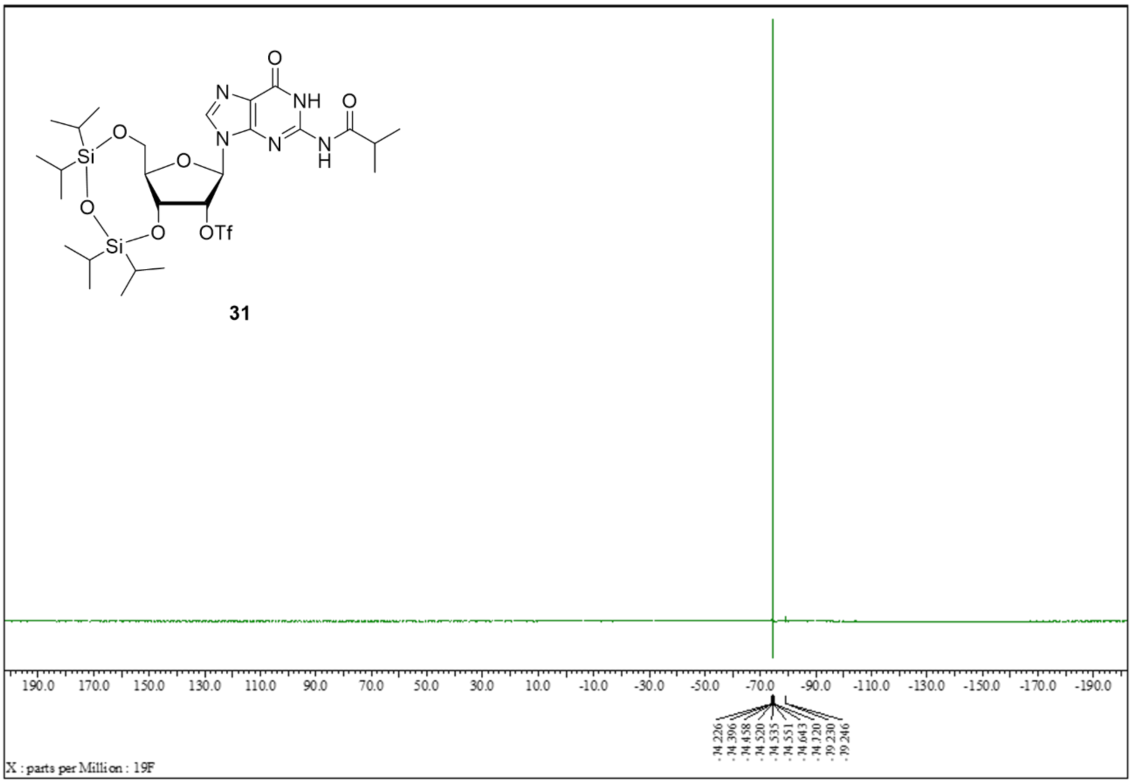

^{19}F-NMR (380 MHz, $CDCl_3$) of Compound **31**

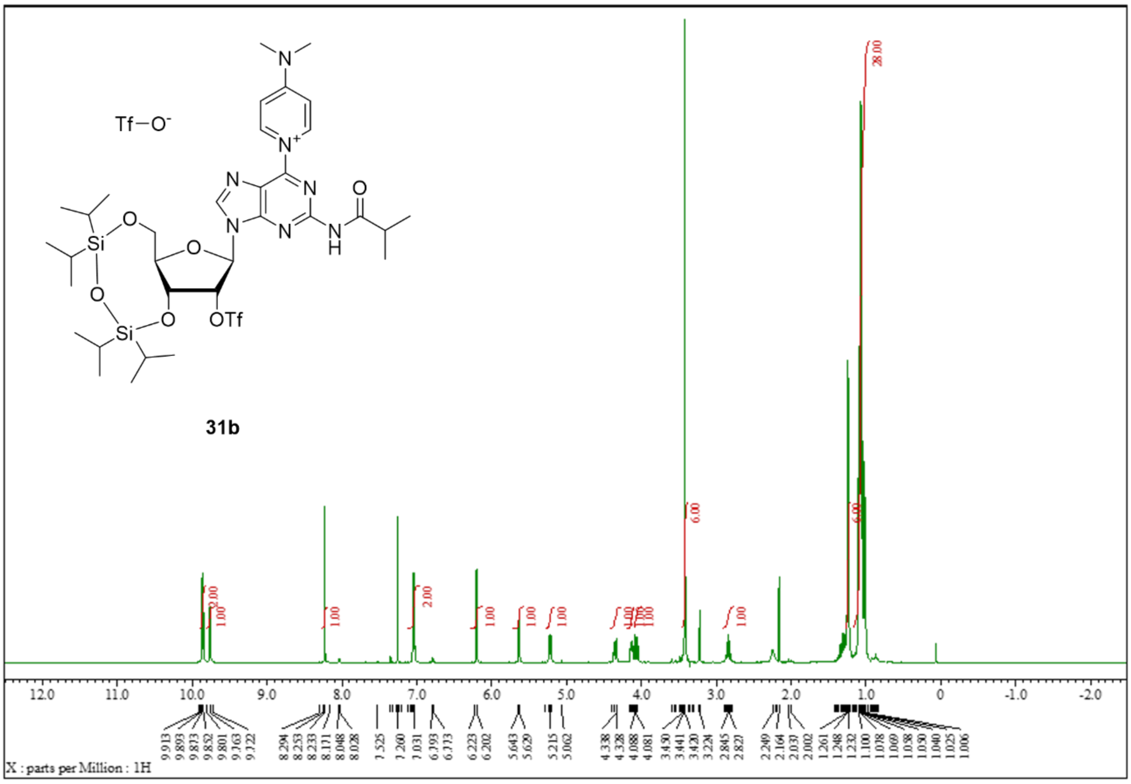

^{1}H-NMR (400 MHz, $CDCl_3$) of Compound **31b**

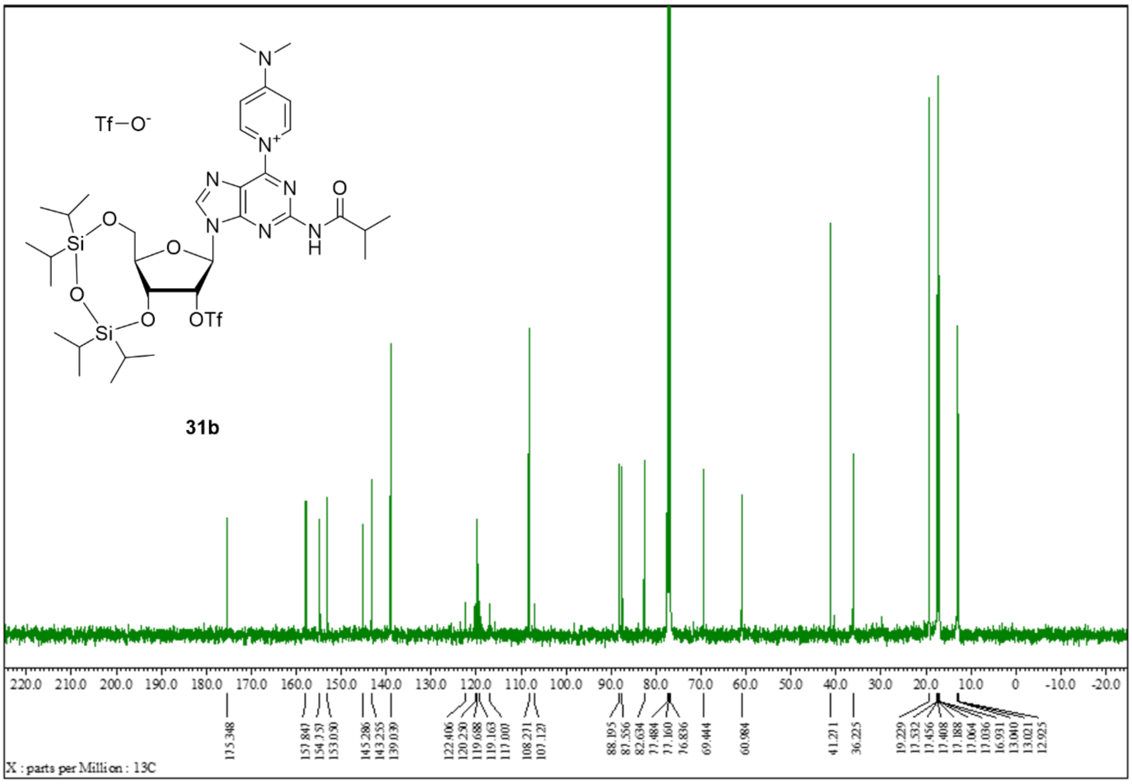

^{13}C-NMR (100 MHz, $CDCl_3$) of Compound **31b**

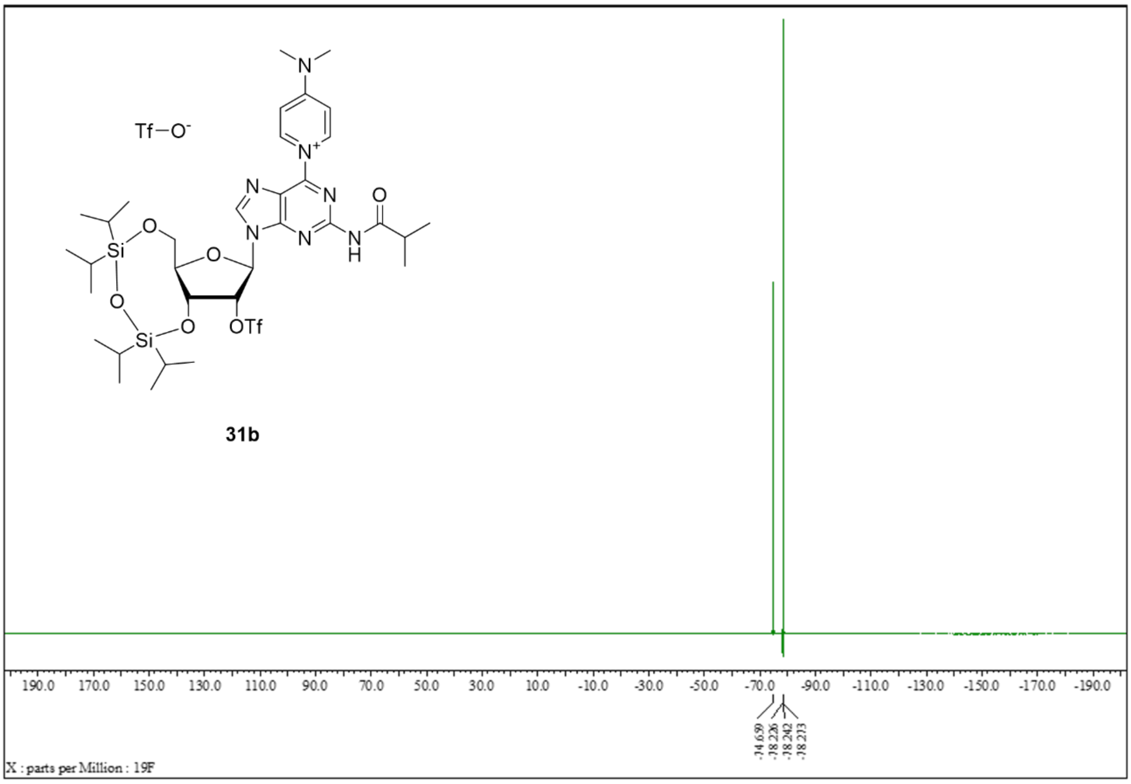

^{19}F-NMR (380 MHz, $CDCl_3$) of Compound **31b**

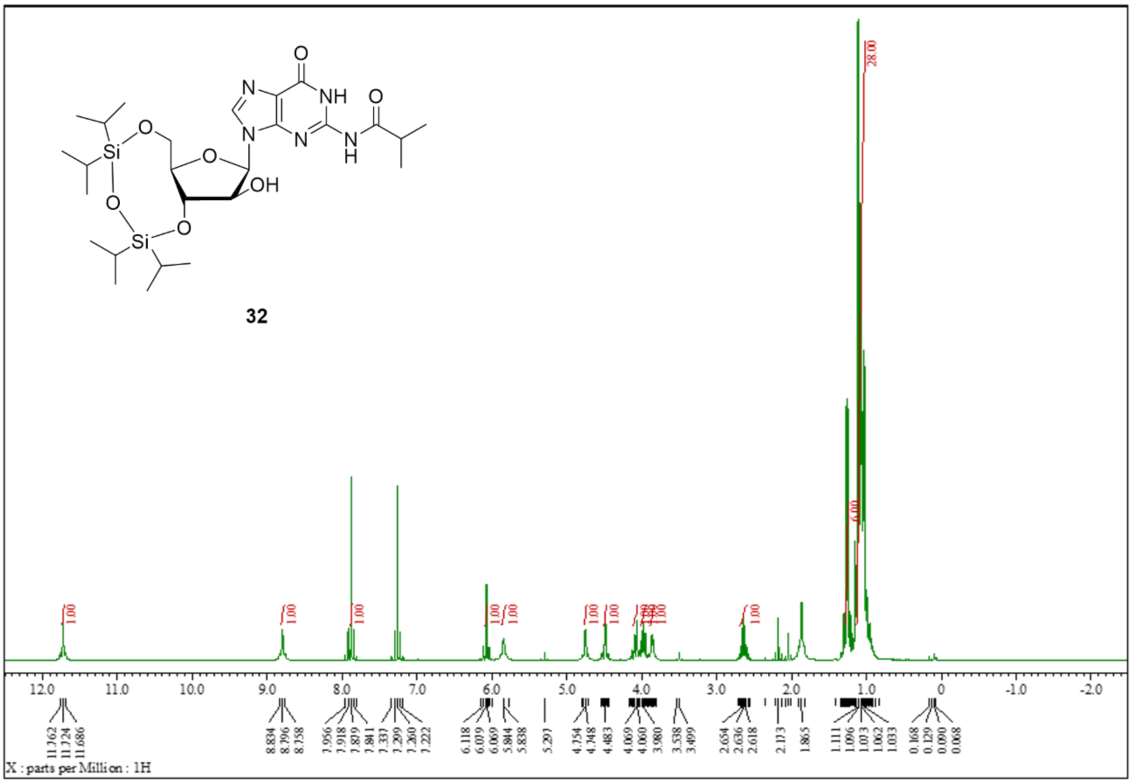

^{1}H-NMR (400 MHz, $CDCl_3$) of Compound **32**

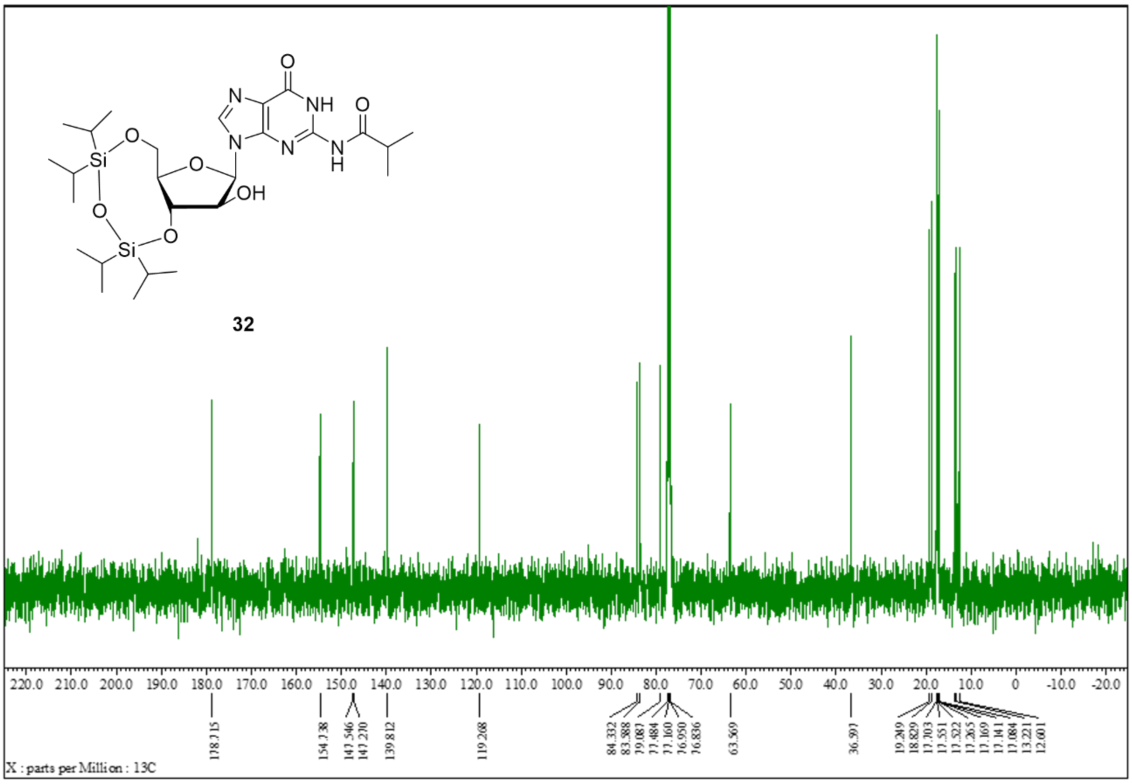

^{13}C-NMR (100 MHz, $CDCl_3$) of Compound **32**

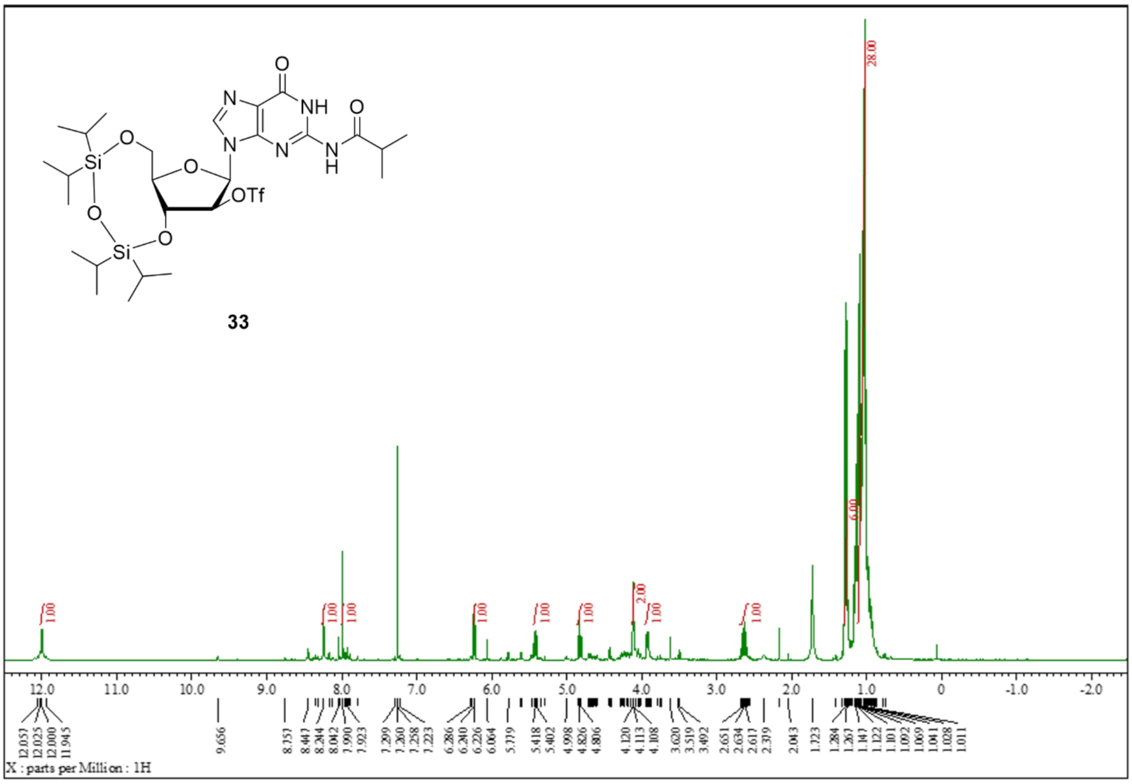

^{1}H-NMR (400 MHz, $CDCl_3$) of Compound **33**

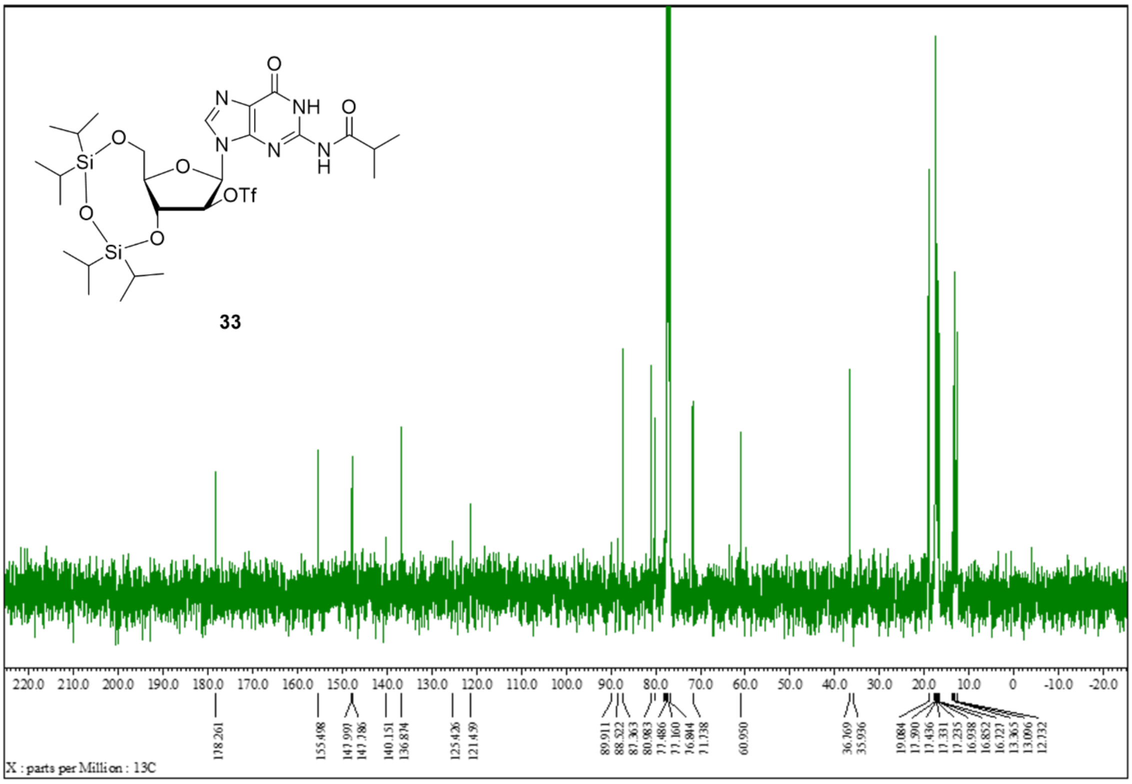

^{13}C-NMR (100 MHz, $CDCl_3$) of Compound **33**

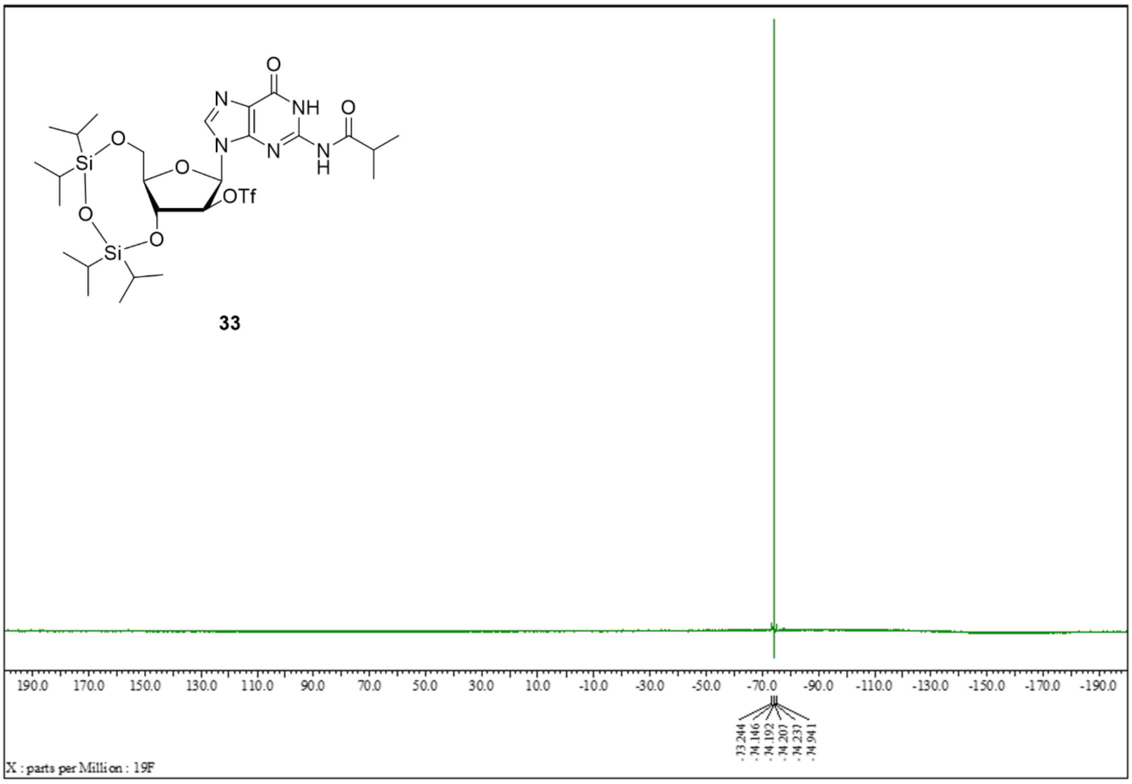

^{19}F-NMR (380 MHz, $CDCl_3$) of Compound **33**

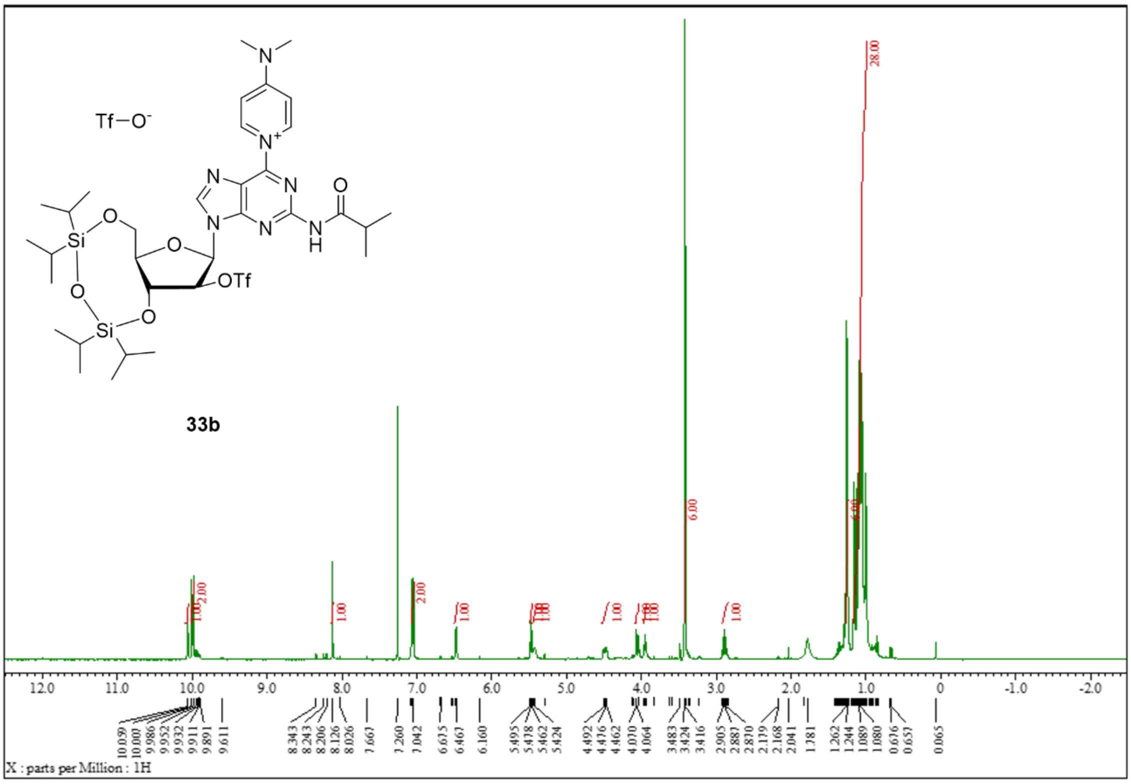

^{1}H-NMR (400 MHz, $CDCl_3$) of Compound **33b**

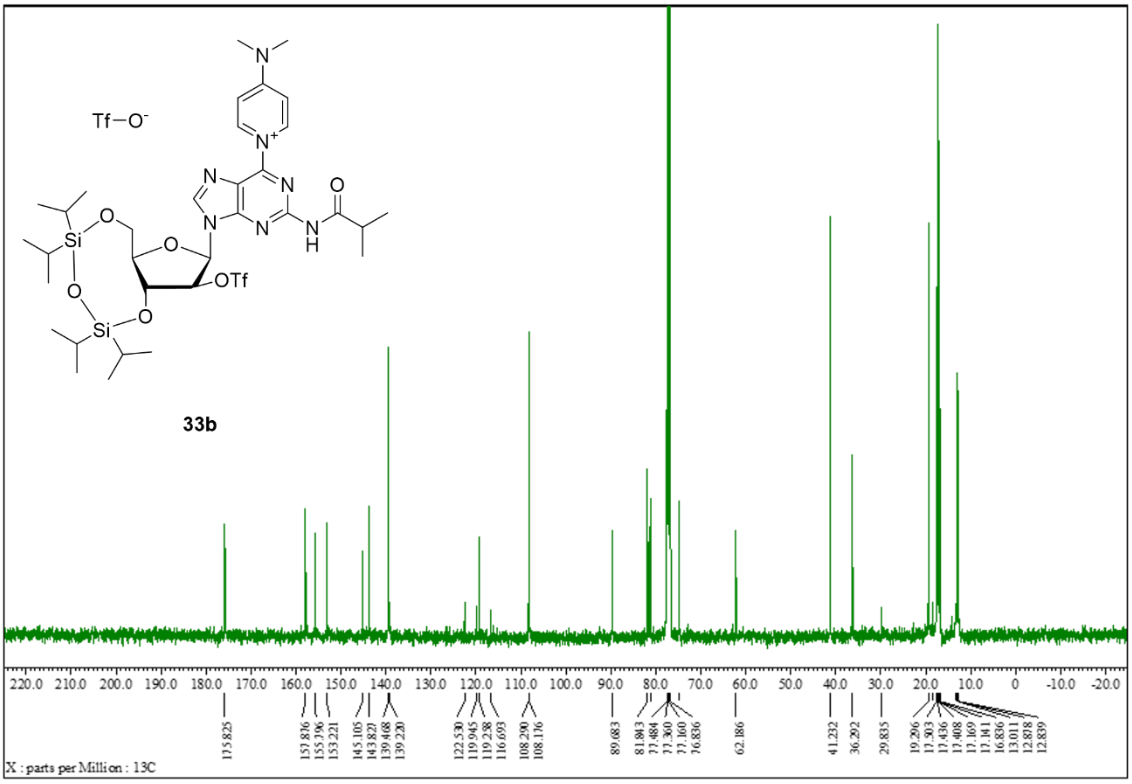

^{13}C-NMR (100 MHz, $CDCl_3$) of Compound **33b**

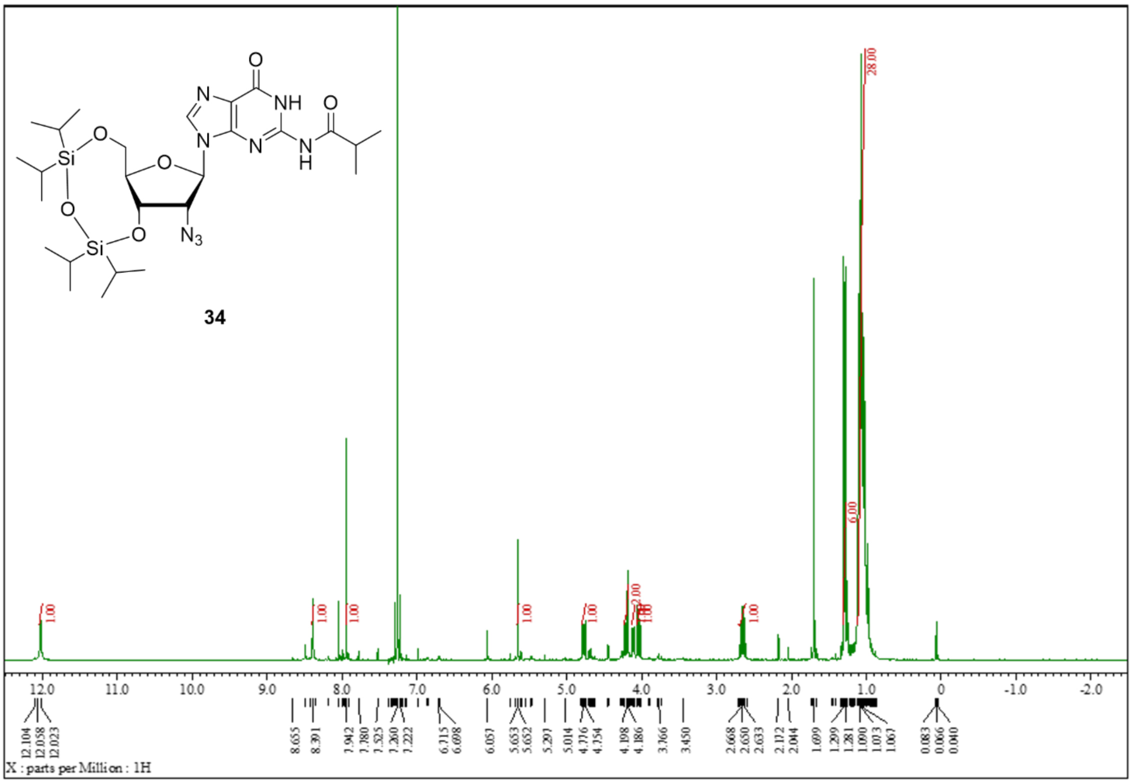

^{19}F-NMR (380 MHz, $CDCl_3$) of Compound **33b**

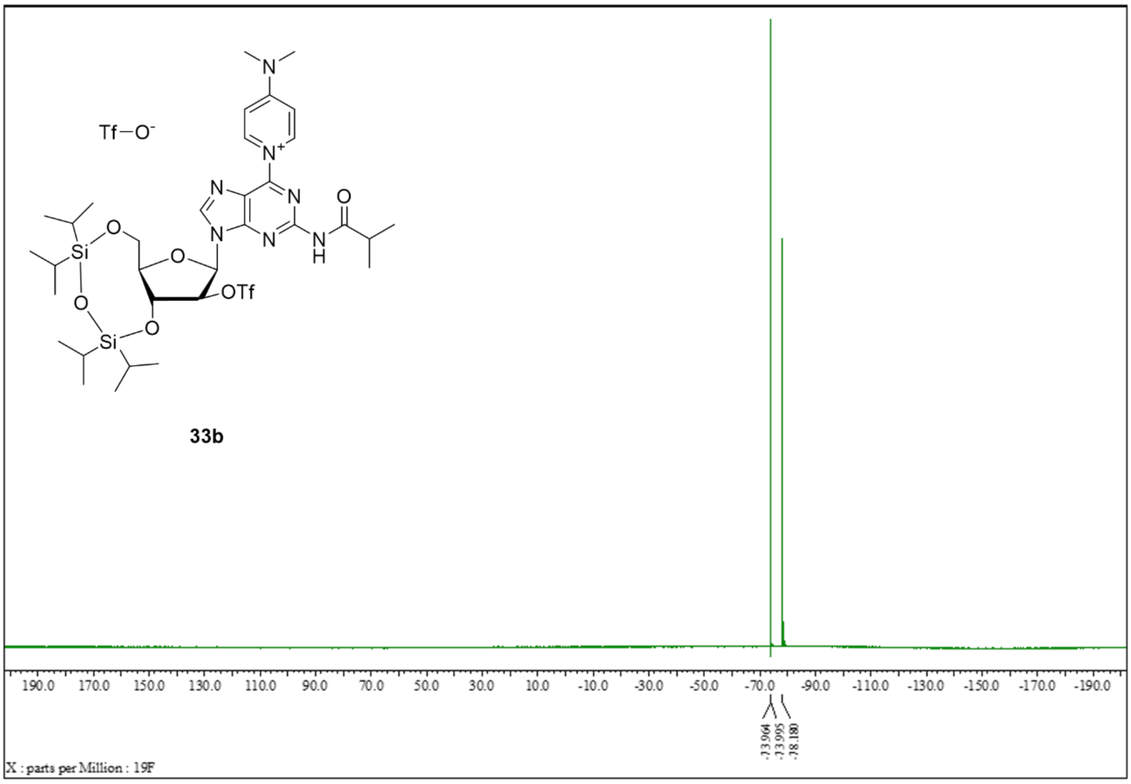

^{1}H-NMR (400 MHz, $CDCl_3$) of Compound **34**

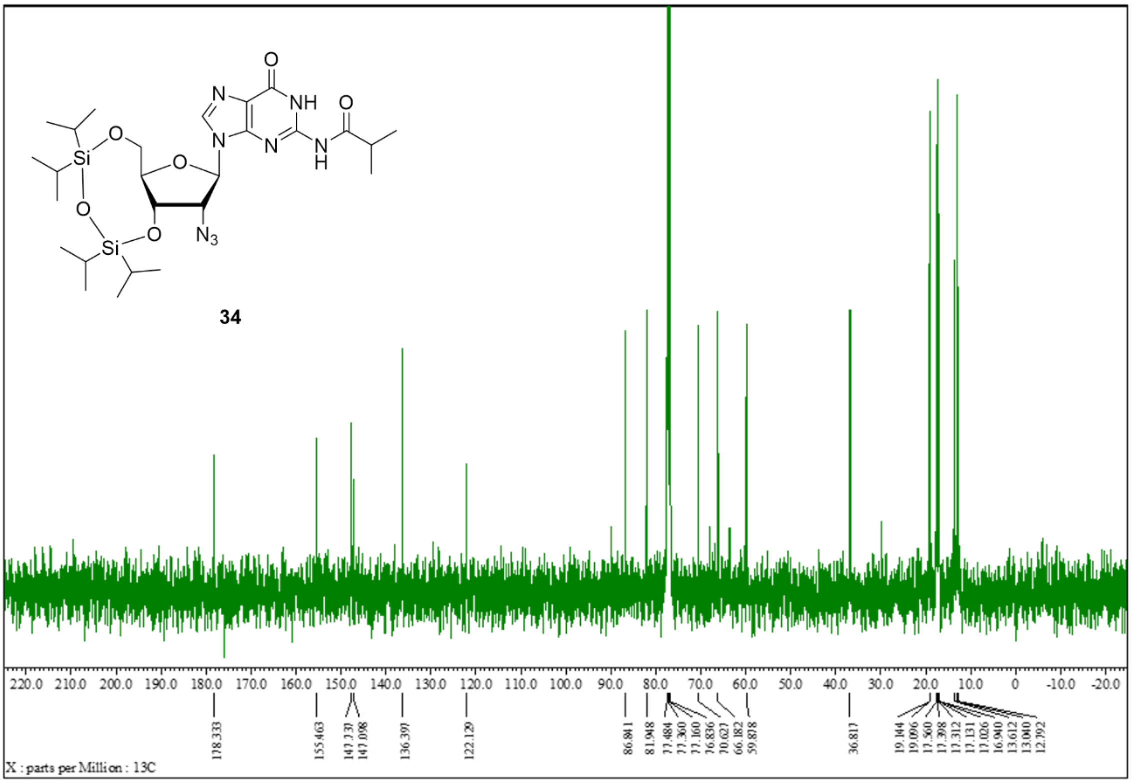

^{13}C-NMR (100 MHz, $CDCl_3$) of Compound **34**

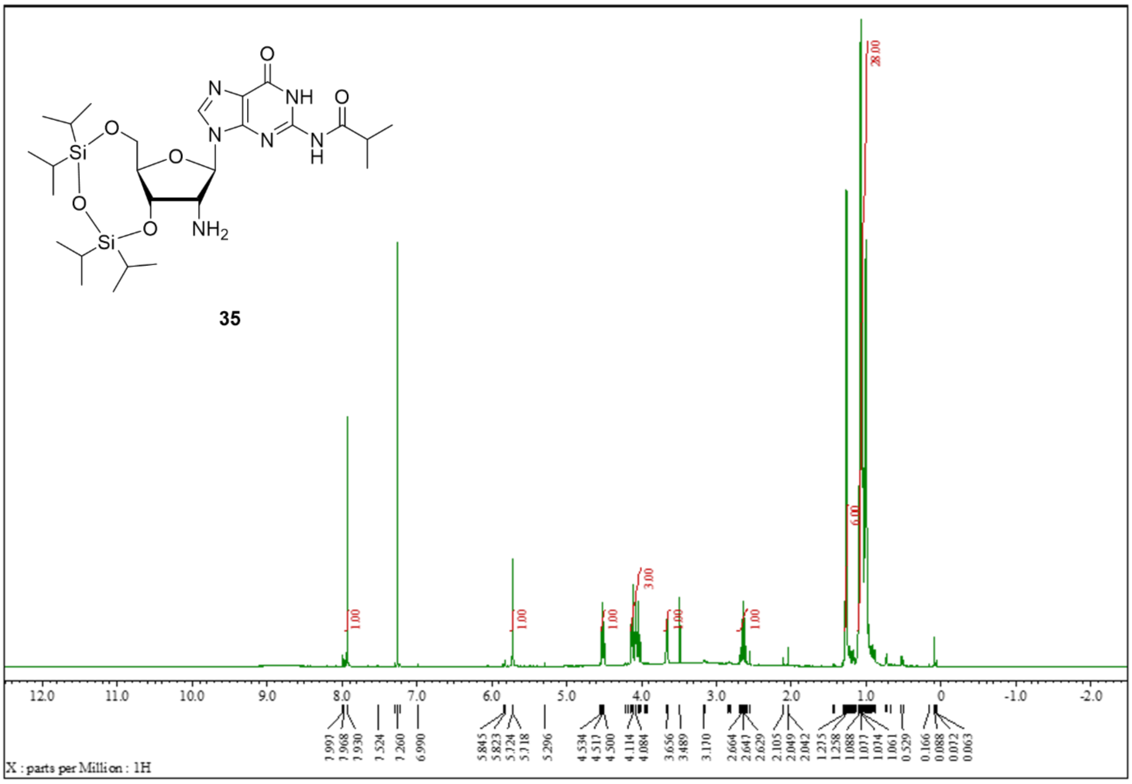

^{1}H-NMR (400 MHz, $CDCl_3$) of Compound **35**

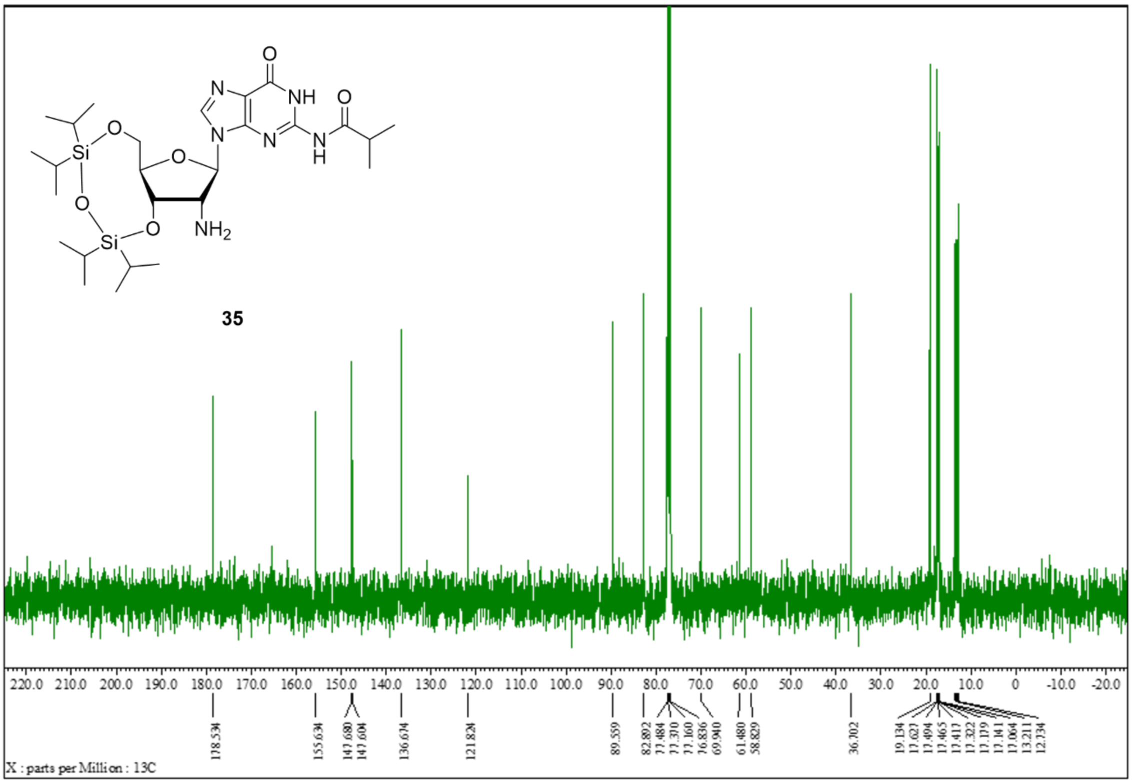

^{13}C-NMR (100 MHz, $CDCl_3$) of Compound **35**

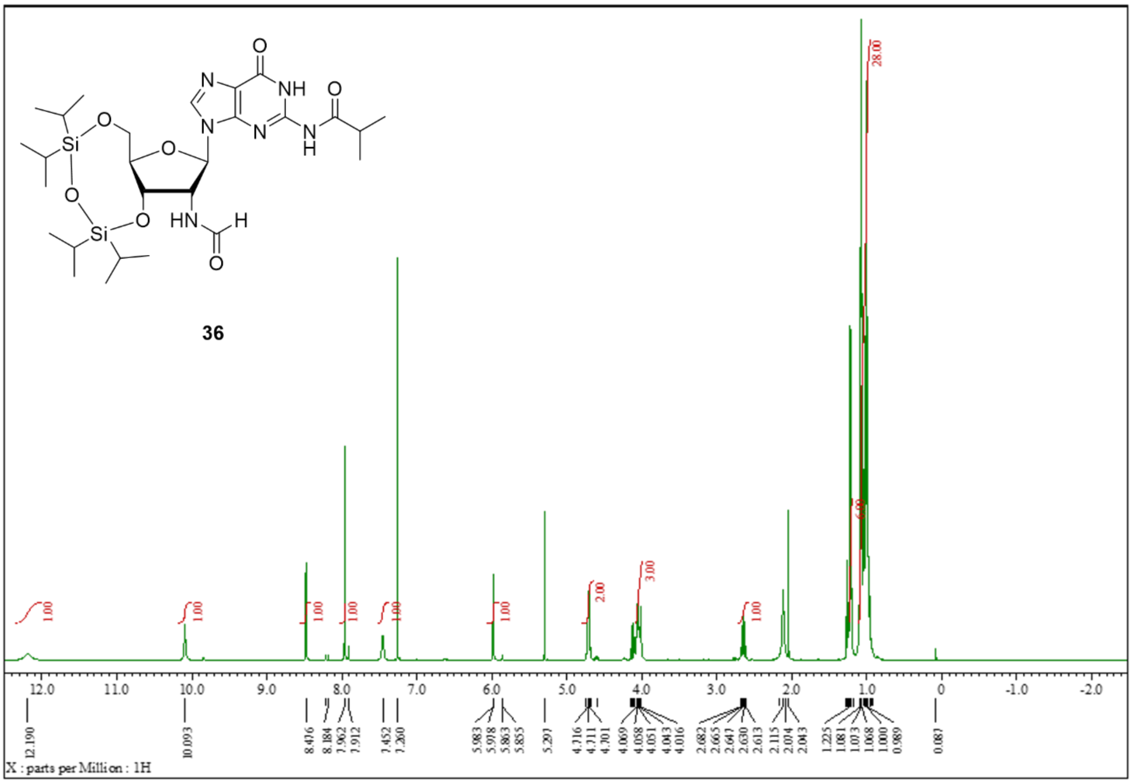

^{1}H-NMR (400 MHz, $CDCl_3$) of Compound **36**

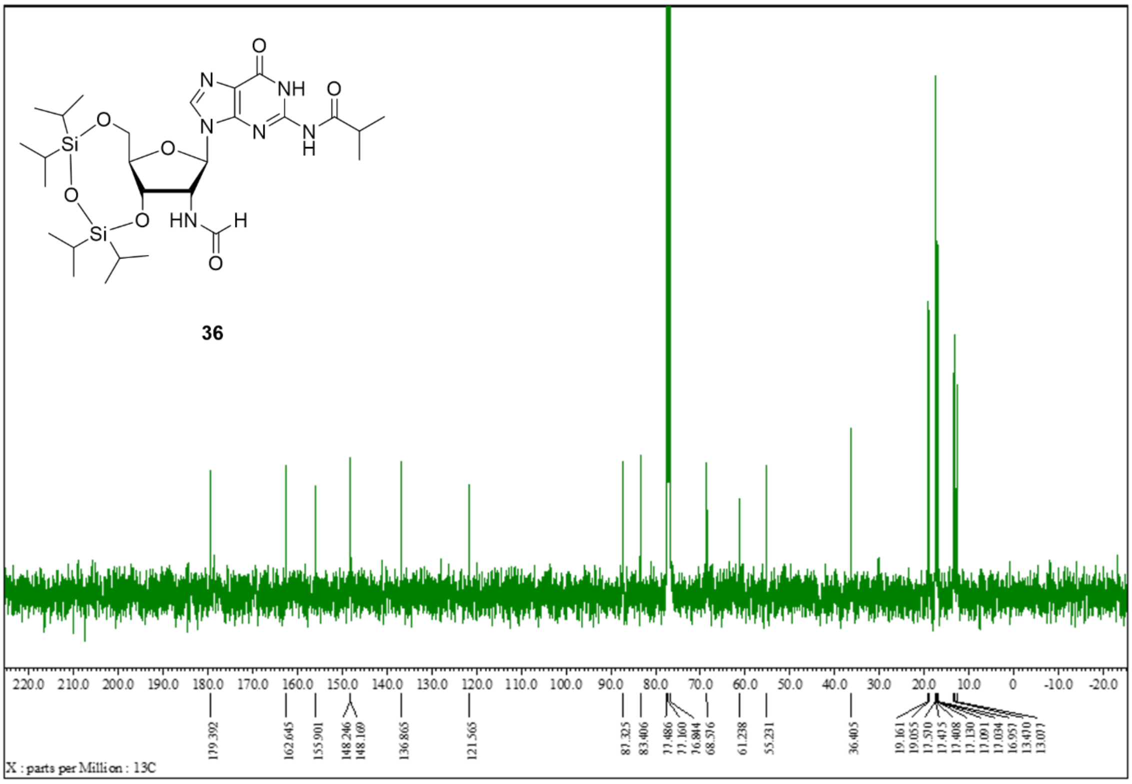

^{13}C-NMR (100 MHz, $CDCl_3$) of Compound **36**

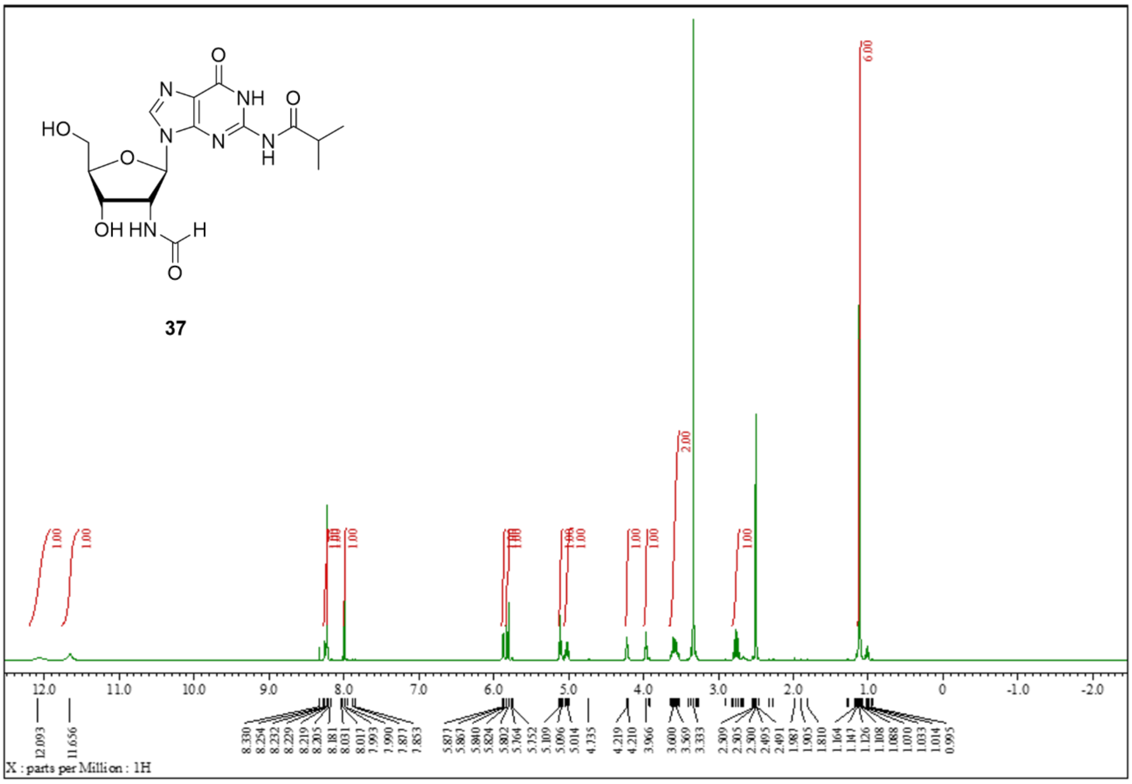

^{1}H-NMR (400 MHz, DMSO-d_6) of Compound **37**

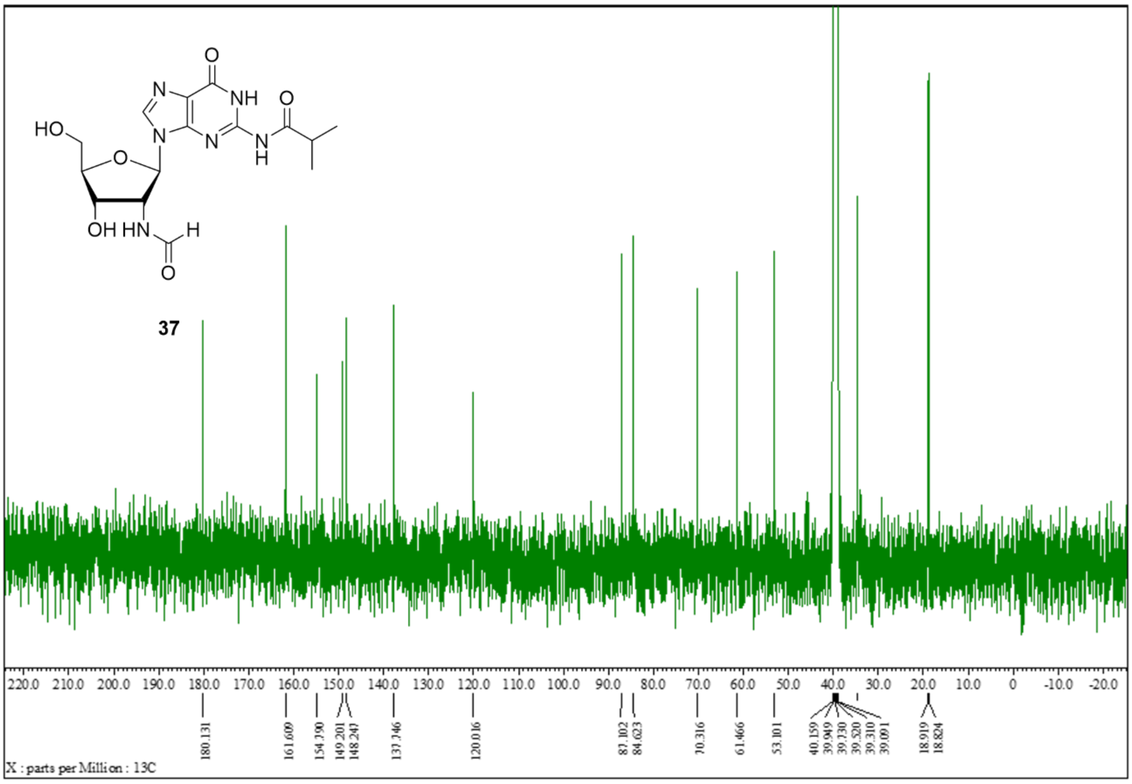

^{13}C-NMR (100 MHz, DMSO-d_6) of Compound **37**

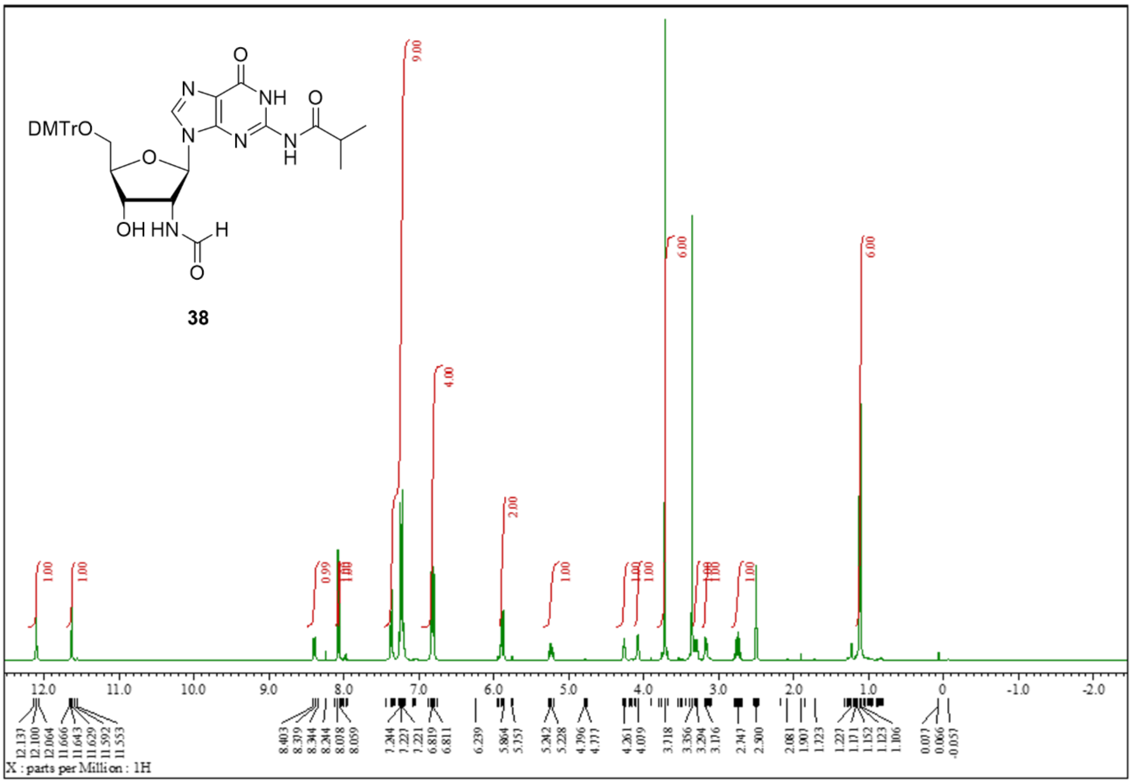

^{1}H-NMR (400 MHz, DMSO-d_6) of Compound **38**

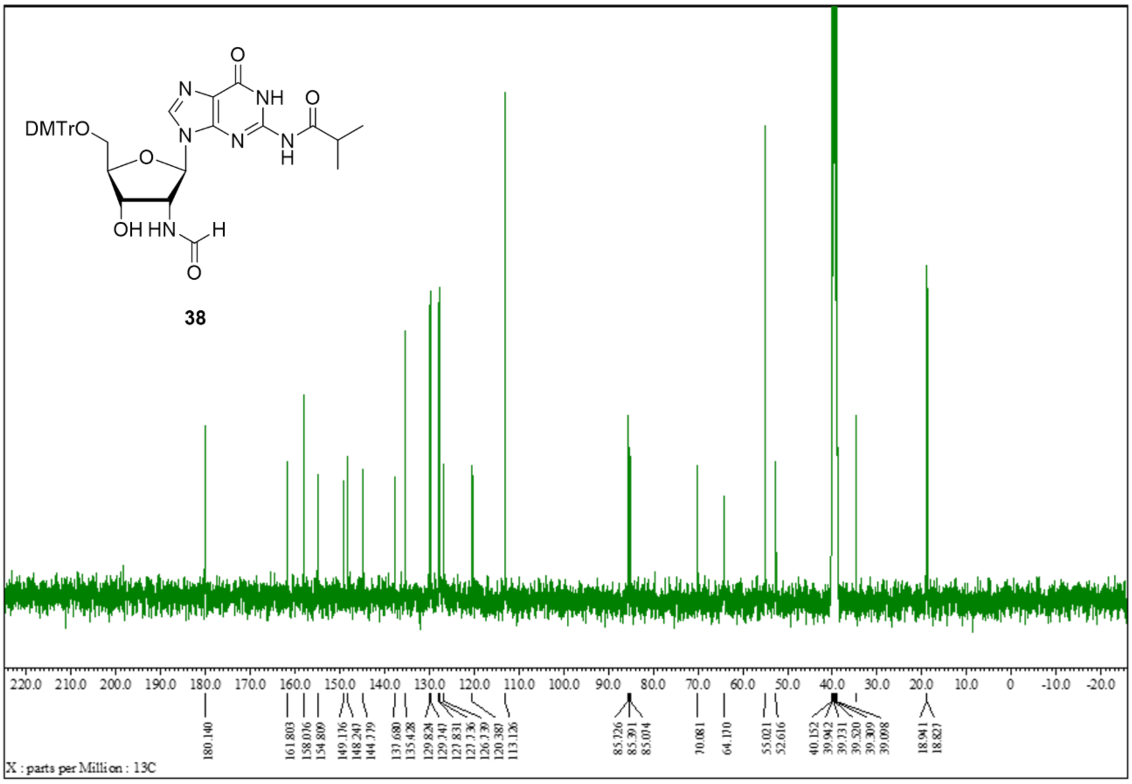

^{13}C-NMR (100 MHz, DMSO-d_6) of Compound **38**

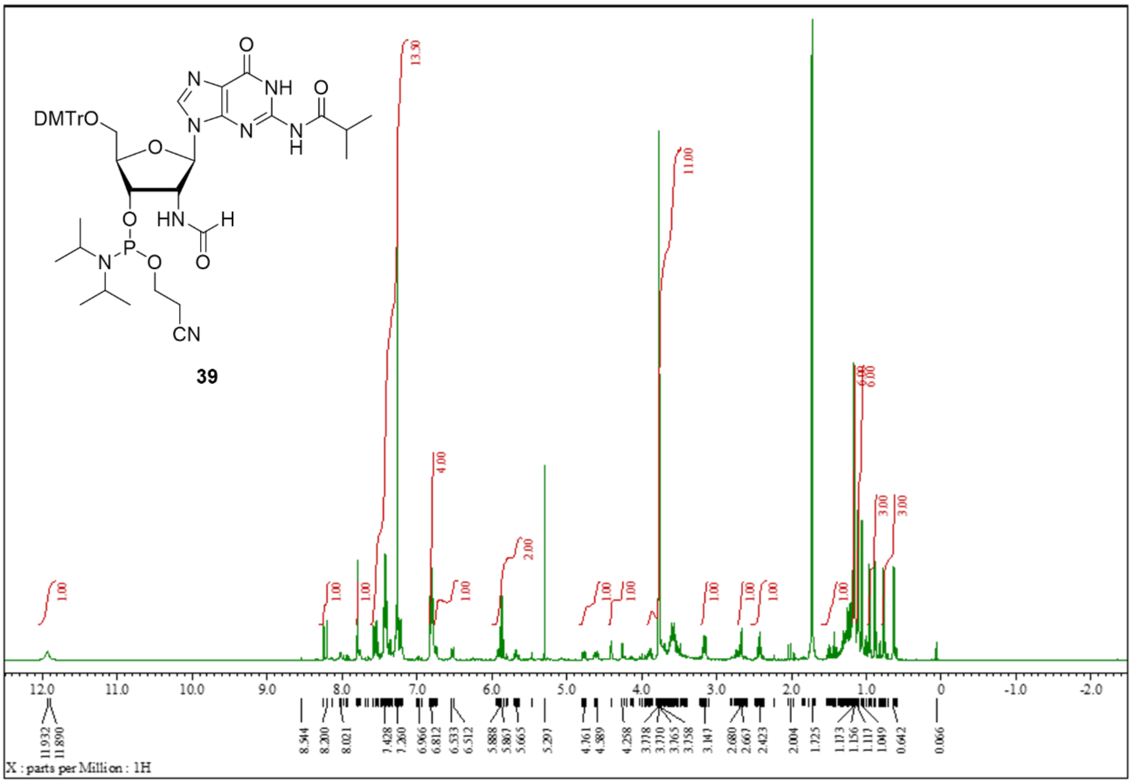

^{1}H-NMR (400 MHz, $CDCl_3$) of Compound **39**

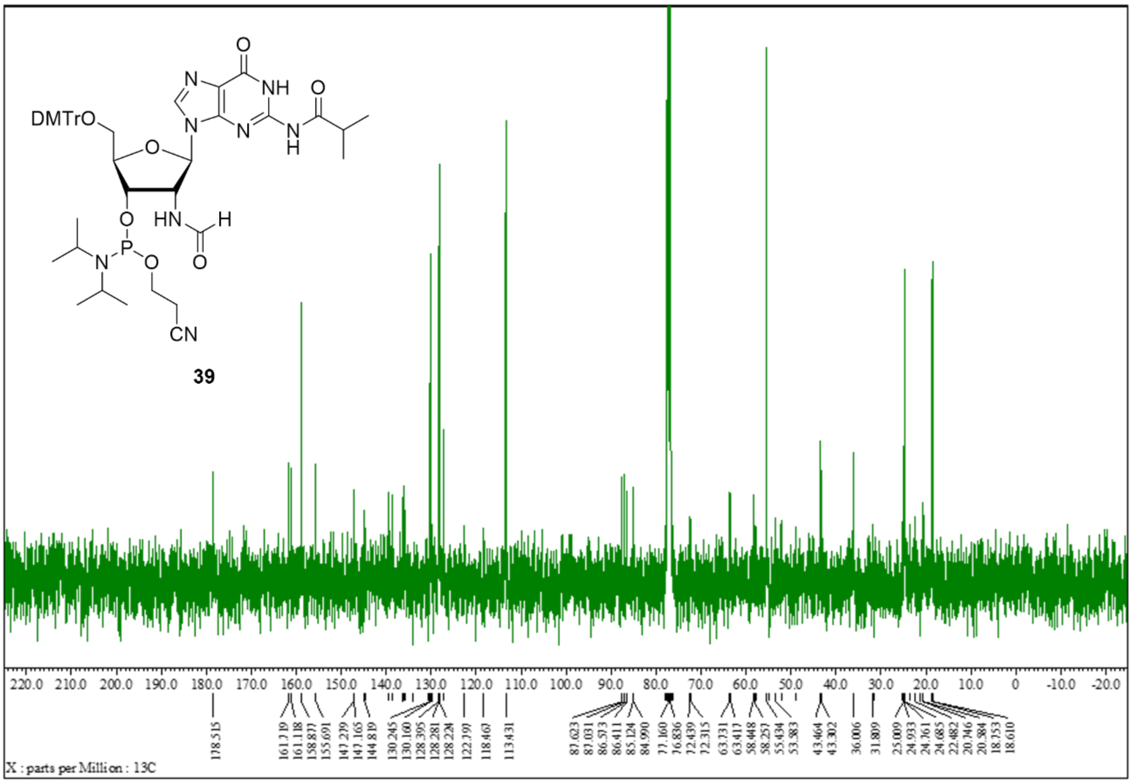

^{13}C-NMR (100 MHz, $CDCl_3$) of Compound **39**

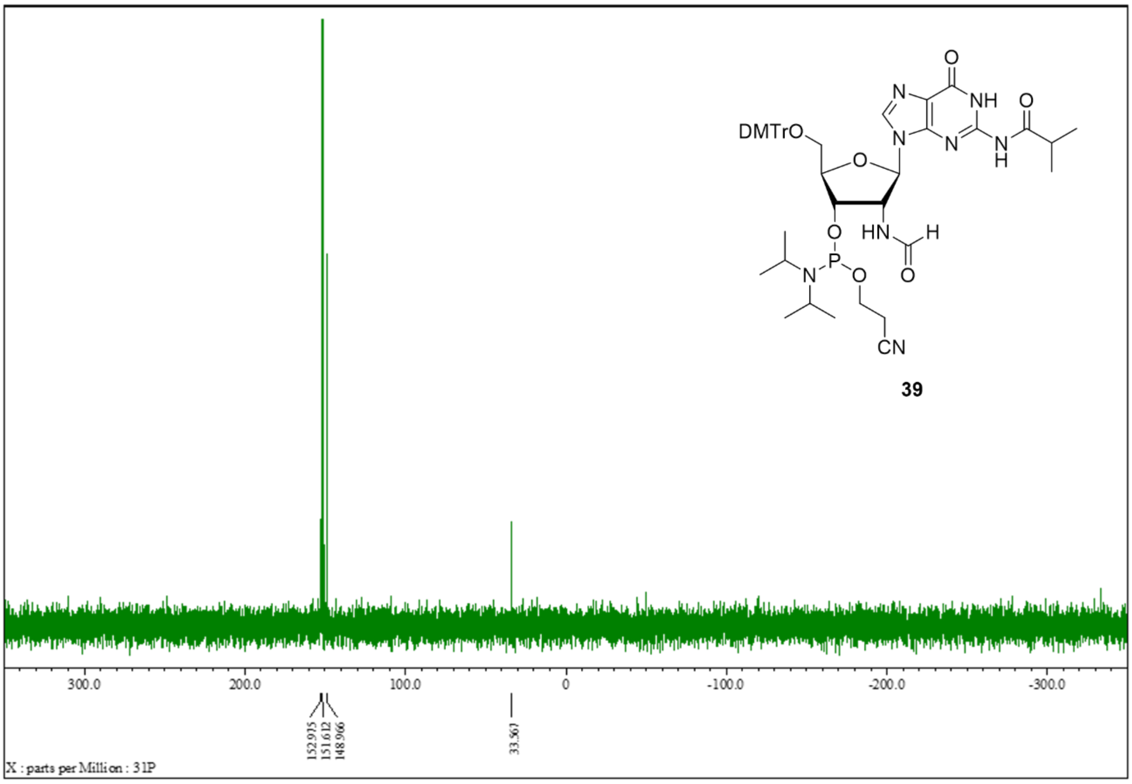

^{13}C-NMR (160 MHz, $CDCl_3$) of Compound **39**

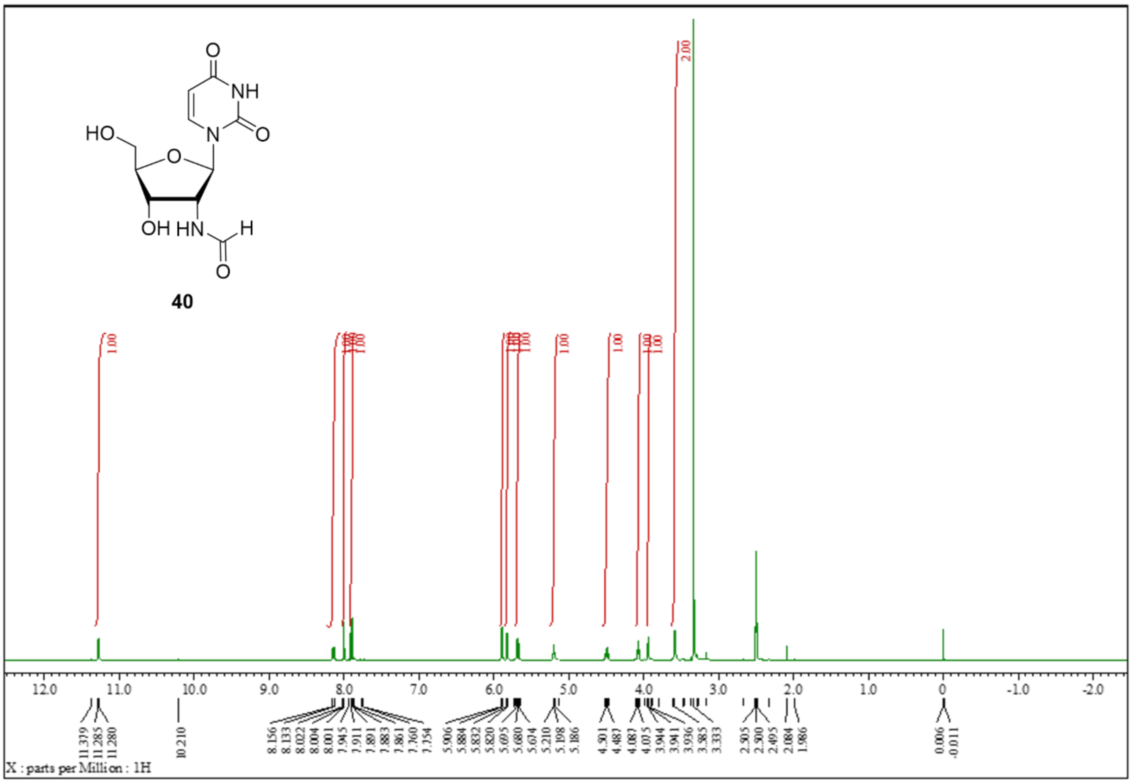

^{1}H-NMR (400 MHz, DMSO-d_6) of Compound **40**

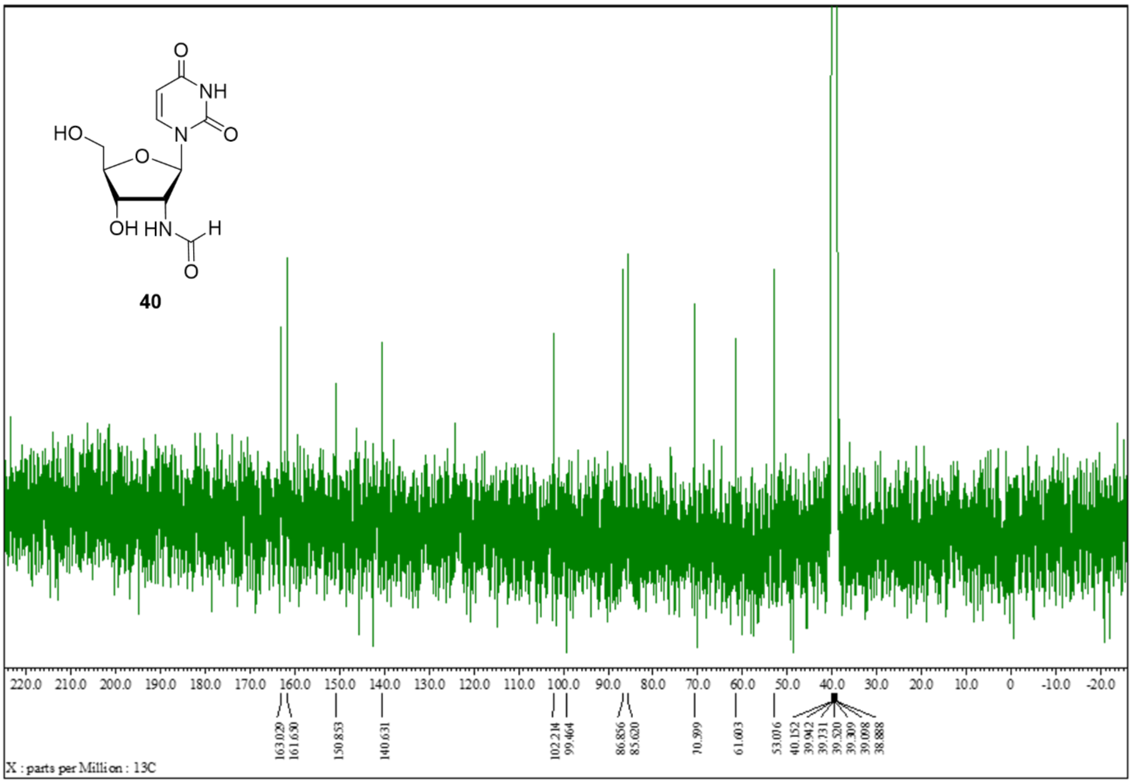

^{13}C-NMR (100 MHz, DMSO-d_6) of Compound **40**
